# Systematic analysis of CDR contacts and pairing constraints between T cell receptor *αβ* chains

**DOI:** 10.1093/bioinformatics/btag551

**Published:** 2026-07-22

**Authors:** Martina Milighetti, Yuta Nagano, James Henderson, Uri Hershberg, Andreas Tiffeau-Mayer, Anne-Florence Bitbol, Benny Chain

**Affiliations:** Division of Infection and Immunity, University College London, London, WC1E 6BT, United Kingdom; Cancer Institute, University College London, London, WC1E 6BT, United Kingdom; Division of Infection and Immunity, University College London, London, WC1E 6BT, United Kingdom; Division of Medicine, University College London, London, WC1E 6BT, United Kingdom; Division of Infection and Immunity, University College London, London, WC1E 6BT, United Kingdom; Institute for the Physics of Living Systems, University College London, London, WC1E 6BT, United Kingdom; Department of Human Biology, School of Life Sciences, Faculty of Sciences and Center of Biophysics and Quantitative Biology, University of Haifa, Haifa 3498858, Israel; Division of Infection and Immunity, University College London, London, WC1E 6BT, United Kingdom; Institute for the Physics of Living Systems, University College London, London, WC1E 6BT, United Kingdom; Institute of Bioengineering, School of Life Sciences, École Polytechnique Fédérale de Lausanne (EPFL), CH-1015 Lausanne, Switzerland; SIB Swiss Institute of Bioinformatics, CH-1015 Lausanne, Switzerland; Division of Infection and Immunity, University College London, London, WC1E 6BT, United Kingdom; Department of Computer Science, University College London, London, WC1E 6BT, United Kingdom

## Abstract

**Motivation:**

The six complementarity determining regions (CDRs) of the T cell receptor (TCR) form multiple contacts with cognate peptide and major histocompatibility complex, thus determining antigen specificity. However, the contacts between the CDRs themselves are less understood.

**Results:**

Our systematic study of all available TCR crystallographic structures identified consistent patterns of intra- and inter-chain CDR contacts in both free and antigen-bound TCRs. In addition, the protein sequences of TCRα and TCRβ from sets of TCRs which recognise a shared antigen shared mutual information and were not independent. As a result, sequence-based models can partially predict TCRα/TCRβ pairing *de novo*. The conserved patterns of CDR amino acid contacts, and the mutual sequence constraints between antigen-specific sets of TCR α and β chains represent an under-appreciated element of TCR structure, which may play an important role in T cell antigen recognition.

**Availability and implementation:**

The code and data necessary to reproduce the analyses are available at https://github.com/mm523/TCRab-pairing.

## 1 Introduction

The αβ T cell receptor (TCR) is a heterodimer which recognises peptides bound to major histocompatibility complex (MHC) molecules (pMHC). Each TCR chain is generated by somatic V(D)J recombination in each T cell between a Variable [V], a Joining [J] and, in the case of TCRβ, a Diversity [D] segment. The recombination generates high sequence diversity, which has been estimated to be greater than $10^{8}$ (Davis and Bjorkman 1988, Kesmir *et al*. 2000, Mora and Walczak 2019). TCRs bind pMHC through its complementarity-determining regions (CDRs or CDR1, CDR2 and CDR3; Szeto *et al*. 2020, Milighetti *et al*. 2021), which are the most variable regions of each TCR chain. CDR1 and CDR2 are encoded by the germline V gene, while CDR3 spans the recombination junction between V, D and J genes and is consequently the most diverse (Schwartz and Hershberg 2013). Notably, thousands of TCRs of different sequences can bind to the same pMHC (Dash *et al.* 2017, Glanville *et al.* 2017) and a single TCR may bind to many pMHCs (Sewell 2012).

Experimental studies have suggested that interactions between CDR loops might be important for epitope specificity, by for example affecting the inter-domain angle between the two TCR chains (McBeth *et al*. 2008). The interactions between CDRs may therefore constitute an important consideration in the design and optimisation of TCR-engineered T cells (Campillo-Davo *et al.* 2020, Baulu *et al*. 2023) or soluble TCRs (Robinson *et al*. 2021) for therapeutic applications. In this study we investigate the interaction between the TCR α and β chain CDR loops. We first systematically document the intra-chain and inter-chain contacts that the CDR loops make with each other. We then quantify the extent to which the CDR sequences of each chain constrain and hence predict the sequence of the other chain. If contacts between CDR loops are a conserved feature of TCRs and impact antigen binding, we expect the sequences of the two TCR chains to be interdependent in epitope-specific but not unselected repertoires. Since TCR repertoire data is often single chain only, the extent to which information on one chain predicts the sequence of the other is an important practical question, as is quantifying the extent to which the sequence of one chain determines specificity. Paired TCRαβ have been shown to carry more information about epitope specificity than each chain independently (Carter *et al*. 2019, Springer *et al*. 2021, Mayer and Callan 2023, Henderson *et al.* 2024), although the increase from adding the paired chain varies for different epitopes. We quantify the interchain constraints using a mutual information (MI) framework, and then use models originally developed in the context of protein co-evolution to predict chain pairing.

## 2 Methods

### 2.1 Analysis of existing crystal structures

A list of crystal structures for unbound TCRs and TCR-pMHC complexes was obtained from The Structural T-Cell Receptor Database (STCRDab, Leem *et al*. 2018), downloaded on 27 February 2023, *N = *918 structures). We selected all αβTCRs renumbered according to the International Immunogenetics Information System (IMGT, Lefranc *et al*. 2003), excluding all TCRs bound to non-canonical MHC (*N = *616 structures remaining). We removed any sequence with missing epitope information (*N = *590 remaining) and excluded non-peptide or chemically modified peptide antigens or superantigens, since their binding modalities may differ and are beyond the scope of this study (*N = *527 remaining). Where multiple structures were available from a single PDB, we chose the structure with the most complete chain combination (*N = *340 structures remaining). For the analysis of bound and unbound receptors, we extracted the sequences from the resulting 340 PDB files, and retained a non-redundant set of 157 structures of TCRs bound to cognate pMHC, and 78 unbound TCRs using STRCRDab annotations.

Structures were visualised using Pymol Open Source v2.5.0 (Schrödinger, LLC 2015).

To calculate intra-chain and inter-chain distances between two residues, we calculated the Euclidean distance between each pair of non-H atoms and retained the minimum distance across all atom pairs using the BioPDB package in Python v3.11 (Hamelryck and Manderick 2003). Contacts were defined as a minimum distance <5Å between two residues. A number of alternative thresholds were evaluated and 5Å was chosen as the average number of contacts between CDRs forms an elbow around the 5Å threshold (Fig. S1, available as supplementary data at *Bioinformatics* online).

For the epitopes analysed, we retrieved the crystal structures of pMHC complexes from the Protein DataBank (PDB), where available. Solvent-accessible surface area (SASA) for each epitope was calculated using Pymol Open Source v2.5.0 (Schrödinger, LLC 2015, command get_area on the peptide chain with dot_solvent 1 and solvent radius 1.4Å) on a representative structure for each epitope (Table 1).

**Table 1:** List of epitopes for which a PDB structure is available and calculated solvent-accessible surface area.

Epitope	PDB code	SASA	Reference
ASNENMETM	4HUX	311Å^2^	Valkenburg *et al.* (2013)
CINGVCWTV	3MRG	264Å^2^	Reiser *et al.* (2014)
ELAGIGILTV	1JF1	329Å^2^	Sliz *et al.* (2001)
GILGFVFTL	2VLL	259Å^2^	Ishizuka *et al.* (2008)
GLCTLVAML	3MRE	341Å^2^	Reiser *et al.* (2014)
LLWNGPMAV	5N6B	307Å^2^	Bovay *et al.* (2018)
LSLRNPILV	3BUY	308Å^2^	La Gruta *et al.* (2008)
NLVPMVATV	3GSO	375Å^2^	Gras *et al.* (2009)
RAKFKQLL	3SPV	243Å^2^	*To be published*
SSLENFRAYV	1WBY	464Å^2^	Meijers *et al.* (2005)
SSYRRPVGI	1WBZ	331Å^2^	Meijers *et al.* (2005)
YLQPRTFLL	7N6D	460Å^2^	Chaurasia *et al.* (2021)
SPRWYFYYL	7LGD	436Å^2^	Lineburg *et al.* (2021)

### 2.2 Paired-chain datasets

The VDJDb database (Goncharov *et al*. 2022) was exported on 3 February 2023. It was refined to only include TCRs for which both TCRα and TCRβ chains are available, and to include unique TCR-epitope pairs. We removed epitopes for which fewer than 100 (*N = *914) or more than 10 000 (*N = *1) sequences are available, to reduce skewing due to small samples and spurious annotations and be able to compare similarly-sized sets (Fig. S2A, available as supplementary data at *Bioinformatics* online). We chose the largest cut-off size which allowed us to retain enough epitopes for analysis and we also excluded very large sets for which computational time would have been unwieldy. stitchr (Heather *et al.* 2022) was used to obtain full chain sequences for each V/CDR3/J annotation for both mouse and human TCRs, and ANARCI (Dunbar and Deane 2016) was used to obtain IMGT-renumbered (Lefranc *et al*. 2003) CDR3 sequences from each chain. To ensure that CDR3 sequences were all the same length for MI calculations, sequences longer than 19 residues were discarded (Fig. S2B, available as supplementary data at *Bioinformatics* online), corresponding to <1% (67/9852) of all CDR3αβ. Sequences shorter than 19 were padded by addition of gaps at position $\frac{L}{2}$ (where *L* is the length of the CDR3 sequence). In order to allow within study randomisation for mutual information calculation (Appendix A2, available as supplementary data at *Bioinformatics* online), we restricted the analysis to studies which contain at least 3 sequences. tidytcells v1.6.0 (Nagano and Chain 2023) was used to standardise to the V and J genes, removing allele information. We further dropped duplicates in TRAV-CDR3A-TRAJ/TRBV-CDR3B-TRBJ pairs within each epitope repertoire after gene standardisation to avoid inflating the estimated mutual information (Appendix A2, available as supplementary data at *Bioinformatics* online, *N = *41 sequences removed). The final set of sequences comprised 9713 sequences from 22 epitopes (Table S1, available as supplementary data at *Bioinformatics* online). 7 of the epitopes and 4 of the TCRs (defined at the level of CDR3α-CDR3β) in this set are shared with the set of crystal structures analysed. Only one of the 9713 TCR-epitope pairs overlaps with the TCR-pMHC complexes included in the structural analysis.

Pre-processed TCR sequencing data from Tanno *et al.* (2020) was retrieved and further processed as in Mayer and Callan (2023). The clonotypes from sample A1, sorted on CD4${\;}^{+}$CD45RA${\;}^{+}$CCR7${\;}^{+}$ and classified by the authors as naïve cells were used as a control unselected repertoire. As for the VDJDb set, a length of 19 was enforced on the CDR3s.

We downloaded all human data from The Observed T Cell Receptor Space (OTS) database (Raybould *et al*. 2024). We used the annotations as given by the authors to remove γδ T cells, thymocytes or Mucosal-Associated Invariant T (MAIT) cells, and samples that had <100 sequences. As above, a length of 19 was enforced on the CDR3s.

### 2.3 Calculating mutual information between TCR regions

To quantify how much restriction one region of the TCR poses on another, we calculated the mutual information (MI) between different TCR regions. For CDR3s, where the diversity is too large to calculate entropy using the full sequence as a single categorical variable, we used a pairwise approximation by calculating the MI between single CDR3 residues and either the V or J genes, or single residues in the paired CDR3. All MI estimations were corrected for small sample size bias and finite sampling, background entropy and batch effects. Full details on the MI calculation are in Appendix A2, available as supplementary data at *Bioinformatics* online. MI was calculated using scikit-learn v1.1.3 (Pedregosa *et al*. 2012).

### 2.4 Calculation of effective set size

We define effective set size as the number of distinct sequences present in a repertoire, accounting for repeated or similar sequences (Weigt *et al*. 2009, Bitbol *et al*. 2016). For each sequence (*S*) in a set of size *N*, the number of sequence neighbours within a similarity threshold are counted ($m_{S}$) and *S* is assigned a weight $w_{S}=\frac{1}{m_{S}+1}$. The total effective set size can be calculated as:


(1) $$N_{\text{eff}}=\sum_{S\in 1}^{N}w_{S}=\sum_{S\in 1}^{N}\frac{1}{m_{S}+1}$$

If all sequences are distinct and have no neighbours, $N_{\text{eff}}=N$, whilst if all sequences are neighbours of all other sequences $N_{\text{eff}}=1$. The diversity is calculated by Hamming distance on the padded CDR3 sequences, excluding the first 3 and the last 3 amino acids of the CDR3. We set the threshold of similarity to be up to Hamming distance 1 for CDR3α and CDR3β, and up to Hamming distance 2 for CDR3αβ (calculated as a sum of the distance over the two chains). The effective set size $N_{\text{eff}}$ is normalised by total repertoire size *N* for comparison across repertoires. The effective set size was not correlated to total repertoire size (Fig. S3, available as supplementary data at *Bioinformatics* online).

### 2.5 Pairing models

We adapted two previously-published pairing algorithms to pair TCRαβ chains: the mutual information iterative pairing algorithm (MI-IPA, Bitbol 2018) and the graph alignment algorithm (GA, Bradde *et al*. 2010). The two methods perform pairing by either maximising the residue-level MI, or the alignment of similarity graphs of CDR3α and CDR3β sequences, respectively. These models were originally developed to leverage two distinct signals in co-evolving protein families: the co-evolution of specific residue positions (detected by MI) and the similarity in the phylogenetic trees of co-evolving protein families (detected in the graph alignment). We also evaluated a combination of the two algorithms (GA + MI-IPA, Gandarilla-Pérez *et al*. 2023). Full details on the implementation of each algorithm are in Appendix A3, available as supplementary data at *Bioinformatics* online.

The MI-IPA, GA and GA + MI-IPA models for each epitope were run on a computer with Intel Xeon W-2295 × 36 processor with 128GB RAM. All models were run on each epitope-specific repertoire from VDJDb separately, without using a training set of known pairs, as would be the case if no information about pairing was available. We inputted the CDR3 sequences only. To equalize epitope-specific TCR set sizes and reduce computational load, we subsampled the three largest epitope sets to 700 sequences, matching the size of the fourth-largest epitope-specific set. The set used for each epitope is summarised in Table S1, available as supplementary data at *Bioinformatics* online. For all models, epitopes GLCTLVAML (EBV) and YLQPRTFLL (SARS-CoV-2) were used to find the optimal parameters.

To quantitatively compare pairing algorithms, we calculated the proportion of predicted pairs that are correct, i.e. they are paired as observed in the downloaded VDJDb set (precision). Importantly, there are repeats in the α and β chain sets which will influence this calculation (Fig. S4, available as supplementary data at *Bioinformatics* online).

### 2.6 Benchmarking the pairing models

To benchmark the performance of MI-IPA, GA and GA + MI-IPA, we predicted pairing using TULIP (Meynard-Piganeau *et al.* 2024) and SCEPTR (Nagano *et al*. 2025). TULIP prediction was performed using the available pre-trained model. For each epitope, we generated all possible CDR3α-CDR3β pairs available from each individual and predicted the probability of binding of each pair against the epitope. We then assigned αβ pairs with a greedy algorithm similar to that used for the MI-IPA. Briefly, for each individual, we selected the most likely pair (lowest score). We then removed the α and the β sequences from the set, assigned the best remaining pair and continued iteratively until all pairs were assigned. We calculated precision as above.

To assign pairing with SCEPTR, we embedded the α and the β chains separately using the pre-trained default model. Since the training is performed by masking sections of the TCR, we expect that αβ paired chains may have similar embeddings. Therefore, we calculated the Euclidean distance between all α and all β chains from one individual for each epitope and then assigned pairs with the same greedy algorithm as above (starting with the pair with the smallest distance) and calculated precision as previously defined. SCEPTR was run both with its default model (trained on paired data) and with a model trained on synthetic data, in which the pairing in the training set was random (SCEPTR—synthetic).

## 3 Results

### 3.1 Contacts between CDR loops in existing TCR structures

**Figure 1:**
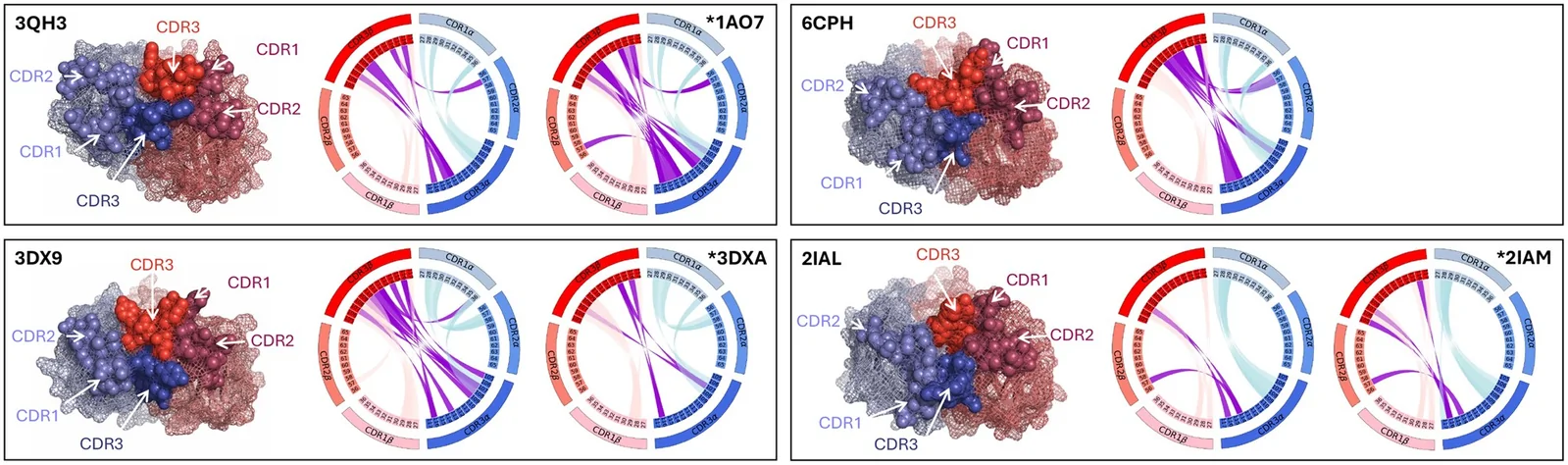
CDR loops come together to form the TCR binding site in four example TCRs. Positioning of CDR loops of 4 representative TCRs (2 recognise epitope on class I MHC, 3QH3 and 3DX9; 2 recognise epitope on class II MHC, 6CPH and 2IAL). The TCRα chain is in shades of blue, whilst the TCRβ is in shades of red. CDR1, 2 and 3 for each chain are represented with spheres and annotated on the figure. Mesh shows the rest of the TCR structure. On the right of each structure, the circle plot shows contacts between the CDR loops (defined as minimum distance between two non-H atoms on two residues <5Å) for the same TCR in both the unbound (left) and bound (right, indicated with a *) structure. Light blue: contacts between loops on TCRα; light red: contacts between loops on TCRβ; purple: contacts between the two chains. 3QH3: Scott *et al*. (2011), 1AO7: Garboczi *et al*. (1996), 3DX9 and 3DXA: Archbold *et al*. (2009), 6CPH: Galperin *et al*. (2018), 2IAL and 2IAM: Deng *et al*. (2007). Structures are plotted using Pymol Open Source (Schrödinger, LLC 2015). Circos plots are plotted using Python package pyCirclize (Shimoyama 2022). CDR, Complementarity-Determining Region.

One type of interaction between CDR loops may be mediated by molecular contacts between amino acids. We therefore examined 340 curated TCR-pMHC structures available from the The Structural T-Cell Receptor Database (STCRDab, Leem *et al*. 2018). We defined contact as minimum distance between two non-H atoms <5Å. Observation of 4 representative TCRs (Fig. 1) shows that CDR loops form a continuous contact surface that binds pMHC. There are both intra- (between two CDR residues on the same chain) and inter-chain (between one residue on the α and one residue on the β chain) contacts observed both in the bound (right panel, indicated with a *) and in the unbound (middle panel) TCR structures, although the number of contacts varies between the ligated and free receptors.

**Figure 2:**
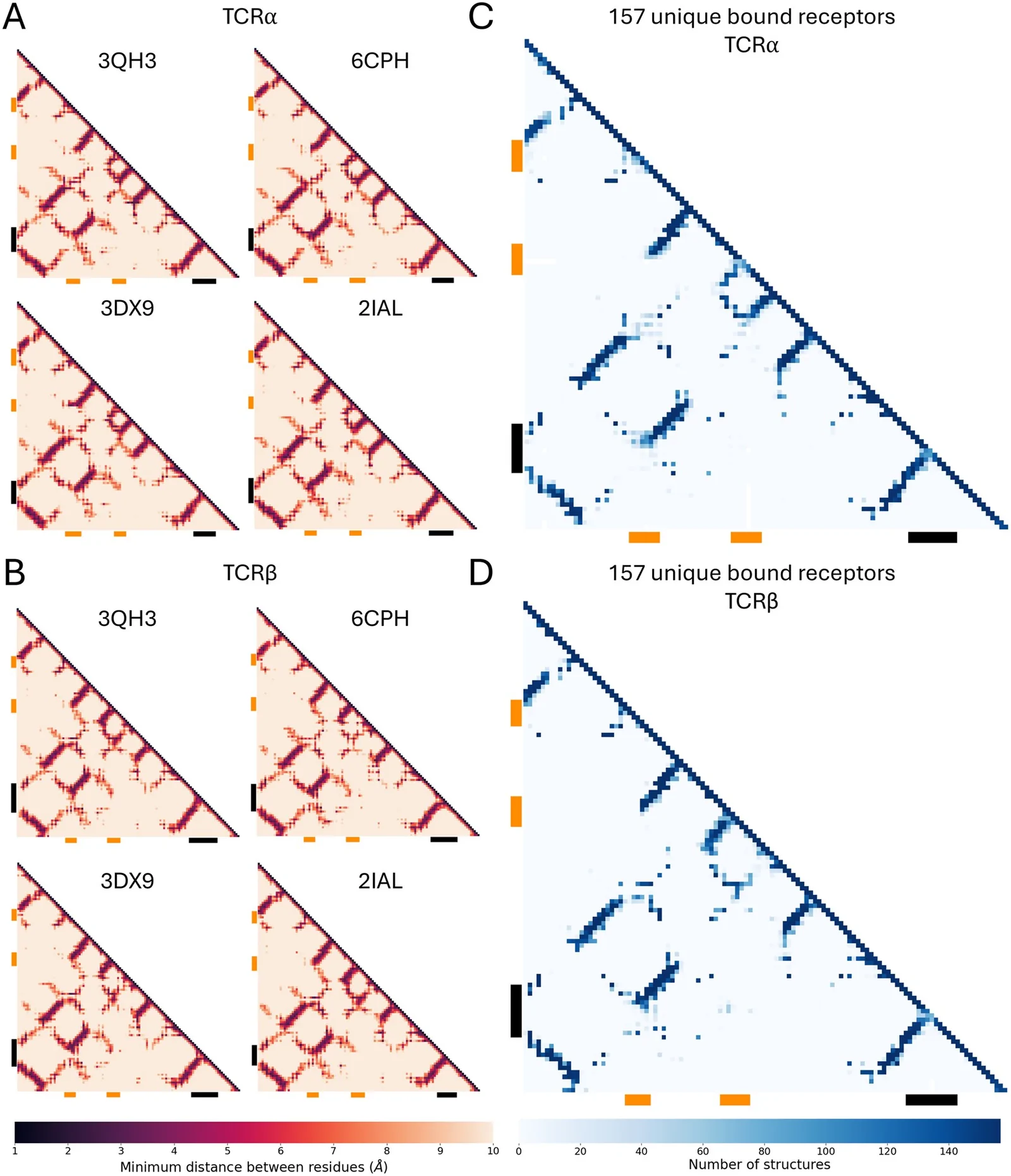
Intra-chain interactions are observed in bound and unbound TCR structures. (A, B) Intra-chain contact maps for the unligated structures in Figure 1 for the TCR α and β chain, respectively. The distance (Å, colour bar) is calculated between all pairs of atoms in the two residues, and the minimum distance is shown. (C, D) Intra-chain contact maps were calculated for the TCR α and β chain (respectively) of 157 unique bound TCRs. A contact is defined as minimum distance between two non-H atoms on two residues <5Å. The heatmap shows the number of structures that are found to make contact at each pair of positions. Only positions present in >75% of structures according to the IMGT numbering scheme are shown. Only the bottom half of each distance matrix is shown as it is symmetrical across the diagonal. In each heatmap, the positions of CDR1, 2 (orange) and 3 (black) are highlighted.

Consistent with Fig. 1, the intra-chain distances for the four example unligated structures (Fig. 2A and B for TCRα and β, respectively) show intra-chain contacts between CDR1 and CDR3 and CDR1 and CDR2, both in the TCRα and in the TCRβ chain. The patterns are consistent across the four structures and in both chains. The same analysis was extended to 78 unique unbound receptors (Fig. S5A, available as supplementary data at *Bioinformatics* online), 157 unique pMHC bound TCRs (Figs 2C, D and S5B, C, available as supplementary data at *Bioinformatics* online) and TCRs that bind MHC with reversed polarity (Fig. S5D, available as supplementary data at *Bioinformatics* online). We also calculated the number of contacts between CDR loops, defined as minimum distance between two non-H atoms <5Å, for all 340 available structures (Fig. S6A, available as supplementary data at *Bioinformatics* online). The patterns observed in the 4 example structures are generalisable and observed in TCRs both in the unligated and ligated form. Strikingly, all 340 PDB files analysed show contacts between CDR1 and CDR3 on both chains (Fig. S6A, available as supplementary data at *Bioinformatics* online; median of 13 contacts [range 5–23] for TCRα and median of 14 [range 8–22] contacts for TCRβ). CDR2 in turn forms contacts with CDR1 (median of 5 contacts for both TCRα and TCRβ, ranges of 0–12 and 0–7, respectively). We also observe contacts between CDR2 and CDR3 (range 0–3 contacts in TCRα and 0–4 contacts in TCRβ).

**Figure 3:**
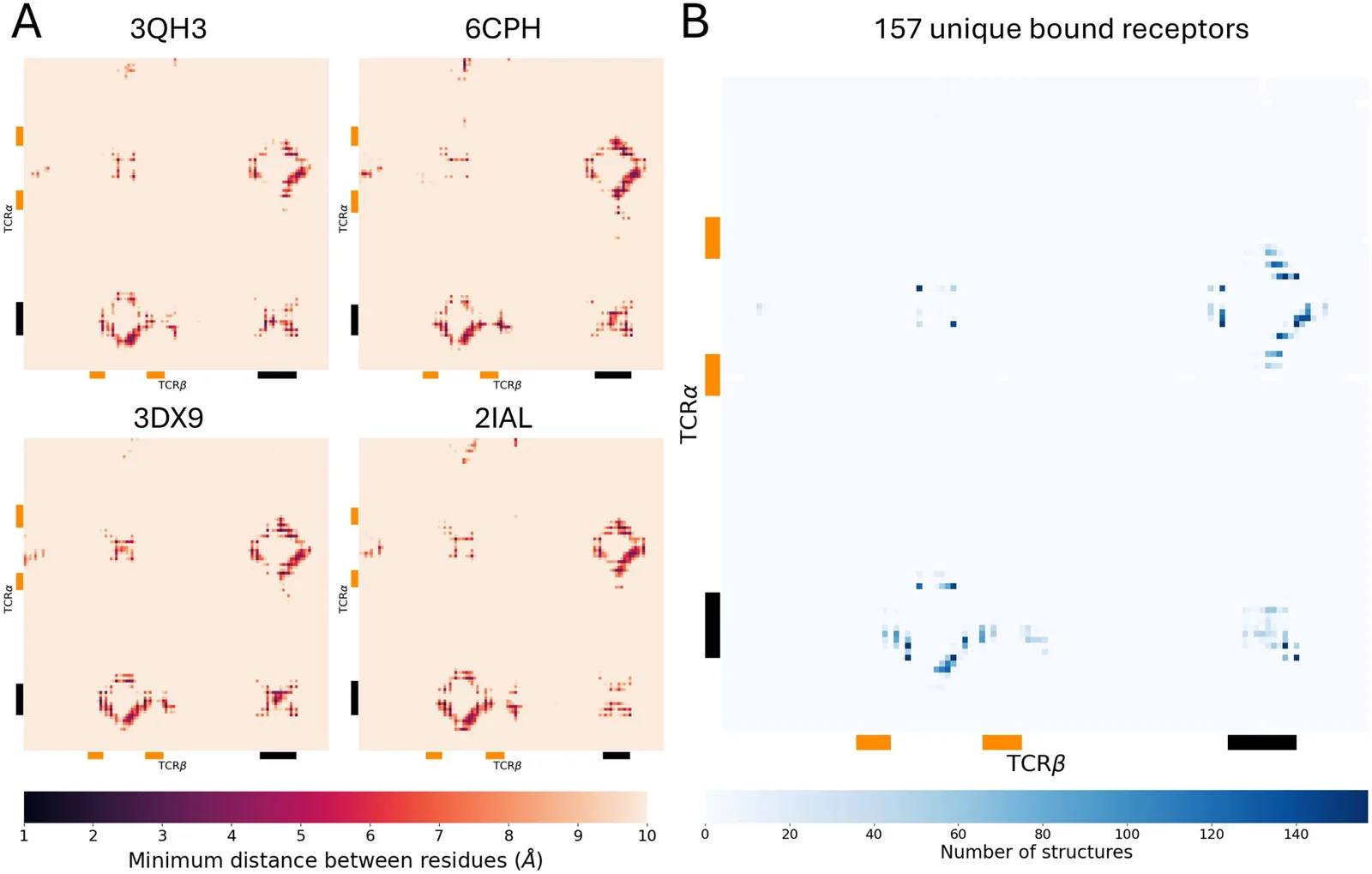
Inter-chain interactions are observed in bound and unbound TCR structures. (A) Inter-chain (TCRα to TCRβ) contact maps for the four structures in Figure 1. The distance (Å, colour bar) is calculated between all pairs of atoms in the two residues, and the minimum distance is shown. (B) Inter-chain contact maps were calculated for 157 unique bound TCRs. A contact is defined as minimum distance between two non-H atoms on two residues <5Å. The heatmap shows the number of structures that are found to make contact at each pair of positions. Only positions present in >75% of structures according to the IMGT numbering scheme are shown. In each heatmap, the positions of CDR1, 2 (orange) and 3 (black) along the sequence are shown as coloured bars along the x and y axes.

Inter-chain distances for the four unligated TCRs in Fig. 1 also show contacts both in the conserved framework regions and in the CDR loops (Fig. 3A). These interactions are also observed in most studied structures (Figs 3B and S7A-C, available as supplementary data at *Bioinformatics* online; and quantified in Fig. S6B-C, available as supplementary data at *Bioinformatics* online). Specifically, framework region 2 (FR2), found between CDR1 and CDR2, makes contacts with FR2 on the other chain, as well as with the edges of the CDR3, where the CDR3 sequence is least variable (the diamond-shaped pattern, median 9 contacts [range 0–17] between FR2α-CDR3β and median 7 contacts [range 1–16] between FR2β-CDR3α, Fig. S6B, available as supplementary data at *Bioinformatics* online; contacts with edges and middle of the CDR3 are quantified in Fig. S6C, available as supplementary data at *Bioinformatics* online: median of 3 [range 0–9] central contacts for FR2α-CDR3β and of 2 [range 0–8] for FR2β-CDR3α compared to median of 6 [range 0–12] edge contacts for FR2α-CDR3β and of 5 [range 0–10] for FR2β-CDR3α). Inter-chain contacts are also observed between CDR1 and 2 and the CDR3 on the paired chain (Fig. S6B, available as supplementary data at *Bioinformatics* online). We observed contacts between the two CDR3s (median of 7 contacts, range 1–22), whilst no contacts are observed between the germline loops CDR1 and CDR2 on the two chains in any of the 340 PDB files analysed (Fig. S6B, available as supplementary data at *Bioinformatics* online). The pattern of contacts between the two CDR3s is X-shaped: the interactions involve the variable junctional regions, but fewer interactions are observed between the central variable regions and the conserved segments on the loop (quantified in Fig. S6C, available as supplementary data at *Bioinformatics* online: median of 3 [range 0–13] interactions with the central segment, compared to median of 2 [range 0–6] for the edges). Notably, these patterns can also be observed in TCRs which bind pMHC with reverse orientation (Fig. S7D, available as supplementary data at *Bioinformatics* online). Overall, these results suggest that the CDR loops form multiple intra- and inter-chain contacts independently of pMHC binding.

### 3.2 Quantifying sequence inter-dependence between TCR*α* and *β* in epitope-specific repertoires using MI

We examined whether, in the context of sets of TCRs recognising a shared pMHC, the sequences of the TCR α and β were determined independently by the requirement to bind the specific antigen, or whether the α and β CDR sequences were dependent on each other. We initially observed increased sequence similarity in TCRβ paired to similar TCRα, and vice versa (Appendix A1, available as supplementary data at *Bioinformatics* online; median increase of 0.56 and 0.15, respectively). We quantified interchain constraints in a more generalisable way using MI to measure the extent to which a change in one chain is accompanied by a complementary change in the paired chain. We estimated the MI between TCRα and β regions of epitope-specific repertoires using a pairwise approximation and corrected for sample size and background distributions. As negative controls, we estimated the MIs for all TCRs in VDJDb (“background”), and for a single sample of sorted CD4+ naïve T cells from Tanno *et al.* (2020) (“Tanno::A1::naïve”). The former contains information about the 22 epitopes under study pooled together and might have some residual epitope-specific signal. The latter depends only on the biology of receptor recombination and thymic selection, without the influence of antigen selection.

**Figure 4:**
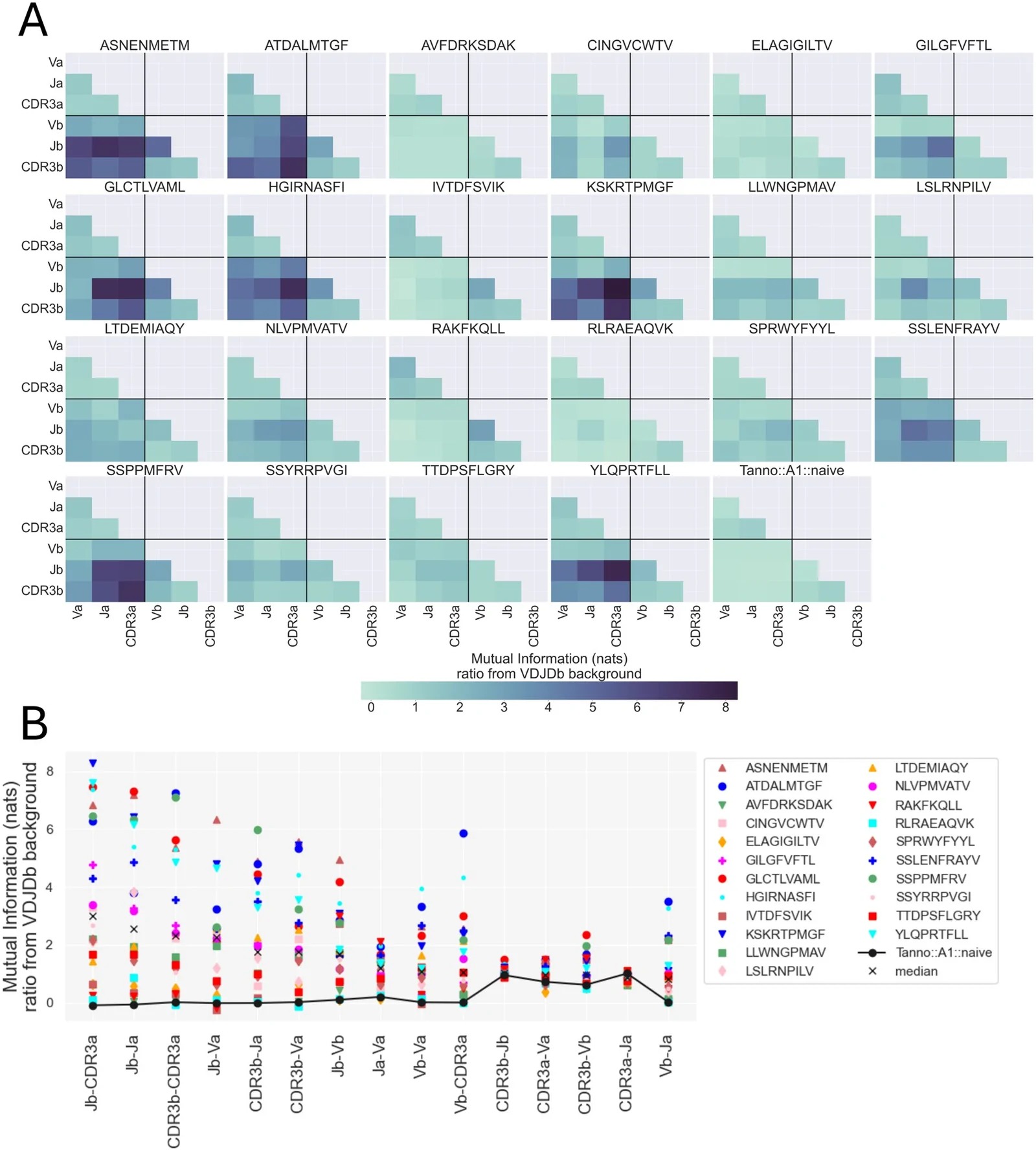
Mutual information between TCR regions in epitope-specific repertoires. (A) Mutual information (MI) between different regions of the TCR (V genes, J genes, CDR3) was calculated for each epitope-specific repertoire. In each heatmap, the top-left and bottom-right quadrants contain intra-chain MI (α in top-left and β in bottom-right). The bottom-left quadrant shows the inter-chain MI. The MI was estimated for various subsamples for each epitope, and corrected for finite size effects (Fig. S5A, available as supplementary data at *Bioinformatics* online). The MI is shown as fold change from VDJDb background to normalise across MI pairs with different alphabet sizes. A pairwise approximation is used in cases involving CDR3 (see Methods). (B) Summary of the plots in A. The *x*-axis is ordered according to the median MI across all epitope repertoires (indicated with an X). Each epitope is identified by a unique combination of colour and shape.

Figure 4A shows the estimated MIs for each epitope compared to VDJdb background. The naïve repertoire showed no increase in MI compared to the pooled VDJDb repertoire. In contrast, most individual epitopes showed an increase in MI compared to the background. A few epitopes, such as AVFDRKSDAK showed low or no signal. The MIs for all epitopes are summarised in Fig. 4B and the MIs for all pairs in all epitopes are in Table S2, available as supplementary data at *Bioinformatics* online.

**Figure 5:**
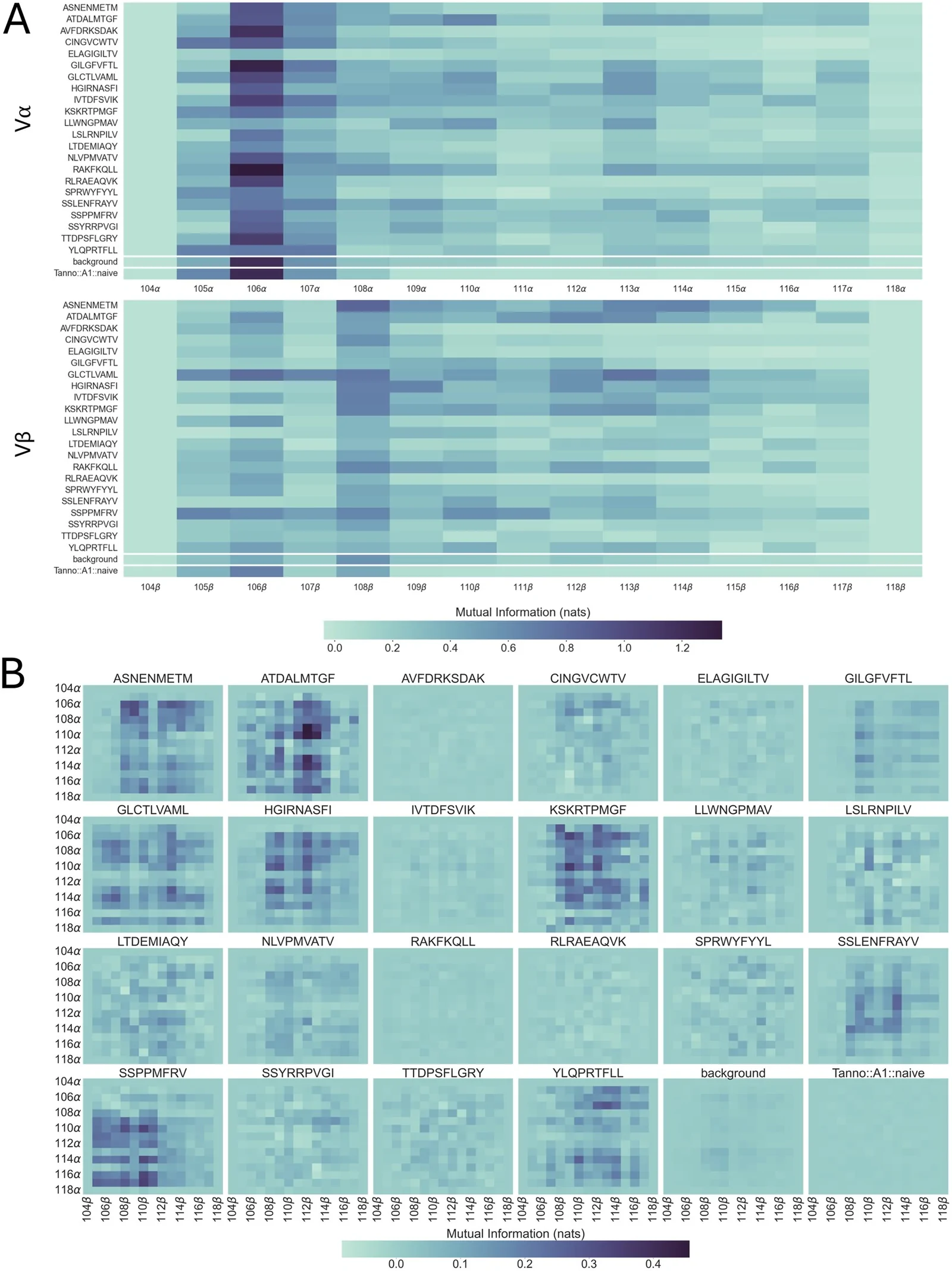
Mutual information between CDR3 and associated V gene and CDR3α-CDR3β. (A) Mutual information (MI) between V genes and each residue position on the CDR3. Top: Vα to CDR3α; bottom: Vβ to CDR3β. (B) MI between each pairs of positions on CDR3α and CDR3β. The MI was calculated with pairwise approximation, extrapolation and correction for background (for both A and B, see Methods). IMGT positions between residues 111 and 112 are not shown as they are not present in most TCRs analysed.

To understand the statistical dependence between V gene and CDR3 in more detail, we examined the estimated intra-chain MI between the V gene and each CDR3 residue (Fig. 5A). As CDR3 positions 104 and 118 on both chains are conserved (C for position 104, F or W most commonly for position 118), the MI is ∼0. The initial residues (105–108) on CDR3α and β show a higher MI with the V gene than the rest of the CDR3 for all epitopes and in the background, since the beginning of the CDR3 is usually encoded by the end of the V gene. However, we also observed MI between the V gene and the central residues of the CDR3 for most epitopes but not in the naïve repertoire. Overall, V-CDR3 MI is higher than background in 10/22 and 9/22 epitopes and higher than naïve repertoire in 18/22 and 19/22 epitopes for Vα-CDR3α and Vβ-CDR3β, respectively.

We also examined the MI between the CDR3α and CDR3β (Fig. 5B). Many epitopes but not background or naïve repertoire showed inter-dependence between α and β CDR3 sequences, with an epitope-specific MI footprint (CDR3α-CDR3β MI is greater than background in 17/22 epitopes). Antigen-dependent TCR selection therefore imposes mutual sequence constraints between the two chains of the TCR. As in previous analyses, epitope AVFDRKSDAK showed very low inter-CDR3 MI.

In order to align the sequences to calculate MI, the CDR3 sequences were padded. We thus investigated the impact of padding on MI. As expected, residue positions where padding occurs more often have lower MI (Fig. S8, available as supplementary data at *Bioinformatics* online, slope <0 and *P*-value <0.05 for all relationships studied). To get a better sense of the impact of padding on MI estimation, we also compared two padding methods: padding in the middle of the sequence (as in IMGT numbering, used in all analyses) and padding at the C terminus of the sequence. MI estimated from CDR3 sequences with either method (Fig. S9, available as supplementary data at *Bioinformatics* online) were correlated for most MI pairs (Spearman ρ>0.95 in 7/9 pairs, CI and *P*-values for each comparison reported in the figure). The correlation between estimated MIs with the two padding strategies for Jα-CDR3α and Jβ-CDR3β was lower (Spearman ρ=0.68, 95% CI = [0.39–0.85], *P*-value = $2.26\times 10^{-4}$ and ρ=0.66, 95% CI = [0.35–0.84], *P*-value = $4.49\times 10^{-4}$, respectively). This reduction is expected since the J gene partially determines the sequence of the C terminal of the CDR3 and padding in the middle will better preserve the alignment at the C terminus, while padding at the end will disrupt it and therefore reduce the MI. Overall, the MI calculation was robust to the padding strategy, which did not alter the relative signal of epitopes and background.

Finally, we extended the MI estimation to poly-epitope repertoires. We downloaded TCR repertoires from the OTS database (Raybould *et al*. 2024) and calculated TCR αβ MI for all studies (Fig. S10, Table S3, available as supplementary data at *Bioinformatics* online). Sorted memory and AIM + cells, which are enriched for antigen-specific groups of TCRs, have higher median CDR3α-CDR3β MI compared to unsorted and unstimulated T cells.

### 3.3 Examining MI variation across epitopes

A striking feature of the TCR chain sequence inter-dependence was the variability observed between epitopes. Understanding the factors which drive this epitope dependence may have important implications for predicting the antigen specificity of an orphan TCR.

**Figure 6:**
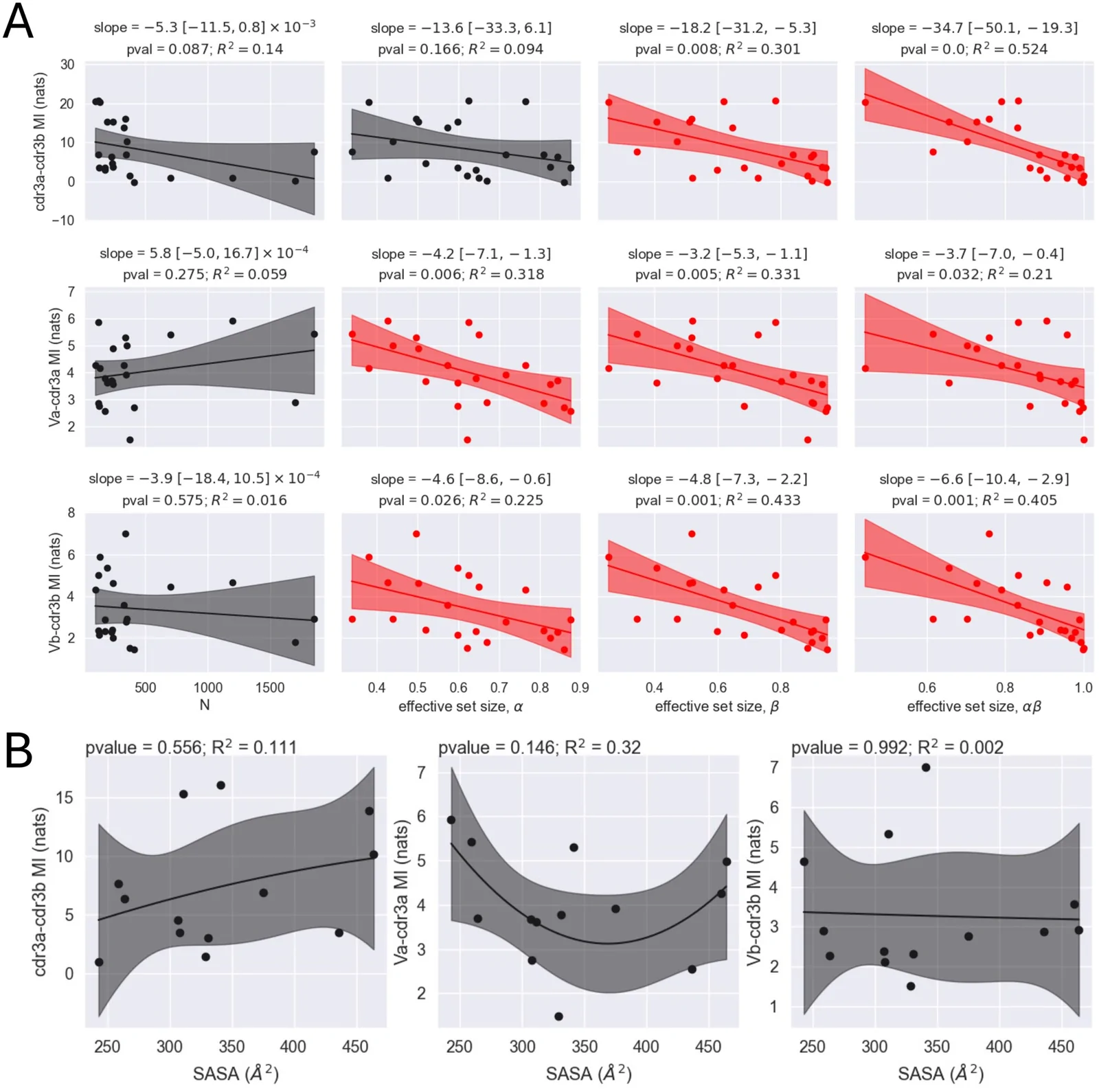
Mutual information correlates to effective set size but not peptide solvent-accessible area. (A) Correlation of mutual information (MI) and effective set size. Effective set size was calculated by grouping all sequences with Hamming distance of <2 on each chain and <3 for the paired chains, normalised by total repertoire size (N). Correlation with N is also shown as a control, and no significant correlation was found in this case. All correlations with effective set size and N are shown in Fig. S12, available as supplementary data at *Bioinformatics* online. (B) Correlation of MI and solvent-accessible surface area (SASA) for the available epitopes (Table 1). Correlations were calculated by fitting a linear regression. The slope of the linear fits and $R^{2}$ of all fits are reported. 95% CIs for the slope are indicated in square brackets. *P*-value for the *F*-test and $R^{2}$ were calculated for each fit. Fits that have *P*-value<0.05 are highlighted in red. The shaded area represents the 95% CI of the fit.

To validate that the observed results were not simply an artefact of finite size effects, we correlated estimated MI and epitope repertoire size (*N*). We did not detect significant correlations for most of the relationships investigated (Figs 6A, left-most column and Fig. S11A, available as supplementary data at *Bioinformatics* online).

Epitope-specific responses are characterised by differing levels of sequence similarity (see for instance Dash *et al.* 2017) and occasionally public TCR chains (see for instance Zhong *et al.* 2007, Pogorelyy *et al*. 2022). To evaluate the impact of similar or public TCR chains in MI estimation, we measured the correlation between MI and the effective set size of CDR3α, CDR3β and CDR3αβ, which gives a measure of the number of distinct sequence clusters in a repertoire (Figs 6A and S11B, available as supplementary data at *Bioinformatics* online). β and αβ effective set sizes were negatively correlated with MI calculated from CDR3α-CDR3β (slope = –18.2, 95% CI = [–31.2, –5.3] and slope = −34.7, 95% CI = [–50.1, –19.3], respectively), Vα-CDR3α (slopes = –3.2 [–5.3, –1.1] and –3.7 [–7.0, –0.4], respectively) and Vβ-CDR3β (slopes = –4.8 [–7.3, –2.2] and –6.6 [–10.4, –2.9], respectively). αβ effective set size was also correlated to the MI from many other evaluated pairs (Fig. S11B, available as supplementary data at *Bioinformatics* online). Effective α set size was negatively correlated with Vα-CDR3α (slope = –4.2 [–7.1, –1.3]) and Vβ-CDR3β (slope = −4.6 [–8.6, –0.6]), but not CDR3α-CDR3β. Overall, these significant negative correlations suggest repertoire characteristics can explain ∼20−67% of the variability in estimated MI across epitopes and that repertoires dominated by a single sequence or cluster of similar sequences (smaller set size) are associated with a higher MI.

We also examined whether the MI depends on how many features the peptide offers for the TCR to bind. We approximated the “featureness” of a peptide by its solvent-accessible surface area (SASA) for the 13 epitopes where a structure was available (Table 1). Epitopes that have a relatively featureless surface (low SASA), or which are bulged out of the MHC (high SASA) tend to generate less diverse TCR repertoires (Turner *et al*. 2005, 2006) and might therefore have higher MI. A parabola fitted to the relationship between SASA and MI did not show any significant relationship (Fig. 6B). However, the small number of epitopes examined limited the statistical power of the analysis to only detect large effect sizes, and moderate to small effects were below the threshold of detection with 80% power and *P*-value <0.05 (Fig. S12, available as supplementary data at *Bioinformatics* online).

### 3.4 Predicting TCRαβ pairing *de novo*

Given the observed sequence signals in TCR pairing, we tested whether TCRαβ pairing could be predicted *de novo*. Sequence-based methods have been very valuable in studying the evolutionary constraints imposed on the sequences of co-evolving protein families (De Juan *et al*. 2013). Epitope-specific TCR pairing signals do not arise from the evolutionary process at play in interacting protein families, but rather from antigen selection of TCRs produced by V(D)J recombination. The methods used to study protein co-evolution, however, are generalisable to studying the constraints imposed by a selection force on any sets of pairs of variable interacting proteins, and hence to predict pairing. We therefore adapted two methods: a MI-based algorithm (MI-IPA, Bitbol 2018), which exploits the MI between co-evolving residue pairs (Fig. 5), and a graph alignment algorithm (GA, Bradde *et al*. 2010), which exploits the similarity in phylogenetic trees, or in our use-case, similarity graphs, Figs A3 and A4, available as supplementary data at *Bioinformatics* online). We also tested a combination of the two algorithms (GA + MI-IPA, Gandarilla-Pérez *et al*. 2023).

**Figure 7:**
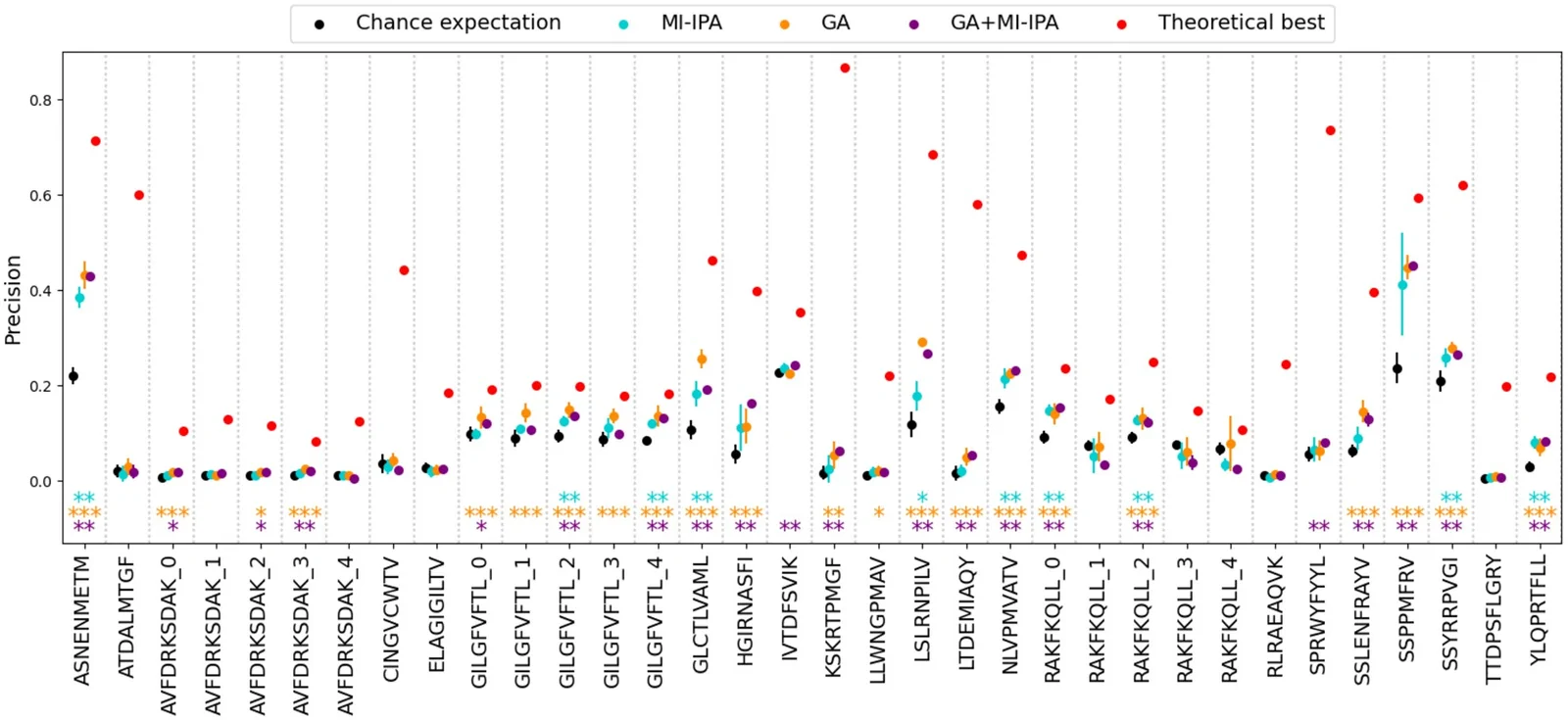
Pairing models perform better than random at pairing TCRαβ in epitope-specific repertoires. Precision is calculated over 10 repeats of the MI-IPA or GA + MI-IPA (cyan and magenta, respectively) or 100 repeats of the GA (orange) for all epitopes. For epitopes with >1000 sequences, 5 subsamples of 700 sequences are shown. The IPA is run both with λ=1 (chance expectation, black) and λ=0.6 (cyan). Error bars correspond to the standard deviation over the repeats. The red circles correspond to the theoretical best performance, i.e. the MI-IPA initialised on all correct pairs and run once to pair all sequences. One-sided Mann-Whitney *U*-test is calculated for each epitope for the MI-IPA, GA or GA + MI-IPA using the alternative hypothesis that the model performs better than chance expectation. Adjusted *P*-values with Bonferroni correction are indicated by asterisks in the corresponding colour: ***<0.001; 0.001≤**<0.01; 0.01≤*<0.05.

In 6/22 epitopes, the MI-IPA performs significantly better than chance expectation (median fold change from chance expectation across all 22 epitopes = 1.29, range = 0.51–2.70; Fig. 7). The GA shows significantly better performance than chance expectation in 14/22 epitopes (median fold change from chance expectation across all 22 epitopes = 1.49, range = 0.81–3.11; we consider it successful for large epitopes based on whether it is significant in at least 3/5 subsamples; Fig. 7). As pairing assignments were repeated 10 times by the MI-IPA and 100 times by the GA for each epitope to account for stochasticity, we explored whether the stability of the assignments between repeats could provide a confidence score for each pair (as in Bitbol *et al*. 2016). For most epitopes, the majority of pairs are not assigned stably across repeats (Figs S13A and S14A, available as supplementary data at *Bioinformatics* online). Notably, stable pairs have a larger proportion of correct pairs in 23/27 epitope sets with stable pairs in the MI-IPA (Fig. S13B and C, available as supplementary data at *Bioinformatics* online) and 22/23 epitope sets with stable pairs in the GA (Fig. S14B and C, available as supplementary data at *Bioinformatics* online). Consistently, epitopes which show better performance show a larger proportion of stable pairs (Figs S13D and S14D, available as supplementary data at *Bioinformatics* online, Spearman ρ=0.796, 95% CI = [0.627, 0.894], *P*-value $=1.8\times 10^{-8}$ for MI-IPA and Spearman ρ=0.699, 95% CI = [0.472, 0.839], *P*-value $=4.3\times 10^{-6}$ for GA).

We then combined the two methods by using stable pairs from the GA assignments as training set for the MI-IPA (Gandarilla-Pérez *et al*. 2023), but allowed the MI-IPA to re-pair sequences in the training set to potentially correct any mistakes in the GA classification (Table S4, available as supplementary data at *Bioinformatics* online). The combination of the two methods occasionally marginally improved on either method alone and performs significantly better than chance expectation in 15/22 epitopes (median fold change from chance expectation across all 22 epitopes = 1.43, range = 0.37–3.59, Fig. 7). However, unlike the MI-IPA and GA, the GA + MI-IPA shows a larger proportion of stable pairs with 12/34 epitope sets having >95% stable pairs (Fig S15A and D, available as supplementary data at *Bioinformatics* online). However, there is a lower enrichment for correct pairs in the stable pairs (Fig. S15B and C, available as supplementary data at *Bioinformatics* online: 8/34 epitopes have the same or lower percentage of correct pairs among the stable pairs compared to the overall percentage of correct pairs, instead of 4/27 and 1/23 for MI-IPA and GA, respectively, Figs S13C and S14C, available as supplementary data at *Bioinformatics* online). We attribute this to the use of a training set in the MI-IPA, as the trajectory of the MI-IPA is less random when a training set is provided.

The limitation in model prediction accuracy can arise in the method (i.e. the way the algorithm selects pairs does not find the optimal solution) or in the data (i.e. the signal in the data is too low to achieve pairing). To distinguish these two limiting factors, we calculated the theoretical best performance for the MI-IPA. We initialised the model with all correctly-paired αβ for each epitope and ran a single iteration of the MI-IPA to pair all available α/β from that epitope repertoire again, effectively teaching the model the pairing rules *a priori* and over-fitting (Fig. 7). In all epitopes, the pairing algorithms under-perform compared to the theoretical limit but the theoretical limit remains well below 1 for all epitope repertoires, suggesting that both method and data limitations are present.

Finally, we tested whether TCR sequence embedding methods or models trained to predict TCR-epitope binding could capture the pairing signal. Chain pairing was predicted within epitope-specific repertoires using TULIP (Meynard-Piganeau *et al.* 2024), a method developed for binding prediction, and SCEPTR (Nagano *et al*. 2025), a method for TCR sequence embedding. We found that pairing models consistently perform better than both TULIP and SCEPTR for TCR αβ pairing (Fig. S16, available as supplementary data at *Bioinformatics* online), suggesting that interchain MI is often overlooked in other specificity-prediction methods. However, both TULIP and SCEPTR seem to be able to learn some of the pairing signal in their training, as both perform marginally better than chance expectation for a few epitopes.

### 3.5 Correlates of pairing performance and repertoire characteristics

**Figure 8:**
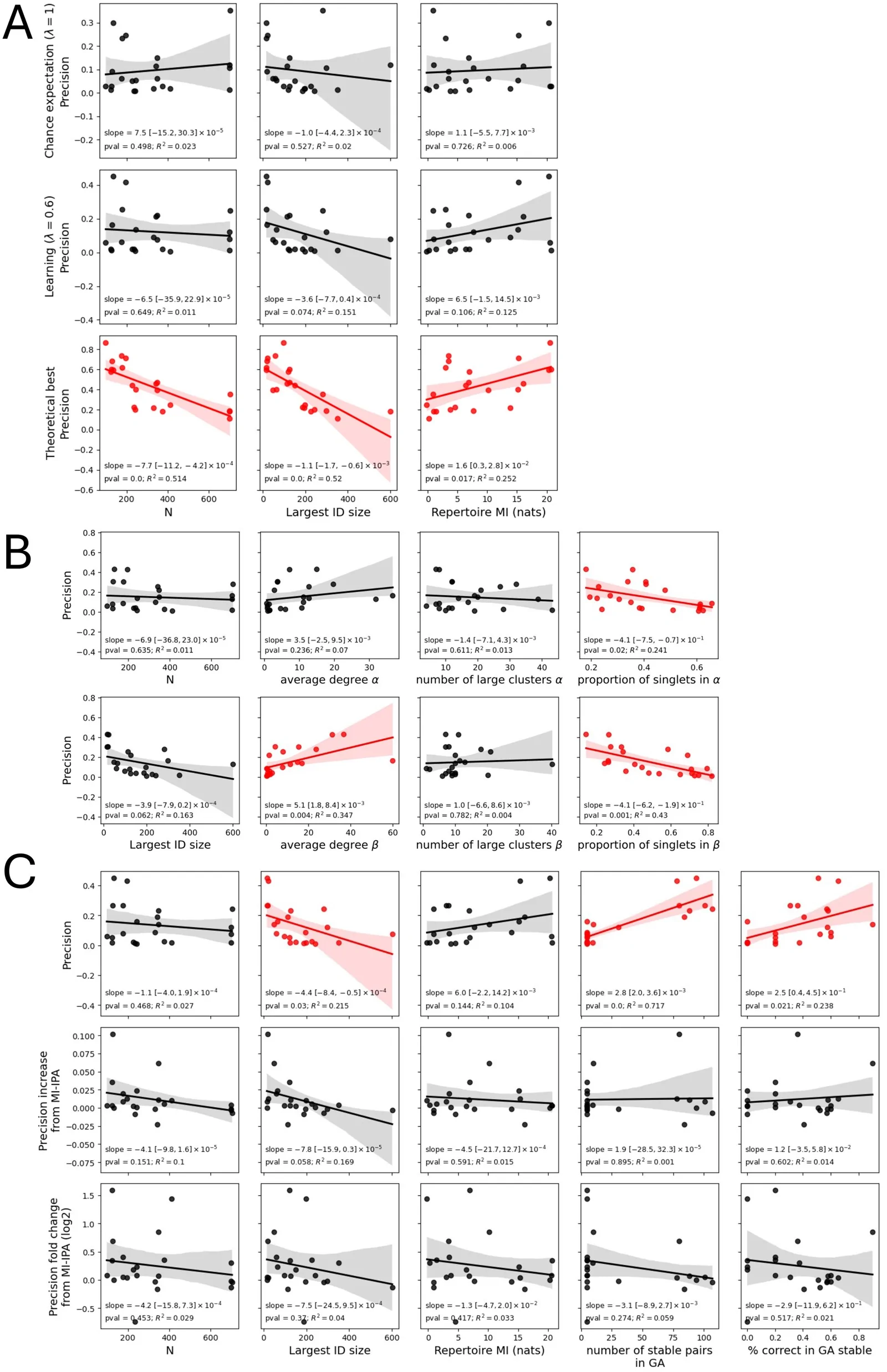
Correlation of model performance and repertoire characteristics. Correlation between model pairing prediction performance, repertoire size and MI. (A) A linear regression was calculated to evaluate the effect of repertoire size (N), largest individual repertoire size (Largest ID size) and total mutual information (Repertoire MI, calculated between CDR3α and CDR3β as in Fig. 4, without fold change from background) on the precision for MI-IPA (no learning: [confidence = none; λ=1.0; θ=0.6]; learning: [confidence = none; λ=0.6; θ=0.6]; theoretical best: [confidence = none; λ=0.6; θ=0.6; correct pairs as training set]). Subsamples from large repertoires are included as an average, and the repertoire MI is calculated on the complete sample. (B) A linear regression was calculated to evaluate the effect of N, Largest ID size and similarity network characteristics on the precision for GA. The graph characteristics measured are: average degree of nodes, number of clusters of size ≥3 and proportion of sequences that do not have any neighbours (singlets). These are calculated on the graph built by drawing edges between sequences at Levenshtein distance <3 on CDR3α and CDR3β separately. Subsamples from large repertoires are included as an average, and the graph properties are calculated on each subsample separately. (C) A linear regression was calculated to evaluate the effect of N, Largest ID size, Repertoire MI, number of stable GA pairs and proportion of the GA training set that is correct (% correct in GA stable) on the precision for GA + MI-IPA. Subsamples from large repertoires are included as an average, the repertoire MI is calculated on the complete sample and the GA stability results are calculated for each subsample separately and averaged. In each panel, the precision is calculated using the modal β for each α over multiple iterations of the model. Since multiple CDR3β may be selected the same number of times for one CDR3α, one is selected at random. To mitigate for variation, the mode selection is run 100 times and the average precision is used. In each panel, the slope of the linear fits, the $R^{2}$ and the *P*-value for the regression are shown. 95% CIs for the slope are indicated in square brackets. The scatterplots are red when *P*-value for the *F*-statistics of the regression is <0.05. The solid line shows the calculated regression and the shaded area the 95% confidence interval of the prediction.

The pairing prediction performance varied considerably between epitopes (Fig. 7). For each model, we identified factors that could impact performance and correlated them with the observed pairing performance. For all models, we evaluated the total size of the repertoire and the size of the individual repertoires, as they increase the combinatorial number of pairs to be evaluated (Bitbol 2018). For the MI-IPA, the predictive power may also depend on the MI available in each epitope-specific repertoire (Fig. 4), whilst the GA might be sensitive to the topology of the similarity networks generated from each of these epitopes. The theoretical maximal, but not the actual MI-IPA performance (calculated on the consensus assignment over 10 repeats) was inversely correlated to individual repertoire size (slope of the linear fit = $-1.1\times 10^{-3}$, 95% $\text{CI}=[-1.7\times {10^{-}}^{3},-0.6\times {10^{-}}^{3}]$) and total epitope repertoire size (slope = $-7.7\times 10^{-4}$, 95% $\text{CI}=[-11.2\times {10^{-}}^{4},-4.2\times {10^{-}}^{4}]$), and positively correlated to MI (slope = $1.6\times 10^{-2}$, 95% $\text{CI}=[0.3\times {10^{-}}^{2},2.8\times {10^{-}}^{2}]$; Fig. 8A). These 3 variables together can explain 59.1% of the variance observed across epitopes in the theoretical best performance, and 34.7% in the learning scenario (Table S5, available as supplementary data at *Bioinformatics* online). GA performance (calculated on the consensus assignment over 100 repeats) correlated with the extent of sequence clustering (higher average degree on the TCRβ similarity graph [slope of the linear fit = $5.1\times 10^{-3}$, 95% $\text{CI}=[1.8\times {10^{-}}^{3},8.4\times {10^{-}}^{3}]$], smaller proportion of singlets calculated on the graph generated using pairwise Levenshtein distance with distance threshold <3 [slope for both α and β = $-4.1\times 10^{-1}$, 95% CI $\alpha =[-7.5\times {10^{-}}^{1},-0.7\times {10^{-}}^{1}]$ and 95% CI $\beta =[-6.2\times {10^{-}}^{1},-1.9\times {10^{-}}^{1}]$]; Fig. 8B). The model built with these variables can explain 79.9% of the variance between epitopes (Table S6, available as supplementary data at *Bioinformatics* online).

Finally, we looked at factors that impact the performance of the GA + MI-IPA, as well as the increase and fold-change in precision of the GA + MI-IPA compared to the MI-IPA alone. On top of the repertoire characteristics, we extracted the number of stable pairs from the GA and the percentage of the GA stable set that is correct. The number of stable pairs in the GA and percentage of them that are correct are positively correlated with precision of the GA + MI-IPA (slope $=2.8\times {10^{-}}^{3}$, 95% $\text{CI}=[2.0\times {10^{-}}^{3},3.6\times {10^{-}}^{3}]$ and slope $=2.5\times {10^{-}}^{1}$, 95% CI $=[0.4\times {10^{-}}^{1},4.5\times {10^{-}}^{1}]$, respectively), and precision is negatively correlated to the largest ID size (slope $=-4.4\times {10^{-}}^{4}$, 95% CI $=[-8.4\times {10^{-}}^{4},-0.5\times {10^{-}}^{4}]$). No factor explained why certain epitopes showed improvement using the combined models and others did not. Together, these variables explain 81.8% of the observed variance in precision of the GA + MI-IPA, but explain less < 50% of the variance in improvement compared to MI-IPA alone (Table S7, available as supplementary data at *Bioinformatics* online).

## 4 Discussion

In contrast to the wealth of studies that examine the interactions between TCR CDRs and pMHC, very few have examined the interactions between the α and β chain CDRs. In this study we address two related but distinct aims. The first aim is to comprehensively document the amino acid contacts observed between TCR CDR loops in published TCR crystallographic structures. We observed a conserved pattern of intra-chain interactions between CDR1 and both CDR2 and CDR3 loops in both chains, and more limited contacts between CDR2 and CDR3 (Figs 1, 2 and S6A, available as supplementary data at *Bioinformatics* online). We also quantified inter-chain interactions between the conserved FR2 regions on each chain, and at the interface between the two CDR3s (Figs 3 and S6B-C, available as supplementary data at *Bioinformatics* online). These contacts are observed in both pMHC-bound and free TCR structures, although the exact topology of the contact can change upon binding. Contacts between CDR loops are therefore an important intrinsic feature of the TCR which are likely to affect overall receptor shape, antigen binding and stability. Further structural and functional studies will be required to quantify the exact impact of these contacts on TCR-antigen binding. In contrast, conserved interactions between CDR1 and/or CDR2 on one chain, and CDR1 and/or CDR2 on the other chain were not observed, consistent with the absence of constraints in TRAV/TRBV pairing in the overall repertoire (Dupic *et al*. 2019, Shcherbinin *et al.* 2020).

The observed close packing of the CDR regions of the two chains in the folded TCR structure discussed above suggested the hypothesis that the contribution of each chain to pMHC binding is not independent and consequently that the sequences of the two chains in sets of TCRs with shared pMHC specificity might be interdependent. Indeed, CDR3α and CDR3β sequences within sets of epitope-specific TCRs were shown to consistently share MI (Figs 4 and 5). These observations of chain sequence interdependence are reminiscent of the ‘light chain coherence’ described for antibodies (Jaffe *et al*. 2022). Although Shcherbinin *et al.* (2020) did not find evidence for restriction of TCR chain pairing within epitope-specific repertoires, their study excluded CDR3 sequences that provide the majority of the information content determining epitope binding specificity (Henderson *et al.* 2024).

Mutual sequence interdependence between two sets of proteins allows, at least in principle, prediction of pairing between members of the two sets. A number of sequence-based approaches have already been developed to predict pairing between co-evolving interacting protein families (Korber *et al*. 1993, Fryxell 1996, Dunn *et al*. 2008, De Juan *et al*. 2013, Ochoa and Pazos 2014). Although TCRs do not evolve, but are rather produced independently by V(D)J recombination and then selected by antigen, the mutual interdependence of TCR αβ sequences suggested that similar methods could be applied to predict pairing between the αβ sequences of sets of pMHC-specific TCRs. Prediction of pairing would be of significant value in analysis of TCR repertoires, since bulk TCR sequencing usually produces lists of unpaired TCR α and β sequences. In order to apply the available methods, multiple TCRα and TCRβ chains which bind to the same epitope are treated analogously to ‘paralogs’ from interacting protein families, while similar TCRα or β sequences that bind the same epitope but are found in different individuals are ‘orthologs’. Three co-evolution based methods (Bradde *et al*. 2010, Bitbol 2018, Gandarilla-Pérez *et al*. 2023) were adapted to predict TCRαβ pairing in lists of TCRα and TCRβ sequences annotated to a single epitope. Each model was evaluated for its success in making *de novo* pairing predictions, in the absence of any training data. A significant improvement in pairing performance over random assignment was detected in 16/22 tested epitopes using at least one of the three methods (Fig. 7).

In general, these methods show better performance in the pairing task than existing models for epitope prediction (Meynard-Piganeau *et al.* 2024) or TCR sequence embedding (Nagano *et al*. 2025), suggesting that they are learning an alternative signal for prediction. However, pairing performance remains poor, limiting practical applications for TCR pairing. The addition of more pairs for each epitope is likely to increase performance, as information-based methods are data-thirsty (Bitbol 2018). The current algorithms are also computationally intensive, limiting their application to large epitope-specific TCR sets. Faster methods have been developed (see for instance, Lupo *et al.* 2024) and can be tested on larger datasets in the future. TCR pairing may also be improved by including additional information such as sequence abundance, as α and β chains from the same TCR will vary across different samples in a correlated manner. Finally, the precision calculation assumes all unobserved pairs in the data to be non-binders, but this assumption may be incorrect.

Epitopes differed widely in the amount of MI shared between α and β CDR sequences and in the performance achieved by the pairing algorithms (Figs 6 and 8). This variation may arise from intrinsic features of the pMHCs, and features of the TCR repertoire associated with each pMHC. The feature-ness of a peptide (how much of the peptide extends outside the MHC binding groove), for example, has been shown to impact the diversity of the associated TCR repertoire (Turner *et al*. 2005, 2006). However, no correlation was detected between feature-ness and MI in this study (Fig. 6). In contrast, we found that pMHCs with smaller effective set size of associated TCRs have higher inter-chain MI and better pairing prediction (Fig. 8). The presence of fewer, larger clusters of similar TCR sequences in these sets may allow better prediction of αβ pairing.

The study has a number of limitations. The total number of crystallographic structures is still only a tiny fraction of the number of possible TCRs. Similarly, the number of pMHC annotated TCRs in the VDJDb database (Bagaev *et al*. 2020, Goncharov *et al*. 2022) is only a tiny fraction of possible TCR/pMHC pairings. Furthermore, whilst the analysis was not deliberately limited to specific MHC genes, the VDJDb dataset is known to be skewed and only a small fraction of all MHC alleles and peptide antigens are represented (Hudson *et al*. 2023). Moreover, the MHC information as reported by VDJDb occasionally lacks allele variant information. As detailed above, the number of pMHC/TCR sets was further limited because of the inclusion criteria used for the analyses (more than 100 but less than 10000 TCRs associated with an epitope) to limit the bias in MI calculations for very small TCR sets, and the computational limits in analysis of very large datasets. These criteria restricted the analysed HLA alleles to few alleles from HLA-A and HLA-B and mouse MHCs (Table S1, available as supplementary data at *Bioinformatics* online). While we expect this process to be relevant also for CD4 T cell binding to class II MHC, as exemplified by the signal in CD4 + AIM + populations from OTS (Table S3, available as supplementary data at *Bioinformatics* online), the epitope-specific data from VDJdb has only allowed us to test our hypothesis on CD8 T cells. VDJDb is also known to include erroneously-annotated sequences, which will add noise to the MI calculation (Messemaker *et al*. 2025). The estimation of inter-sequence MI is also subject to some technical limitations, such as finite size effects, padding artefacts, heterogeneity in sequencing platforms and other batch effects between experiments. Although every effort was made to control for these factors, some residual effect cannot be excluded. Finally, MI may not capture higher-order interactions involving epistatic interactions between multiple amino acid positions.

In conclusion, our study documents extensive conserved interactions between the CDR sequences which form the binding surface of a TCR. Inter-chain contacts can stabilise the conformational flexibility of the loops, reducing the entropy of the free TCR, and increasing affinity and specificity at the expense of reduced cross-reactivity. In addition, we quantified broader sequence constraints on TCRαβ pairing in the context of antigen-specific TCR sets. These constraints may be imposed directly by the interchain contacts, or through indirect structural constraints imposed on chain interactions in the context of binding to a pMHC. Our study provides a bioinformatic foundation on which to develop functional and structural studies investigating the role of inter-CDR interactions in determining the kinetics and thermodynamics of TCR-antigen binding. Tuning inter-CDR interactions may represent an under-appreciated mechanism to optimise the design of TCRs and TCR analogues for therapeutic purposes, and may also have broader implications for predicting TCR/pMHC binding.

## Supplementary Material

btag551_Supplementary_Data

## Data Availability

No new data was generated with this paper. The data analysed can be downloaded from VDJDb (https://vdjdb.cdr3.net/), the Structural T Cell Receptor Database (STCRDab, https://opig.stats.ox.ac.uk/webapps/stcrdab-stcrpred/) and the Observed TCR Space database (OTS, https://opig.stats.ox.ac.uk/webapps/ots/). Processed data from Tanno *et al.* (2020) is available from: https://zenodo.org/records/14288119. The code needed to reproduce the analysis in this manuscript is available at https://github.com/mm523/TCRab-pairing and is deposited on Zenodo https://doi.org/10.5281/zenodo.21390616.

## References

[btag551-B1] Archbold JK , Macdonald WA, Gras S et al Natural micropolymorphism in human leukocyte antigens provides a basis for genetic control of antigen recognition. J Exp Med 2009;206:209–19. 10.1084/JEM.20082136. 19139173 PMC2626662

[btag551-B2] Bagaev DV , Vroomans RM, Samir J et al VDJdb in 2019: database extension, new analysis infrastructure and a T-cell receptor motif compendium. Nucleic Acids Res 2020;48:D1057–62. 10.1093/nar/gkz874. 31588507 PMC6943061

[btag551-B3] Baulu E , Gardet C, Chuvin N et al TCR-engineered T cell therapy in solid tumors: state of the art and perspectives. Sci Adv 2023;9:eadf3700. 10.1126/sciadv.adf3700. 36791198 PMC9931212

[btag551-B4] Bitbol A-F. Inferring interaction partners from protein sequences using mutual information. PLoS Comput Biol 2018;14:e1006401. 10.1371/journal.pcbi.1006401. 30422978 PMC6258550

[btag551-B5] Bitbol A-F , Dwyer RS, Colwell LJ et al Inferring interaction partners from protein sequences. Proc Natl Acad Sci USA 2016;113:12180–5. 10.1073/pnas.1606762113. 27663738 PMC5087060

[btag551-B6] Bovay A , Zoete V, Dolton G et al T cell receptor alpha variable 12-2 bias in the immunodominant response to yellow fever virus. Eur J Immunol 2018;48:258–72. 10.1002/eji.201747082. 28975614 PMC5887915

[btag551-B7] Bradde S , Braunstein A, Mahmoudi H et al Aligning graphs and finding substructures by a cavity approach. EPL (Europhysics Letters) 2010;89:37009. 10.1209/0295-5075/89/37009 http://arxiv.org/abs/0905.1893.

[btag551-B8] Campillo-Davo D , Flumens D, Lion E. The quest for the best: how TCR affinity, avidity, and functional avidity affect TCR-engineered T-cell antitumor responses. Cells 2020;9:1720. 10.3390/cells9071720. 32708366 PMC7408146

[btag551-B9] Carter JA , Preall JB, Grigaityte K et al Single T cell sequencing demonstrates the functional role of α β TCR pairing in cell lineage and antigen specificity. Front Immunol 2019;10:1516. 10.3389/fimmu.2019.01516. 31417541 PMC6684766

[btag551-B10] Chaurasia P , Nguyen TH, Rowntree LC et al Structural basis of biased T cell receptor recognition of an immunodominant HLA-A2 epitope of the SARS-CoV-2 spike protein. J Biol Chem 2021;297:101065. 10.1016/j.jbc.2021.101065. 34384783 PMC8352664

[btag551-B11] Dash P , Fiore-Gartland AJ, Hertz T et al Quantifiable predictive features define epitope-specific T cell receptor repertoires. Nature 2017;547:89–93. 10.1038/nature22383. 28636592 PMC5616171

[btag551-B12] Davis MM , Bjorkman PJ. T-cell antigen receptor genes and T-cell recognition. Nature 1988;334:395–402. 10.1038/334395a0. 3043226

[btag551-B13] De Juan D , Pazos F, Valencia A. Emerging methods in protein co-evolution. Nat Rev Genet 2013;14:249–61. 10.1038/nrg3414. 23458856

[btag551-B14] Deng L , Langley RJ, Brown PH et al Structural basis for the recognition of mutant self by a tumor-specific, MHC class II–restricted T cell receptor. Nat Immunol 2007;8:398–408. 10.1038/ni1447. 17334368

[btag551-B15] Dunbar J , Deane CM. ANARCI: antigen receptor numbering and receptor classification. Bioinformatics 2016;32:298–300. 10.1093/bioinformatics/btv552. 26424857 PMC4708101

[btag551-B16] Dunn S , Wahl L, Gloor G. Mutual information without the influence of phylogeny or entropy dramatically improves residue contact prediction. Bioinformatics 2008;24:333–40. 10.1093/bioinformatics/btm604. 18057019

[btag551-B17] Dupic T , Marcou Q, Walczak AM et al Genesis of the α β T-cell receptor. PLoS Comput Biol 2019;15:e1006874. 10.1371/journal.pcbi.1006874. 30830899 PMC6417744

[btag551-B18] Fryxell KJ. The coevolution of gene family trees. Trends Genet 1996;12:364–9. 10.1016/S0168-9525(96)80020-5. 8855667

[btag551-B19] Galperin M , Farenc C, Mukhopadhyay M et al CD4+ T cell–mediated HLA class II cross-restriction in HIV controllers. Sci Immunol 2018;3:687. 10.1126/SCIIMMUNOL.AAT0687. 29884618

[btag551-B20] Gandarilla-Pérez CA , Pinilla S, Bitbol A-F et al Combining phylogeny and coevolution improves the inference of interaction partners among paralogous proteins. PLoS Comput Biol 2023;19:e1011010. 10.1371/journal.pcbi.1011010. 36996234 PMC10089317

[btag551-B21] Garboczi DN , Ghosh P, Utz U et al Structure of the complex between human T-cell receptor, viral peptide and HLA-A2. Nature 1996;384:134–41. 10.1038/384134a0. 8906788

[btag551-B22] Glanville J , Huang H, Nau A et al Identifying specificity groups in the T cell receptor repertoire. Nature 2017;547:94–8. 10.1038/nature22976. 28636589 PMC5794212

[btag551-B23] Goncharov M , Bagaev D, Shcherbinin D et al VDJdb in the pandemic era: a compendium of T cell receptors specific for SARS-CoV-2. Nat Methods 2022;19:1017–9. 10.1038/s41592-022-01578-0. 35970936

[btag551-B24] Gras S , Saulquin X, Reiser J-B et al Structural bases for the affinity-driven selection of a public TCR against a dominant human cytomegalovirus epitope. J Immunol 2009;183:430–7. 10.4049/jimmunol.0900556. 19542454

[btag551-B25] Hamelryck T , Manderick B. PDB file parser and structure class implemented in python. Bioinformatics 2003;19:2308–10. 10.1093/bioinformatics/btg299. 14630660

[btag551-B26] Heather JM , Spindler MJ, Alonso MH et al Stitchr: stitching coding TCR nucleotide sequences from V/J/CDR3 information. Nucleic Acids Res 2022;50:e68. 10.1093/nar/gkac190. 35325179 PMC9262623

[btag551-B27] Henderson J , Nagano Y, Milighetti M et al Limits on inferring T cell specificity from partial information. Proc Natl Acad Sci 2024;121(42):e2408696121. 10.1073/pnas.2408696121. 39374400 PMC11494314

[btag551-B28] Hudson D , Fernandes RA, Basham M et al Can we predict T cell specificity with digital biology and machine learning? Nat Rev Immunol 2023;23:511–21. 10.1038/s41577-023-00835-3. 36755161 PMC9908307

[btag551-B29] Ishizuka J , Stewart-Jones GB, van der Merwe A et al The structural dynamics and energetics of an immunodominant T cell receptor are programmed by its Vβ domain. Immunity 2008;28:171–82. 10.1016/j.immuni.2007.12.018. 18275829

[btag551-B30] Jaffe DB , Shahi P, Adams BA et al Functional antibodies exhibit light chain coherence. Nature 2022;611:352–7. 10.1038/s41586-022-05371-z. 36289331 PMC9607724

[btag551-B31] Kesmir C , Borghans JA, de Boer RJ. Diversity of human α β T cell receptors. Science (1979) 2000;288:1135. 10.1126/science.288.5469.1135a.

[btag551-B32] Korber BT , Farber RM, Wolpert DH et al Covariation of mutations in the V3 loop of human immunodeficiency virus type 1 envelope protein: an information theoretic analysis. Proc Natl Acad Sci USA 1993;90:7176–80. 10.1073/pnas.90.15.7176 8346232 PMC47099

[btag551-B33] La Gruta NL , Thomas PG, Webb AI et al Epitope-specific TCRbeta repertoire diversity imparts no functional advantage on the CD8+ T cell response to cognate viral peptides. Proc Natl Acad Sci USA 2008;105:2034–9. 10.1073/pnas.0711682102. 18238896 PMC2538877

[btag551-B34] Leem J , de Oliveira SHP, Krawczyk K et al STCRDab: the structural T-cell receptor database. Nucleic Acids Res 2018;46:D406–12. 10.1093/nar/gkx971. 29087479 PMC5753249

[btag551-B35] Lefranc M-P , Pommié C, Ruiz M et al IMGT unique numbering for immunoglobulin and T cell receptor variable domains and Ig superfamily V-like domains. Dev Comp Immunol 2003;27:55–77. 10.1016/S0145-305X(02)00039-. 12477501

[btag551-B36] Lineburg KE , Grant EJ, Swaminathan S et al CD8+ T cells specific for an immunodominant SARS-CoV-2 nucleocapsid epitope cross-react with selective seasonal coronaviruses. Immunity 2021;54:1055–65.e5. 10.1016/j.immuni.2021.04.006. 33945786 PMC8043652

[btag551-B37] Lupo U , Sgarbossa D, Milighetti M et al DiffPaSS–high-performance differentiable pairing of protein sequences using soft scores. Bioinformatics 2024;41:btae738. 10.1093/bioinformatics/btae738. 39672677 PMC11676329

[btag551-B38] Mayer A , Callan CG. Measures of epitope binding degeneracy from T cell receptor repertoires. Proc Natl Acad Sci 2023;120:e2213264120. 10.1073/pnas.2213264120. 36649423 PMC9942805

[btag551-B39] McBeth C , Seamons A, Pizarro JC et al A new twist in TCR diversity revealed by a forbidden alphabeta TCR. J Mol Biol 2008;375:1306–19. 10.1016/j.jmb.2007.11.020. 18155234 PMC2330282

[btag551-B40] Meijers R , Lai C-C, Yang Y et al Crystal structures of murine MHC class I H-2 Db and Kb molecules in complex with CTL epitopes from influenza a virus: implications for TCR repertoire selection and immunodominance. J Mol Biol 2005;345:1099–110. 10.1016/j.jmb.2004.11.023. 15644207

[btag551-B41] Messemaker M , Kwee BPY, Moravec V et al A functionally validated TCR-pMHC database for TCR specificity model development. 2025. https://www.biorxiv.org/content/10.1101/2025.04.28.651095v3.

[btag551-B42] Meynard-Piganeau B , Feinauer C, Weigt M et al TULIP: a transformer-based unsupervised language model for interacting peptides and T cell receptors that generalizes to unseen epitopes. Proc Natl Acad Sci U S A 2024;121:e2316401121. 10.1073/pnas.2316401121. 38838016 PMC11181096

[btag551-B43] Milighetti M. Analysis of T cell receptor sequence and structure to understand the drivers of antigen specificity. PhD Thesis, UCL (University College London), 2023.

[btag551-B44] Milighetti M , Shawe-Taylor J, Chain B. Predicting T cell receptor antigen specificity from structural features derived from homology models of receptor-peptide-major histocompatibility complexes. Front Physiol 2021;12:730908. 10.3389/fphys.2021.730908. 34566692 PMC8456106

[btag551-B45] Mora T , Walczak AM. How many different clonotypes do immune repertoires contain? Curr Opin Syst Biol 2019;18:104–10. 10.1016/J.COISB.2019.10.001.

[btag551-B46] Nagano Y , Chain B. Tidytcells: standardizer for TR/MH nomenclature. Front Immunol 2023;14:1276106. 37954585 10.3389/fimmu.2023.1276106 PMC10634431

[btag551-B47] Nagano Y , Pyo AGT, Milighetti M et al Contrastive learning of T cell receptor representations. Cell Syst 2025;16:101165. 10.1016/j.cels.2024.12.006. 39778580

[btag551-B48] Ochoa D , Pazos F. Practical aspects of protein co-evolution. Front Cell Dev Biol 2014;2:14. 25364721 10.3389/fcell.2014.00014 PMC4207036

[btag551-B49] Pedregosa F , Varoquaux G, Gramfort A et al Scikit-learn: machine learning in python. J Mach Learn Res 2012;12:2825–30.

[btag551-B50] Pogorelyy MV , Rosati E, Minervina AA et al Resolving SARS-CoV-2 CD4+ T cell specificity via reverse epitope discovery. Cell Rep Med 2022;3:100697. 10.1016/j.xcrm.2022.100697. 35841887 PMC9247234

[btag551-B51] Raybould MIJ , Greenshields-Watson A, Agarwal P et al The observed T cell receptor space database enables paired-chain repertoire mining, coherence analysis, and language modeling. Cell Rep 2024;43:114704. 10.1016/j.celrep.2024.114704. 39216000

[btag551-B52] Reiser J-B , Legoux F, Gras S et al Analysis of relationships between peptide/MHC structural features and naive T cell frequency in humans. J Immunol 2014;193:5816–26. 10.4049/JIMMUNOL.1303084. 25392532

[btag551-B53] Robinson RA , McMurran C, McCully ML et al Engineering soluble T-cell receptors for therapy. Febs J 2021;288:6159–73. 10.1111/febs.15780. 33624424 PMC8596704

[btag551-B54] Schrödinger, LLC. The PyMOL Molecular Graphics System. Version 2.5. 2015.

[btag551-B55] Schwartz GW , Hershberg U. Conserved variation: identifying patterns of stability and variability in BCR and TCR v genes with different diversity and richness metrics. Phys Biol 2013;10:035005. 10.1088/1478-3975/10/3/035005. 23735612

[btag551-B56] Scott DR , Borbulevych OY, Piepenbrink KH et al Disparate degrees of hypervariable loop flexibility control T-cell receptor cross-reactivity, specificity, and binding mechanism. J Mol Biol 2011;414:385–400. 10.1016/J.JMB.2011.10.006. 22019736 PMC3230710

[btag551-B57] Sewell AK. Why must T cells be cross-reactive? Nat Rev Immunol 2012;12:669–77. 10.1038/nri3279. 22918468 PMC7097784

[btag551-B58] Shcherbinin DS , Belousov VA, Shugay M. Comprehensive analysis of structural and sequencing data reveals almost unconstrained chain pairing in TCRα β complex. PLoS Comput Biol 2020;16:e1007714. 10.1371/journal.pcbi.1007714. 32163410 PMC7093030

[btag551-B2903003] Shimoyama Y. (2022). pyCirclize: Circular visualization in Python [Computer software]. https://github.com/moshi4/pyCirclize.

[btag551-B59] Sliz P , Michielin O, Cerottini J-C et al Crystal structures of two closely related but antigenically distinct HLA-A2/melanocyte-melanoma tumor-antigen peptide complexes. J Immunol 2001;167:3276–84. 10.4049/JIMMUNOL.167.6.3276. 11544315

[btag551-B60] Springer I , Tickotsky N, Louzoun Y. Contribution of T cell receptor alpha and beta CDR3, MHC typing, V and J genes to peptide binding prediction. Front Immunol 2021;12:664514. 10.3389/FIMMU.2021.664514. 33981311 PMC8107833

[btag551-B61] Szeto C , Lobos CA, Nguyen AT et al TCR recognition of peptide–MHC-I: rule makers and breakers. Int J Mol Sci 2020;22:68. 10.3390/ijms22010068. 33374673 PMC7793522

[btag551-B62] Tanno H , Gould TM, McDaniel JR et al Determinants governing T cell receptor α/β-chain pairing in repertoire formation of identical twins. Proc Natl Acad Sci 2020;117(1):532–40. 10.1073/pnas.1915008117. 31879353 PMC6955297

[btag551-B63] Turner SJ , Kedzierska K, Komodromou H et al Lack of prominent peptide–major histocompatibility complex features limits repertoire diversity in virus-specific CD8+ T cell populations. Nat Immunol 2005;6:382–9. 10.1038/ni1175. 15735650

[btag551-B64] Turner SJ , Doherty PC, McCluskey J et al Structural determinants of T-cell receptor bias in immunity. Nat Rev Immunol 2006;6:883–94.. 10.1038/nri1977. 17110956

[btag551-B65] Valkenburg SA , Gras S, Guillonneau C et al Preemptive priming readily overcomes structure-based mechanisms of virus escape. Proc Natl Acad Sci U S A 2013;110:5570–5. 10.1073/PNAS.1302935110. 23493558 PMC3619348

[btag551-B66] Weigt M , White RA, Szurmant H et al Identification of direct residue contacts in protein–protein interaction by message passing. Proc Natl Acad Sci U S A 2009;106:67–72. 10.1073/pnas.0805923106. 19116270 PMC2629192

[btag551-B67] Zhong W , Dixit SB, Mallis RJ et al CTL recognition of a protective immunodominant influenza a virus nucleoprotein epitope utilizes a highly restricted Vβ but diverse Vα repertoire: functional and structural implications. J Mol Biol 2007;372:535–48. 10.1016/J.JMB.2007.06.057. 17658550

