# Supplementary material for: Systematic analysis of CDR contacts and pairing constraints between T cell receptor αβ chains
Source: Bioinformatics. 2026 Jul 22;42(8):btag551. doi: 10.1093/bioinformatics/btag551 (PMC13447020; doi:10.1093/bioinformatics/btag551)
Supplement: Supplementary file 1 [file btag551_supplementary_data.zip › supplementary_figures.pdf]

## Supplementary material

| Epitope | MHC gene | Number of entries | Unique CDR3$\alpha$ | Unique CDR3$\beta$ | Number of individuals |
|---|---|---|---|---|---|
| **ASNENMETM** | H2-Kb | 196 | 155 | 108 | 26 |
| **ATDALMTGF** | HLA-A*01:01 | 125 | 99 | 111 | 1 |
| **AVFDRKSDAK** | HLA-A*11 | 1699 | 1552 | 1584 | 7 |
| subsample 0 | | 700 | 665 | 672 | 6 |
| subsample 1 | | 700 | 669 | 675 | 6 |
| subsample 2 | | 700 | 658 | 670 | 5 |
| subsample 3 | | 700 | 662 | 678 | 7 |
| subsample 4 | | 700 | 660 | 678 | 6 |
| **CINGVCWTV** | HLA-A*02:01 | 226 | 214 | 218 | 7 |
| **ELAGIGILTV** | HLA-A*02 | 374 | 344 | 365 | 9 |
| **GILGFVFTL** | HLA-A*02 | 1853 | 1077 | 978 | 51 |
| subsample 0 | | 700 | 467 | 445 | 47 |
| subsample 1 | | 700 | 489 | 447 | 46 |
| subsample 2 | | 700 | 484 | 429 | 47 |
| subsample 3 | | 700 | 481 | 442 | 45 |
| subsample 4 | | 700 | 496 | 450 | 46 |
| **GLCTLVAML** | HLA-A*02:01 | 342 | 228 | 238 | 18 |
| **HGIRNASFI** | H-2Db | 243 | 187 | 180 | 13 |
| **IVTDFSVIK** | HLA-A*11 | 704 | 526 | 517 | 7 |
| **KSKRTPMGF** | HLA-B*57:01 | 98 | 85 | 70 | 1 |
| **LLWNGPMAV** | HLA-A*02:01 | 235 | 202 | 220 | 2 |
| **LSLRNPILV** | H-2Db | 127 | 109 | 111 | 12 |
| **LTDEMIAQY** | HLA-A*01:01 | 124 | 115 | 119 | 2 |
| **NLVPMVATV** | HLA-A*02 | 348 | 301 | 314 | 41 |
| **RAKFKQLL** | HLA-B*08:01 | 1200 | 649 | 650 | 3 |
| subsample 0 | | 700 | 401 | 420 | 3 |
| subsample 1 | | 700 | 417 | 417 | 3 |
| subsample 2 | | 700 | 420 | 411 | 3 |
| subsample 3 | | 700 | 429 | 418 | 3 |
| subsample 4 | | 700 | 403 | 410 | 3 |
| **RLRAEAQVK** | HLA-A*03:01 | 412 | 389 | 394 | 4 |
| **SPRWYFYYL** | HLA-B*07:02 | 175 | 169 | 174 | 10 |
| **SSLENFRAYV** | H-2Db | 349 | 240 | 285 | 18 |
| **SSPPMFRV** | H-2Kb | 133 | 100 | 58 | 14 |
| **SSYRRPVGI** | H-2Kb | 177 | 148 | 153 | 32 |
| **TTDPSFLGRY** | HLA-A*01:01 | 242 | 231 | 232 | 1 |
| **YLQPRTFLL** | HLA-A*02:01 | 331 | 284 | 303 | 9 |

Table S1: **Summary of VDJDb set used.** For large epitopes that were subsampled for pairing, composition of each subsample is shown. MHC annotation is reported at the maximum level common to all entries for each epitope in the preprocessed VDJDb set used for all analyses.

| | background | Tanno::A1::naive | ASNENMETM | ATDALMTGF | AVFDRKSDAK | CINGVCWTV | ELAGIGILTV | GILGFVFTL | GLCTLVAML | HGIRNASFI | IVTDFSVIK | KSKRTPMGF |
|---|---|---|---|---|---|---|---|---|---|---|---|---|
| **Ja-Va** | 0.33 | 0.07 | 0.42 | 0.65 | 0.12 | 0.15 | 0.05 | 0.53 | 0.56 | 0.65 | 0.5 | 0.44 |
| **CDR3a-Va** | 3.93 | 2.84 | 3.62 | 5.87 | 2.88 | 3.7 | 1.48 | 5.43 | 5.3 | 4.9 | 5.41 | 4.26 |
| **CDR3a-Ja** | 9.21 | 9.3 | 9.04 | 9.39 | 8.61 | 7.05 | 7.16 | 9.14 | 9.33 | 8.09 | 9.84 | 6.96 |
| **Vb-Va** | 0.17 | 0.0 | 0.44 | 0.55 | 0.04 | 0.17 | 0.02 | 0.05 | 0.38 | 0.66 | -0.01 | 0.33 |
| **Vb-Ja** | 0.19 | 0.0 | 0.41 | 0.65 | 0.02 | 0.01 | 0.03 | 0.15 | 0.41 | 0.61 | 0.01 | 0.21 |
| **Vb-CDR3a** | 0.86 | 0.01 | 2.21 | 5.06 | 0.17 | 0.87 | 0.32 | 0.6 | 2.58 | 3.73 | 0.24 | 2.05 |
| **Jb-Va** | 0.07 | -0.0 | 0.46 | 0.23 | 0.0 | 0.17 | 0.02 | 0.19 | 0.15 | 0.34 | -0.02 | 0.35 |
| **Jb-Ja** | 0.04 | -0.0 | 0.3 | 0.16 | 0.0 | 0.04 | 0.02 | 0.14 | 0.3 | 0.22 | 0.01 | 0.27 |
| **Jb-CDR3a** | 0.22 | -0.02 | 1.48 | 1.35 | 0.01 | 0.71 | 0.14 | 1.03 | 1.61 | 1.59 | 0.14 | 1.79 |
| **Jb-Va** | 0.12 | 0.01 | 0.62 | 0.36 | 0.06 | 0.09 | 0.01 | 0.09 | 0.52 | 0.43 | 0.34 | 0.38 |
| **CDR3b-Va** | 0.68 | 0.01 | 3.81 | 3.64 | 0.03 | 1.51 | 0.43 | 1.04 | 1.81 | 3.02 | -0.06 | 3.72 |
| **CDR3b-Ja** | 0.64 | -0.01 | 3.14 | 3.1 | 0.06 | 0.37 | 0.03 | 1.33 | 2.86 | 2.44 | 0.1 | 2.72 |
| **CDR3b-CDR3a** | 2.86 | 0.05 | 15.28 | 20.71 | 0.2 | 6.34 | 1.38 | 7.64 | 16.06 | 15.17 | 0.88 | 20.57 |
| **CDR3b-Vb** | 2.98 | 1.85 | 5.34 | 5.0 | 1.77 | 2.27 | 1.52 | 2.9 | 7.01 | 4.61 | 4.46 | 4.32 |
| **CDR3b-Jb** | 5.87 | 5.6 | 7.82 | 6.11 | 6.04 | 5.27 | 5.63 | 6.03 | 8.73 | 7.91 | 6.87 | 7.18 |

| | LLWNGPMAV | LSLRNPILV | LTDEMIAQY | NLVPMVATV | RAKFKQLL | RLRAEAQVK | SPRWYFYYL | SSLENFRAYV | SSPPMFRV | SSYRRPVGI | TTDPSFLGRY | YLQPRTFLL |
|---|---|---|---|---|---|---|---|---|---|---|---|---|
| **Ja-Va** | 0.27 | 0.27 | 0.29 | 0.33 | 0.71 | 0.09 | 0.21 | 0.55 | 0.47 | 0.38 | 0.28 | 0.46 |
| **CDR3a-Va** | 3.67 | 2.75 | 2.86 | 3.91 | 5.92 | 2.69 | 2.55 | 4.99 | 4.16 | 3.77 | 3.56 | 4.26 |
| **CDR3a-Ja** | 5.62 | 7.67 | 6.38 | 8.41 | 10.34 | 7.15 | 6.33 | 9.05 | 8.05 | 8.17 | 7.0 | 8.29 |
| **Vb-Va** | 0.02 | 0.1 | 0.27 | 0.16 | 0.05 | 0.02 | 0.15 | 0.44 | 0.19 | 0.19 | 0.2 | 0.2 |
| **Vb-Ja** | 0.03 | 0.08 | 0.16 | 0.19 | 0.09 | 0.01 | 0.11 | 0.43 | 0.4 | 0.09 | 0.17 | 0.24 |
| **Vb-CDR3a** | 0.24 | 0.89 | 1.84 | 1.31 | 0.44 | -0.02 | 0.53 | 2.15 | 1.88 | 0.66 | 0.92 | 1.52 |
| **Jb-Va** | 0.14 | 0.09 | 0.19 | 0.16 | -0.01 | 0.01 | 0.05 | 0.18 | 0.19 | 0.17 | 0.05 | 0.34 |
| **Jb-Ja** | 0.08 | 0.16 | 0.08 | 0.13 | 0.01 | 0.04 | 0.06 | 0.2 | 0.26 | 0.07 | 0.07 | 0.26 |
| **Jb-CDR3a** | 0.48 | 0.46 | 0.31 | 0.73 | 0.05 | 0.02 | 0.47 | 0.93 | 1.39 | 0.58 | 0.36 | 1.64 |
| **Jb-Va** | 0.22 | 0.2 | 0.22 | 0.15 | 0.37 | 0.02 | 0.15 | 0.2 | 0.34 | 0.19 | 0.09 | 0.23 |
| **CDR3b-Va** | 1.13 | 0.49 | 1.73 | 1.27 | -0.02 | -0.08 | 1.01 | 1.89 | 2.21 | 1.14 | 0.25 | 2.44 |
| **CDR3b-Ja** | 0.65 | 1.0 | 1.46 | 1.28 | 0.06 | 0.03 | 0.61 | 2.26 | 3.85 | 0.62 | 0.64 | 2.13 |
| **CDR3b-CDR3a** | 4.53 | 3.43 | 6.93 | 6.87 | 0.94 | -0.2 | 3.46 | 10.17 | 20.32 | 3.0 | 3.75 | 13.86 |
| **CDR3b-Vb** | 2.38 | 2.12 | 2.35 | 2.76 | 4.65 | 1.44 | 2.88 | 2.91 | 5.87 | 2.31 | 2.0 | 3.57 |
| **CDR3b-Jb** | 5.31 | 6.35 | 6.2 | 6.15 | 6.96 | 5.81 | 5.64 | 5.57 | 5.53 | 5.74 | 5.21 | 5.64 |

Table S2: **Estimated MI for all pairs in all epitopes and background.** The estimated values for MI, corrected for shuffled background are shown for each repertoire for all pairs of elements.

| TSource + TSubtype | | |
|---|---|---|
| | Median | SD |
| normal-none | 0.364900 | 0.600487 |
| normal-unstimulated | 0.229974 | 0.119341 |
| normal-memory | 0.561325 | 0.479257 |
| normal-AIM+ | 0.412796 | 0.072385 |
| PBMC-none | 0.201735 | 0.290744 |
| PBMC-unstimulated | 0.165075 | 0.113596 |
| PBMC-includes_naive | 0.165477 | 0.115167 |
| PBMC-memory | 0.243416 | 0.480929 |
| PBMC-antigen_stimulated | 0.254005 | 0.268769 |
| PBMC-AIM+ | 0.789441 | 0.948916 |
| PBMC-tetramer_sorted | 0.333761 | 0.709520 |
| PBMC_and_tissue-none | 0.224987 | 0.259833 |
| PBMC_and_tissue-AIM+ | 0.200046 | 0.109815 |
| tumor-none | 0.294508 | 1.358885 |
| tumor-AIM+ | 1.538966 | 0.876899 |
| tumor-tetramer_sorted | 0.473903 | 0.334814 |

| TType + TSubtype | | |
|---|---|---|
| | Median | SD |
| cd3+-none | 0.223696 | 0.390664 |
| cd3+-antigen_stimulated | 0.262421 | 0.265876 |
| cd3+-AIM+ | 1.200473 | 0.819860 |
| cd4+-none | 0.176644 | 0.486733 |
| cd4+-unstimulated | 0.178039 | 0.117000 |
| cd4+-includes_naive | 0.178204 | 0.659942 |
| cd4+-memory | 0.222327 | 0.125426 |
| cd4+-antigen_stimulated | 0.217867 | 0.291887 |
| cd4+-AIM+ | 0.433065 | 0.630574 |
| cd8+-none | 0.369232 | 0.733441 |
| cd8+-memory | 0.679312 | 1.324472 |
| cd8+-AIM+ | 0.704619 | 1.066032 |
| cd8+-tetramer_sorted | 0.335994 | 0.699384 |
| unsorted-none | 0.296757 | 1.751085 |
| unsorted-includes_naive | 0.165436 | 0.103030 |
| unsorted-memory | 0.239314 | 0.115067 |
| unsorted-antigen_stimulated | 0.376644 | 0.212038 |

Table S3: **Median and standard deviation of estimated MI for T cell populations from OTS database in Fig S10.**

| Epitope | Number of pairs selected as training set | Number of correct pairs in training | % of correct pairs in training |
|---|---|---|---|
| **ASNENMETM** | 78 | 51 | 65.4% |
| **ATDALMTGF** | 5 | 0 | 0% |
| **AVFDRKSDAK** | | | |
| subsample 0 | 5 | 3 | 60.0% |
| subsample 1 | 9 | 1 | 11.1% |
| subsample 2 | 5 | 4 | 80.0% |
| subsample 3 | 17 | 9 | 52.9% |
| subsample 4 | 12 | 1 | 8.3% |
| **CINGVCWTV** | 5 | 0 | 0% |
| **ELAGIGILTV** | 5 | 1 | 20% |
| **GILGFVFTL** | | | |
| subsample 0 | 10 | 9 | 90.0% |
| subsample 1 | 11 | 10 | 90.9% |
| subsample 2 | 85 | 33 | 38.8% |
| subsample 3 | 35 | 24 | 68.6% |
| subsample 4 | 12 | 12 | 100.0% |
| **GLCTLVAML** | 84 | 34 | 40.5% |
| **HGIRNASFI** | 5 | 0 | 0% |
| **IVTDFSVIK** | 107 | 61 | 57.0% |
| **KSKRTPMGF** | 5 | 3 | 60.0% |
| **LLWNGPMAV** | 5 | 0 | 0% |
| **LSLRNPILV** | 80 | 29 | 36.3% |
| **LTDEMIAQY** | 5 | 1 | 20.0% |
| **NLVPMVATV** | 88 | 51 | 58.0% |
| **RAKFKQLL** | | | |
| subsample 0 | 5 | 4 | 80.0% |
| subsample 1 | 5 | 2 | 40.0% |
| subsample 2 | 5 | 3 | 60.0% |
| subsample 3 | 5 | 2 | 40% |
| subsample 4 | 5 | 2 | 40.0% |
| **RLRAEAQVK** | 5 | 0 | 0% |
| **SPRWYFYYL** | 5 | 1 | 20.0% |
| **SSLENFRAYV** | 10 | 9 | 90.0% |
| **SSPPMFRV** | 94 | 48 | 51.1% |
| **SSYRRPVGI** | 101 | 31 | 30.7% |
| **TTDPSFLGRY** | 5 | 1 | 20.0% |
| **YLQPRTFLL** | 5 | 3 | 60% |

Table S4: **Summary of stable pairs from GA used as training set for MI-IPA.** The number of pairs selected from the GA to act as training set for the MI-IPA are shown for each epitope, together with the number of these pairs which are correct.

| | N | Largest ID size | Repertoire MI (nats) | $R^2$ | $R^2_{\text{adj}}$ | F-score p-value |
|---|---|---|---|---|---|---|
| **No-learning background:** confidence = none; $\lambda = 1.0$; $\theta = 0.6$ | 0.1250 (*) | -0.0843 | 0.0151 | 0.232 | 0.104 | 0.180 |
| **Learning model:** confidence = none; $\lambda = 0.6$; $\theta = 0.6$ | 0.1396 | -0.1193 (*) | 0.0479 | 0.347 | 0.238 | 0.0489 |
| **Theoretical best:** confidence = none; $\lambda = 0.6$; $\theta = 0.6$ | -0.1176 | -0.0964 | 0.0389 | 0.591 | 0.522 | 0.000904 |

Table S5: **Factors affecting MI-IPA performance.** A multivariate linear regression was calculated to evaluate the effect repertoire size (N), largest individual (Largest ID size) and total mutual information (Repertoire MI, calculated as in Figure 4 between CDR3$\alpha$ and CDR3$\beta$) on the precision. Subsamples from large repertoires are included as an average, and the repertoire MI is calculated on the complete sample. Each independent variable was normalised by subtracting the mean and dividing by the mean to make the derived coefficients more comparable ($x_{norm} = (x - \bar{x})/\bar{x}$). The $R^2$ and adjusted $R^2$ ($R^2_{\text{adj}}$) for the regression are shown, as well as the F-score p-value. P-values associated with each coefficient are shown as asterisks: ***$< 0.001$; $0.001 \leq$**$< 0.01$; $0.01 \leq$*$< 0.05$.

| | N | Largest ID size | Average degree of node | Number of large clusters | Proportion of singlets | $R^2$ | $R^2_{\text{adj}}$ | F-score p-value |
|---|---|---|---|---|---|---|---|---|
| **GA:** distance=lev; $k$=20 | -0.2870 | -0.1373 (*) | $\alpha$: 0.0332 $\beta$: 0.1084 (**) | $\alpha$: 0.2555 $\beta$: 0.1198 | $\alpha$: 0.1562 $\beta$: 0.0600 | 0.799 | 0.675 | 0.0017 |

Table S6: **Factors affecting GA performance.** A multivariate linear regression was calculated to evaluate the effect repertoire size (N), largest individual (Largest ID size) and similarity network characteristics on the precision for each model. The graph characteristics measured are: average degree of nodes, number of clusters of size $\geq 3$ and proportion of sequences that do not have any neighbours (singlets). These are calculated on the graph built by drawing edges between sequences at Levenshtein distance $\leq 3$ on both CDR3$\alpha$ and CDR3$\beta$. Epitopes for which 5 subsamples are available are averaged across subsamples, and each of the metrics is calculated on the specific subsample. Each independent variable was normalised by subtracting the mean and dividing by the mean to make the derived coefficients more comparable ($x_{norm} = (x - \bar{x})/\bar{x}$). The $R^2$ and adjusted $R^2$ ($R^2_{\text{adj}}$) for the regression are shown, as well as the F-score p-value. P-values associated with each coefficient are shown as asterisks: ***$< 0.001$; $0.001 \leq$**$< 0.01$; $0.01 \leq$*$< 0.05$.

| | **N** | **Largest ID size** | **Repertoire MI (nats)** | **Number of stable pairs in GA** | **% correct in GA stable** | $R^2$ | $R^2_{\text{adj}}$ | **F-score p-value** |
|---|---|---|---|---|---|---|---|---|
| **GA+MI-IPA model precision:** distance=lev; $k$=20; GA-thresh=0.95; re-pair=True | -0.0113 | -0.0184 | 0.0226 | 0.0793 (***) | 0.0354 | 0.818 | 0.762 | $1.92 \times 10^{-5}$ |
| **precision increase from MI-IPA:** precision of GA+MI-IPA − precision of MI-IPA | -0.0113 | -0.0184 | -0.0187 (*) | -0.0065 | 0.0160 | 0.450 | 0.278 | 0.0653 |
| **precision ratio between GA+MI-IPA and MI-IPA:** $\log_2(\frac{\text{precision of GA+MI-IPA}}{\text{precision of MI-IPA}})$ | 0.0934 | -0.3963 | -0.2557 | -0.2024 | 0.0734 | 0.290 | 0.068 | 0.310 |

Table S7: **Factors affecting GA+MI-IPA performance.** A multivariate linear regression was calculated to evaluate the effect repertoire size (N), largest individual (Largest ID size), total mutual information (Repertoire MI, calculated as in Figure 4 between CDR3$\alpha$ and CDR3$\beta$), number of stable GA pairs and proportion of the GA training set that is correct (% correct in GA stable) on the precision. Each independent variable was normalised by subtracting the mean and dividing by the mean to make the derived coefficients more comparable ($x_{norm} = (x - \bar{x})/\bar{x}$). The $R^2$ and adjusted $R^2$ ($R^2_{\text{adj}}$) for the regression are shown, as well as the F-score p-value. P-values associated with each coefficient are shown as asterisks: ***$< 0.001$; $0.001 \leq$**$< 0.01$; $0.01 \leq$*$< 0.05$.

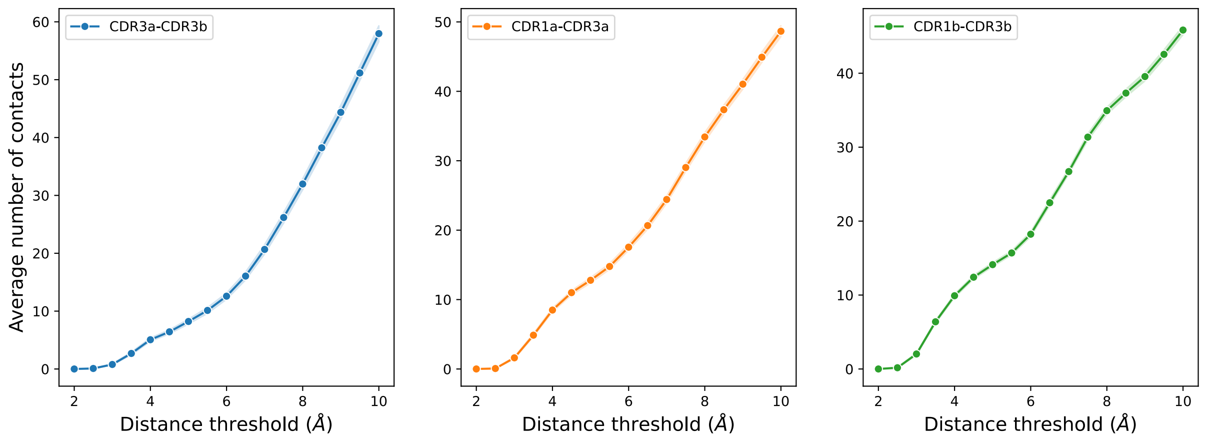


Figure S1: **The relationship between the number of inter-CDR contacts detected and the distance threshold.** Number of observed contacts between CDRs are calculated for each of 340 unique PDB structures at different threshold.

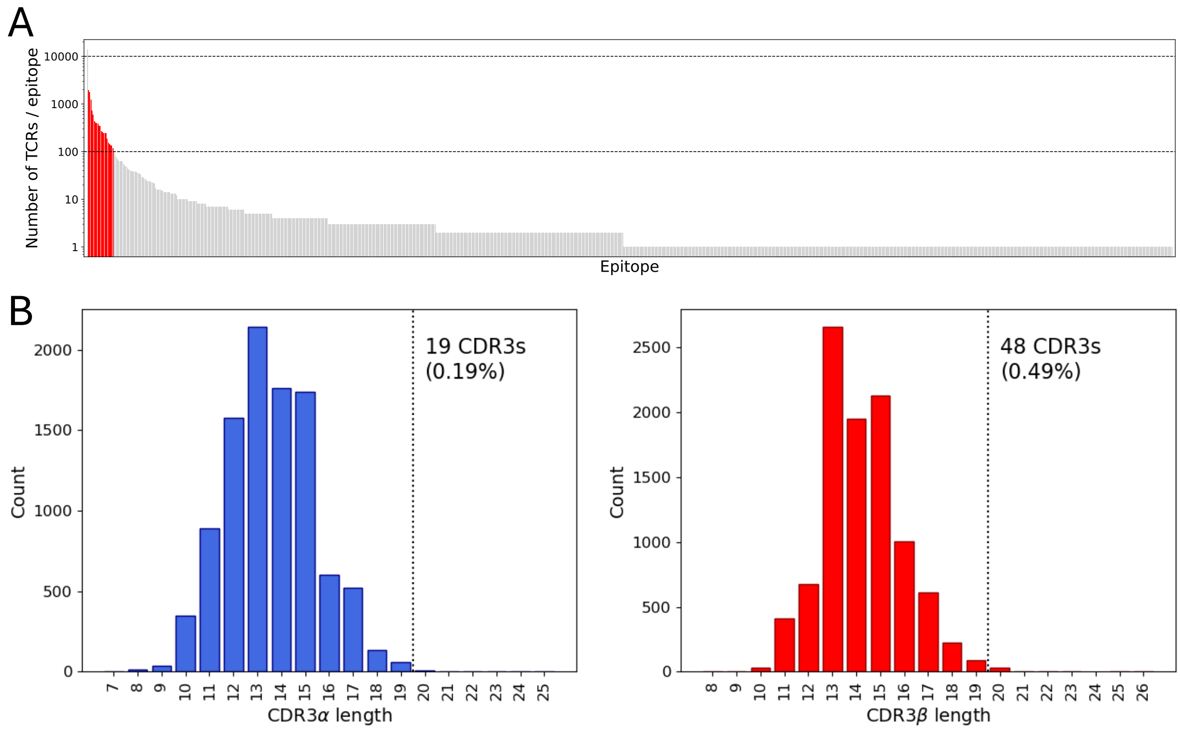


Figure S2: **TCRs and CDR3s from the VDJdb dataset included in the study. a)** Distribution of TCR counts associated with single epitopes in VDJdb. The 22 epitopes with $> 100$ and $\leq 10,000$ sequences (dashed lines, selected epitopes indicated with red bars) are used for all analyses. **b)** Distribution of CDR3 lengths (without padding) in the $\alpha$ (left) and $\beta$ (right) chains. The dotted line shows the length threshold chosen as maximum allowed length, and the text indicates how many CDR3s were removed and what percentage they correspond to.

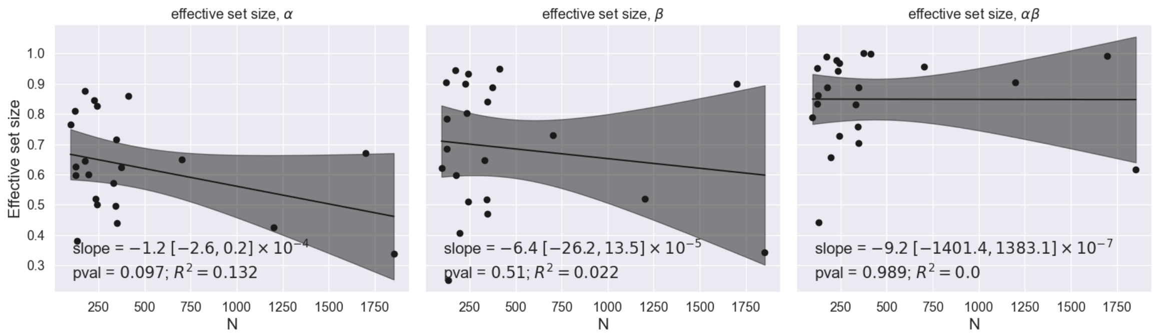


Figure S3: **Correlation of repertoire size and effective set size.** For each epitope, CDR3$\alpha$, CDR3$\beta$ and CDR3$\alpha\beta$ effective set size is correlated with total repertoire size ($N$). Effective set size was calculated by grouping all sequences with Hamming distance of $< 2$ on each chain or $< 3$ for the paired chains, normalised by $N$. Correlation was calculated by fitting a linear regression. The slope and $R^2$ of all fits are reported. 95% CI for the slope are indicated in square brackets. p-value for the F-test and $R^2$ was calculated for each fit. The shaded area represents the 95% CI of the fit.

**A. Correct $\alpha\beta$ pairs with ID**

| $\alpha$ | $\beta$ | ID |
|---|---|---|
| CASSALASLNEQFF | CASSS--LNTQYF | A |
| CASSFG-VEDEQYF | CASSS--LNTQYF | B |
| CASSALASLNEQFF | CASSIRSTDTQYF | B |

**B. Predicted $\alpha\beta$ pairs without ID**

| $\alpha$ | $\beta$ | correct |
|---|---|---|
| CASSALASLNEQFF | CASSS--LNTQYF | True |
| CASSALASLNEQFF | CASSS--LNTQYF | True |
| CASSFG-VEDEQYF | CASSIRSTDTQYF | False |

**C. Predicted $\alpha\beta$ pairs with ID**

| $\alpha$ | $\beta$ | ID | correct |
|---|---|---|---|
| CASSALASLNEQFF | CASSS--LNTQYF | A | True |
| CASSALASLNEQFF | CASSS--LNTQYF | B | False |
| CASSFG-VEDEQYF | CASSIRSTDTQYF | B | False |

Figure S4: **Example assignment with repeat sequences and ID. A)** Correct pairs to be found. The blue $\alpha$ appears twice in the two individuals, but paired with two different $\beta$. The red $\beta$ also appears twice, once paired with the blue $\alpha$. **B)** Pairs assigned by the algorithm, disregarding individual information. Here, the blue $\alpha$/red $\beta$ pair appears twice. In the precision calculation, this will be counted twice as correct, giving a precision of 0.66. **C)** Pairs assigned by the algorithm, including individual information. Here, the blue $\alpha$/red $\beta$ pair appears twice. Because the blue $\alpha$/red $\beta$ pair does not appear in individual B, the pair is considered correct only in individual A.

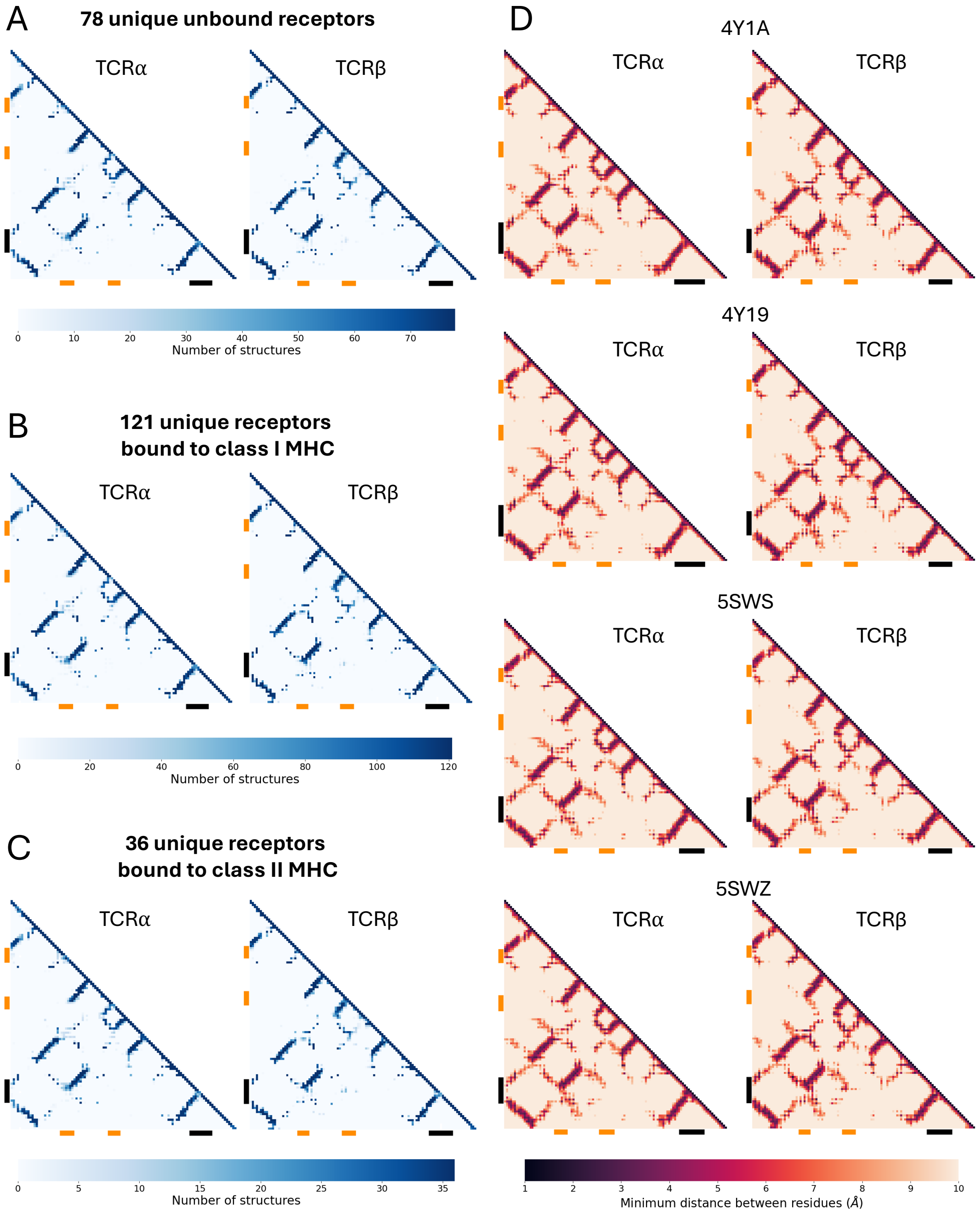


Figure S5: **Intra-chain contacts of unbound TCRs and TCRs bound to class I and class II MHC.** Intra-chain contact maps were calculated for the TCR $\alpha$ and $\beta$ chain of 78 unique unbound TCRs **(A)** and 157 unique bound TCRs to either class I **(B)** or class II MHC **(C)**. A contact was defined as minimum distance between residues $< 5$Å. The heatmap shows the number of structures that are found to make contact at each pair of positions. Only positions present in $> 75\%$ of structures according to the IMGT numbering scheme are shown. Only the bottom half of each distance matrix is shown as it is symmetrical across the diagonal. **D)** Intra-chain contact maps for the TCR $\alpha$ and $\beta$ chain for 4 TCRs known to bind MHC with reversed orientation. The distance (Å, colour bar) is calculated between all pairs of atoms in the two residues, and the minimum distance is shown. In each heatmap, the positions of CDR1, 2 (orange) and 3 (black) are highlighted.

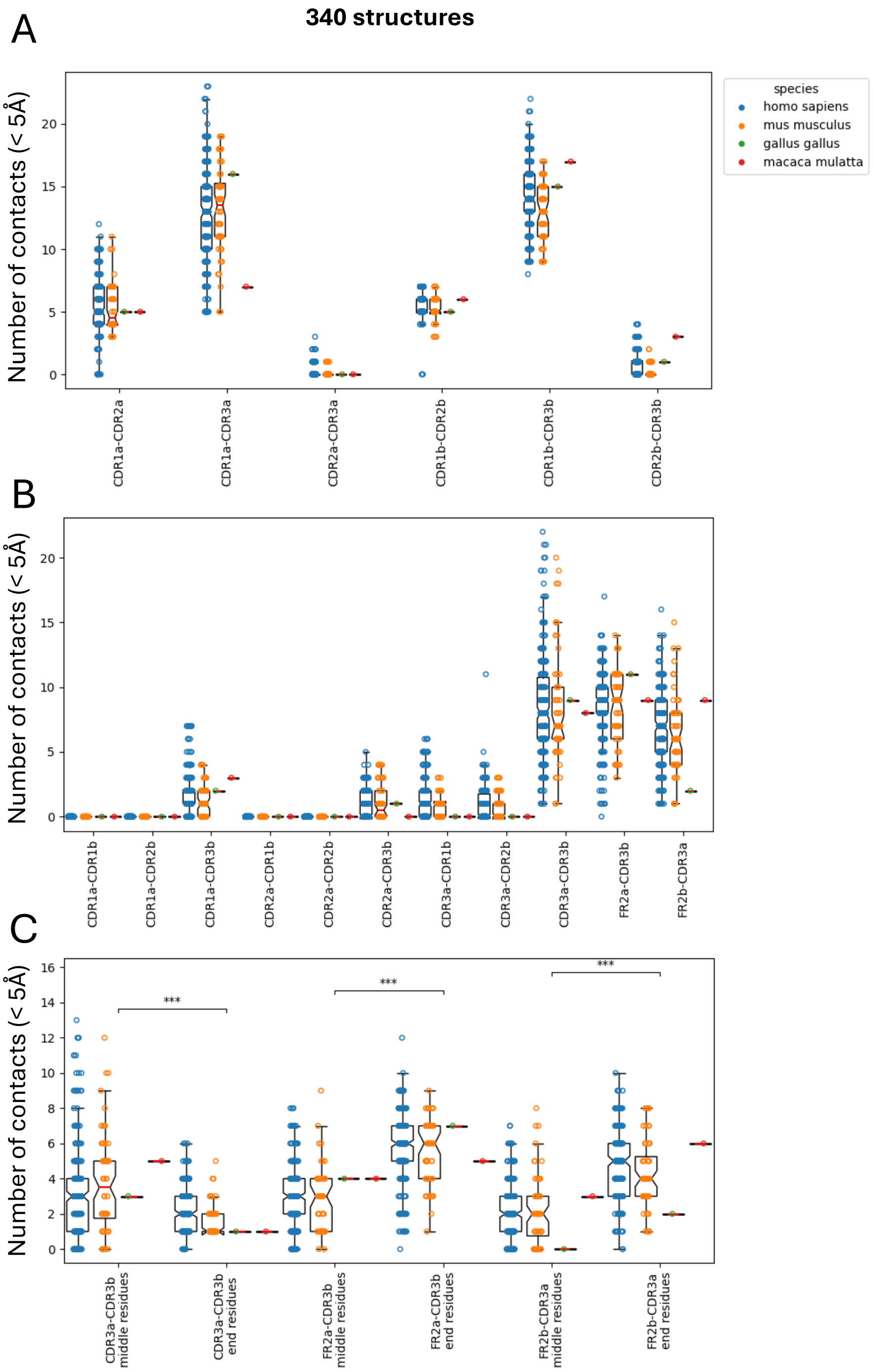


Figure S6: **Number of intra- and inter-chain contacts observed in all available structures. A, B)** Number of intra- (**A**) and inter- (**B**) chain contacts observed in each of 340 TCR structures analysed, focussing on the CDR regions and framework region (FR) 2. **C)** Number of contacts detected that occur with the CDR3 terminus ("end residues", defined as first 3 or last 3 residues) or the middle residues (defined as excluding first 3 and last 3 CDR3 residues). A contact is defined as minimum distance between residues $< 5$Å. Red lines and the notch within the boxplots represent the median, and the box limits represent the quartiles. Whiskers of the boxplots extend to 1.5 the interquartile range of the lower or upper quartile. In each set of boxplot, TCRs from various species are shown separately and colour-coded. For **C**, p-value is calculated between relevant pairs by Wilcoxon signed-rank test, ignoring grouping by species. *** = Bonferroni-corrected p-value $< 0.001$

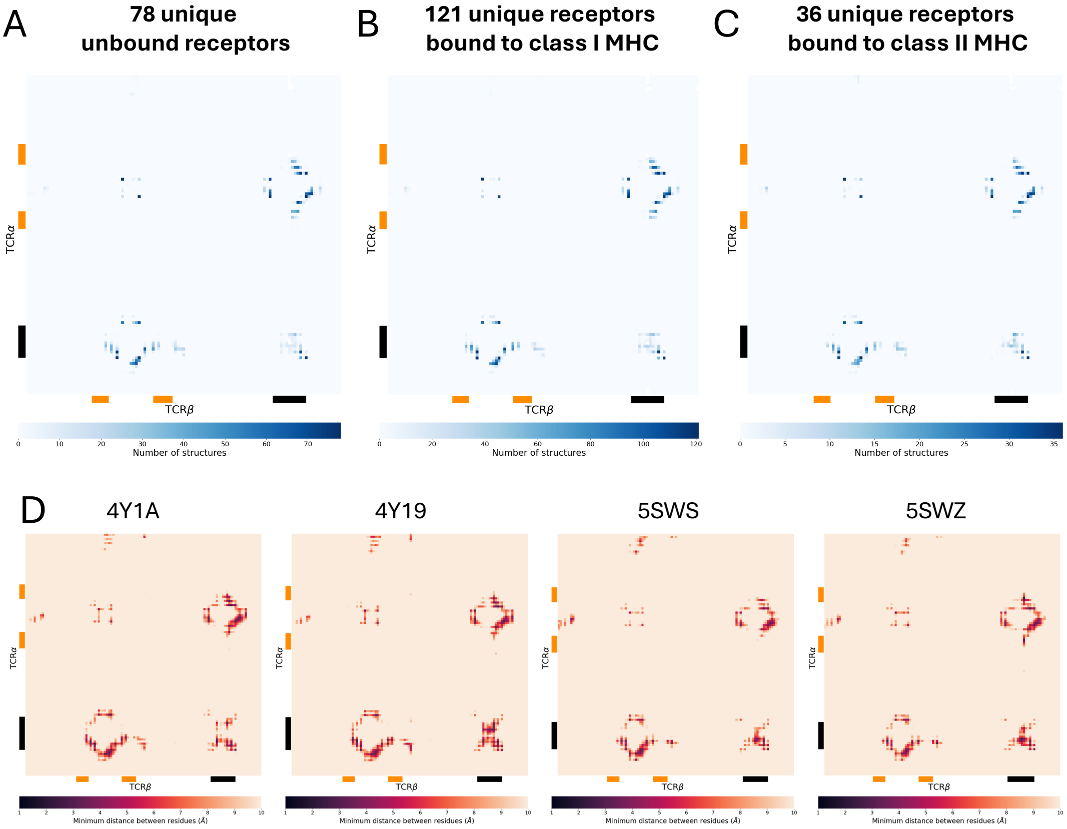


Figure S7: **Inter-chain contacts of unbound TCRs and TCRs bound to class I and class II MHC.** Inter-chain contact maps were calculated for 78 unique unbound TCRs **(A)** and 157 unique bound TCRs to either class I **(B)** or class II MHC **(C)**. A contact was defined as minimum distance between residues $< 5$Å. The heatmap shows the number of structures that are found to make contact at each pair of positions. Only positions present in $> 75\%$ of structures according to the IMGT numbering scheme are shown. **D)** Inter-chain contact maps for 4 TCRs known to bind MHC with reversed orientation. The distance (Å, colour bar) is calculated between all pairs of atoms in the two residues, and the minimum distance is shown. In each heatmap, the positions of CDR1, 2 (orange) and 3 (black) are highlighted.

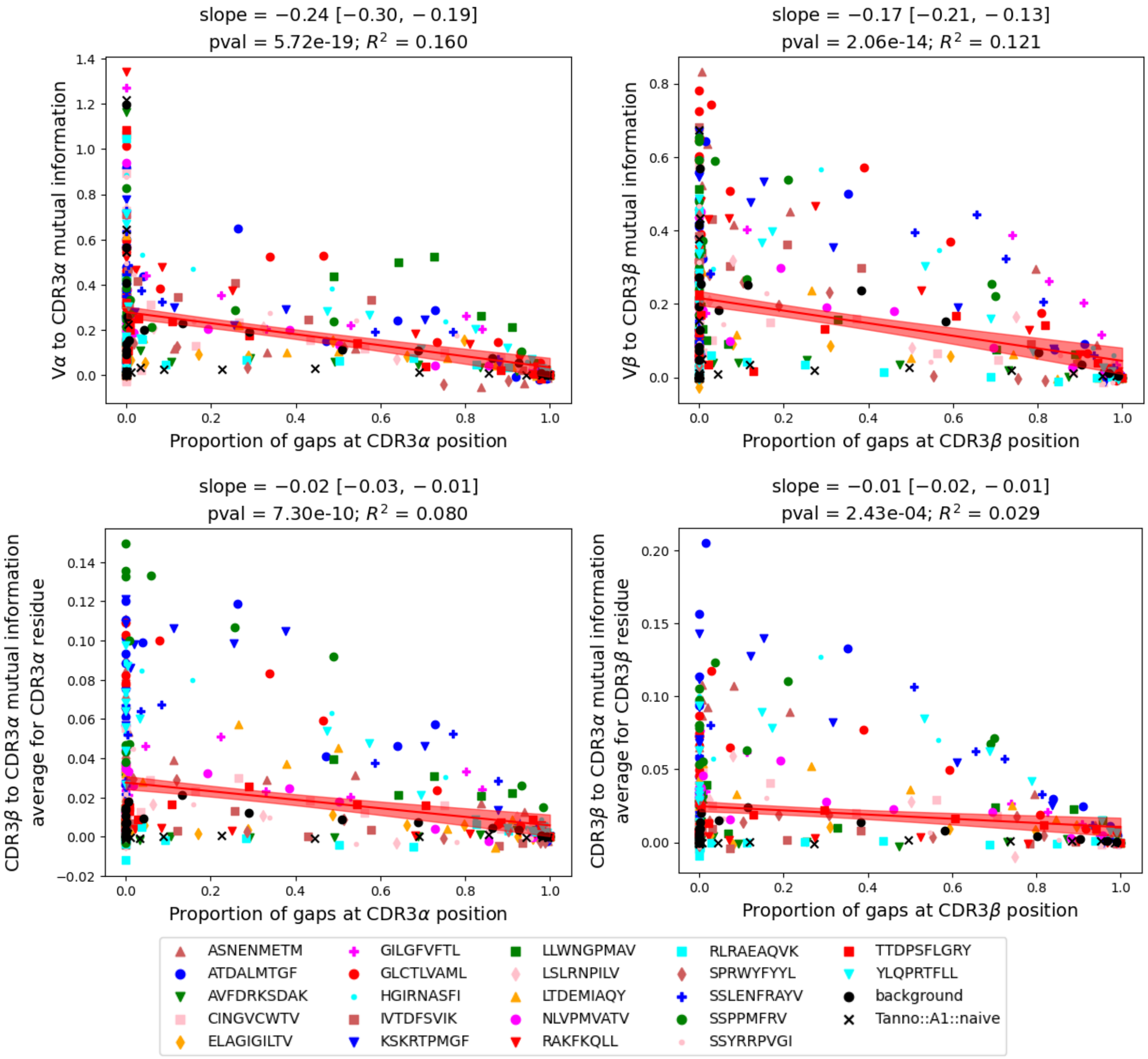


Figure S8: **Contribution of gaps to MI information.** Average MI per CDR3 ($\alpha$ on the left, $\beta$ on the right) residue with V gene (top) or CDR3 (bottom). Slope, pval and $R^2$ of shown fit are reported. The shaded area represents the 95% CI of the fit. The 95% CI of the calculated slopes are shown in square brackets.

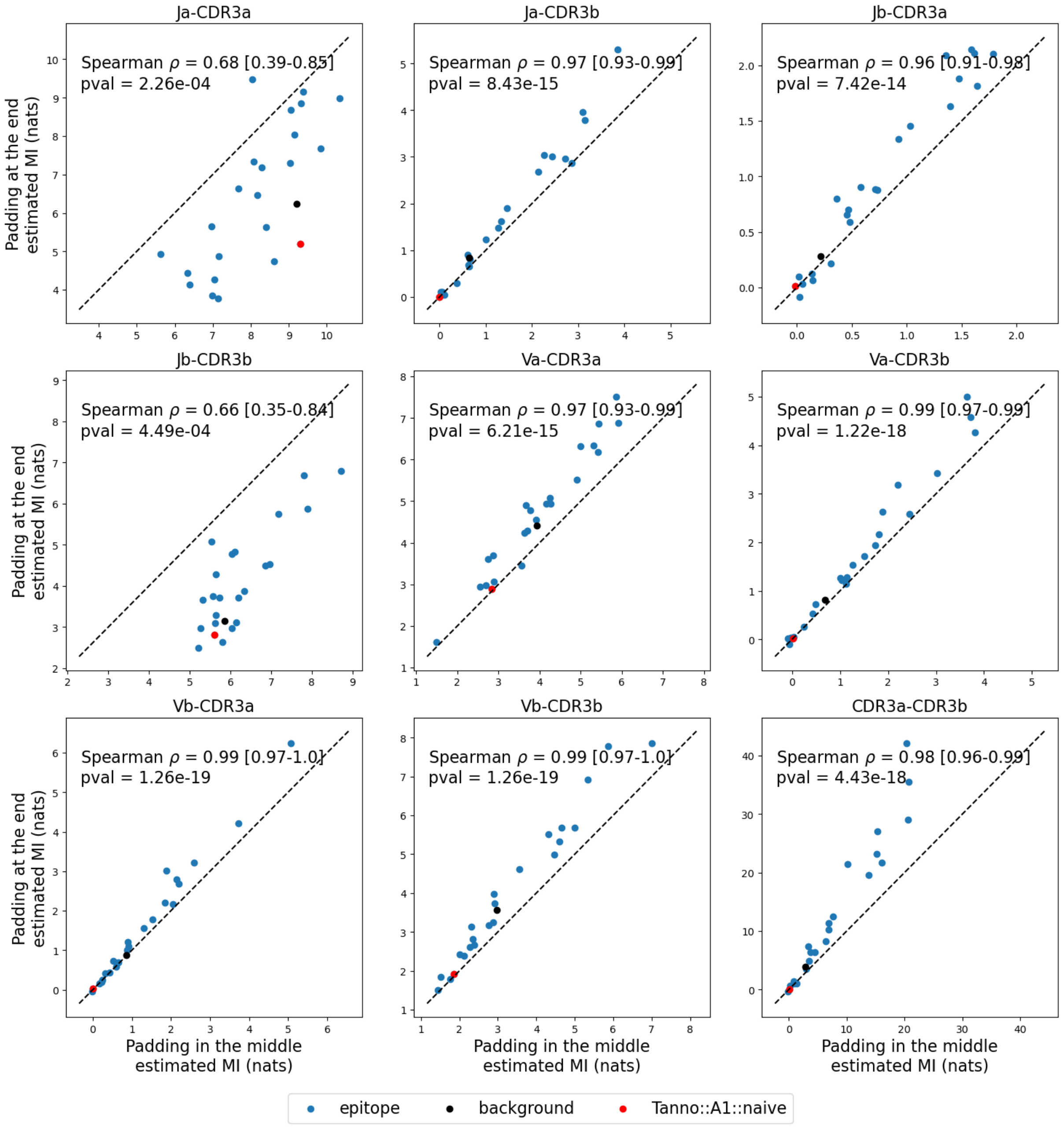


Figure S9: **Comparison of estimated MI when different padding methods are used.** For each pair of variables, the comparison of the MI estimated on CDR3s with padding in the middle (position $\frac{L}{2}$ where $L$ is the length of the CDR3 sequence) or at the end of each sequence is shown. The $y = x$ diagonal is shown with a dashed line. Only pairs of variables including the CDR3 are shown in the plot, as V and J MI estimation is not affected by the padding strategy. Spearman $\rho$ with 95% CI (in square brackets) and p-value for each correlation are reported.

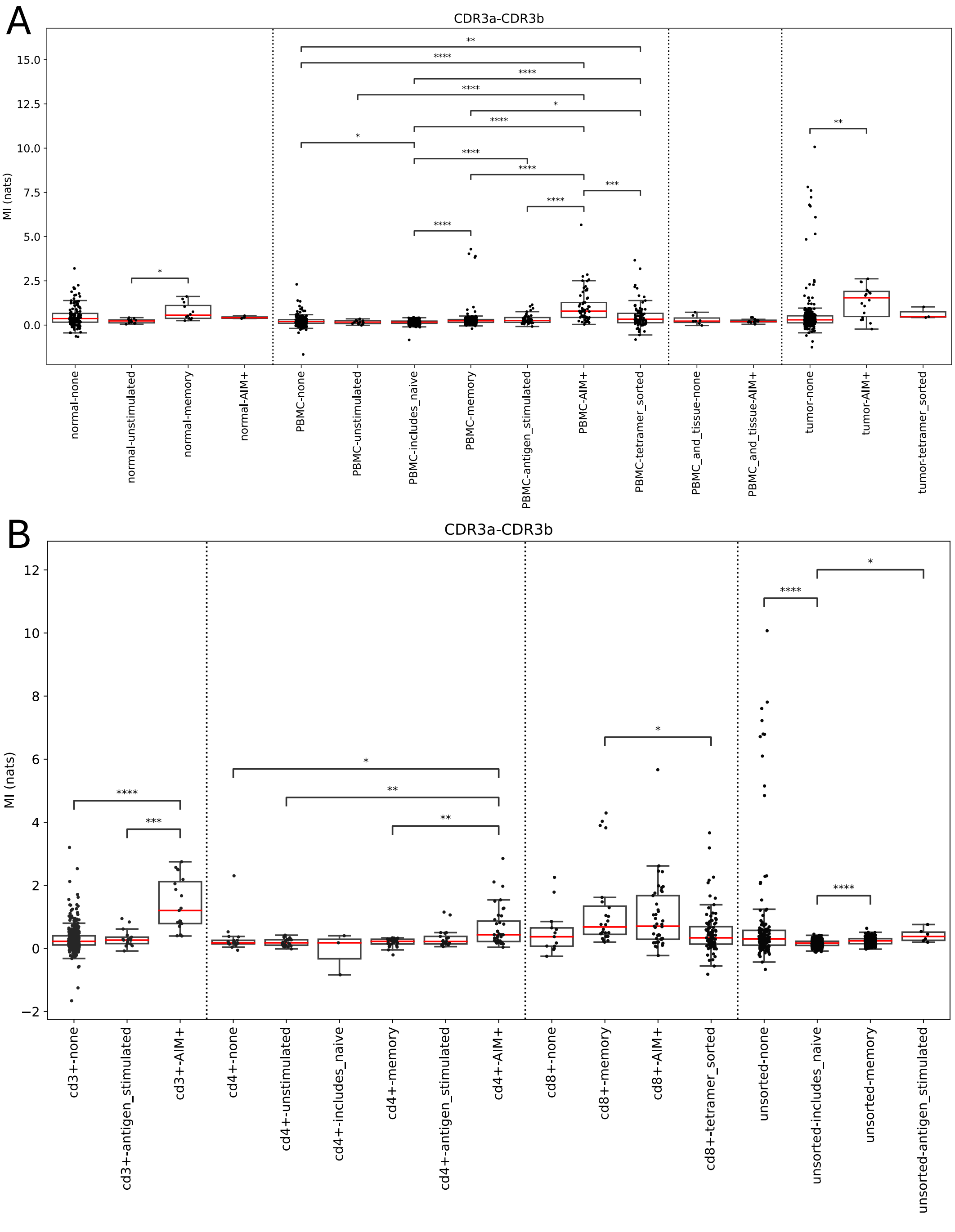


Figure S10: **MI of datasets from OTS database.** Calculated MI is shown for all included studies in the OTS databse, grouped according to the provided TSource and TSubtype (**A**) or the provided TType and TSubtype (**B**). The categories from the database are further grouped to give more meaningful larger groups for the comparisons. The edges of each boxplot show the quartiles, and the whiskers extend to 1.5 the interquartile range. Dotted lines separate comparison classes for the p-values. Mann-Whitney p-values with Bonferroni correction are calculated within each class. Significant p-values are indicated by stars: **** $< 10^{-4}$, *** $< 10^{-3}$, ** $< 10^{-2}$, * $< 0.05$. Median values and standard deviation of each group are reported in Table S3.

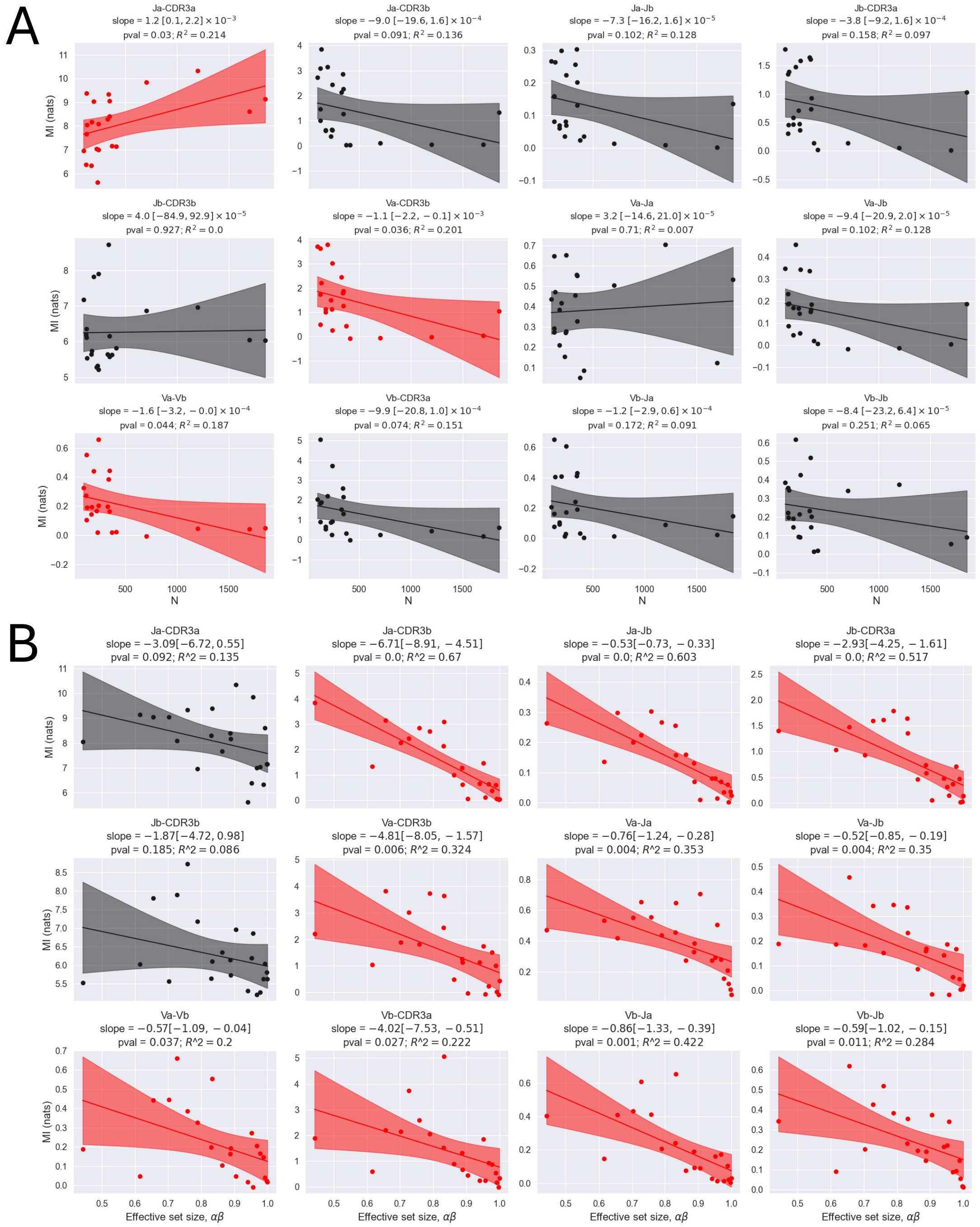


Figure S11: **Correlation between mutual information and epitope repertoire size and mutual information and effective set size.** For each epitope and each pair of variables the mutual information (MI) was estimated, correcting for finite size effects (see Methods). The correlation between MI and epitope repertoire size (N, **A**) or $\alpha\beta$ effective set size (**B**) is shown for each pair of variables. Effective set size was calculated by grouping all sequences with Hamming distance $< 3$ on the paired chain, normalised by N. Correlation was calculated by fitting a linear regression. p-value for the F-test, $R^2$ and slope were calculated for each fit. Fits that have p-value$< 0.05$ are highlighted in red. The shaded area represents the 95% CI of the fit. 95% CIs for the calculated slopes are reported in square brackets.

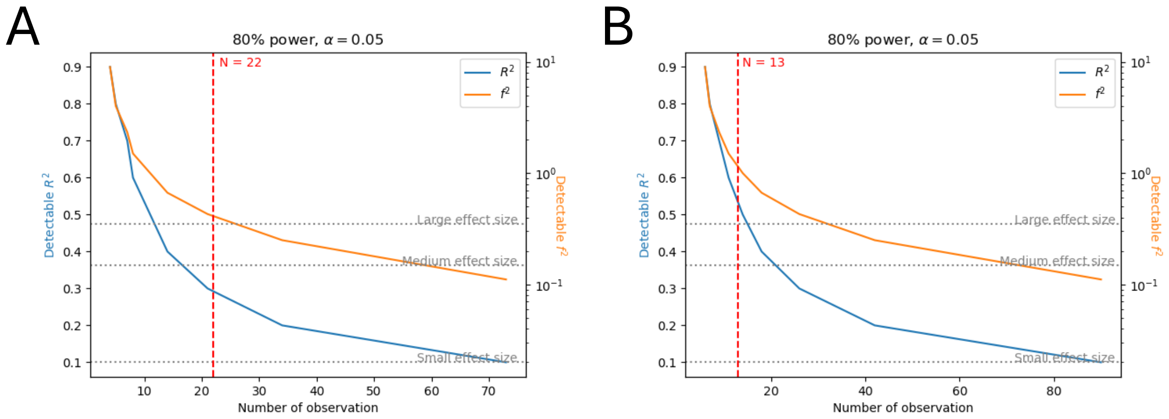


Figure S12: **Power analysis for correlation plots.** The detectable fit ($R^2$) and effect size ($f^2 = \frac{R^2}{1-R^2}$) were calculated for a range of observation numbers, given a linear (**A**, 1 degree of freedom) or a polynomial (**B**, 2 degrees of freedom) fit. The dotted lines show a small ($f^2 = 0.02$), medium ($f^2 = 0.15$) and large ($f^2 = 0.35$) effect size, as defined by **cohen˙statistical˙1988**. The red dashed lines show the number of epitopes included in the linear correlations (22) and in the polynomial correlations (13, the only epitopes for which structure is available). Both models were run with power $= 0.8$ and $\alpha = 0.05$.

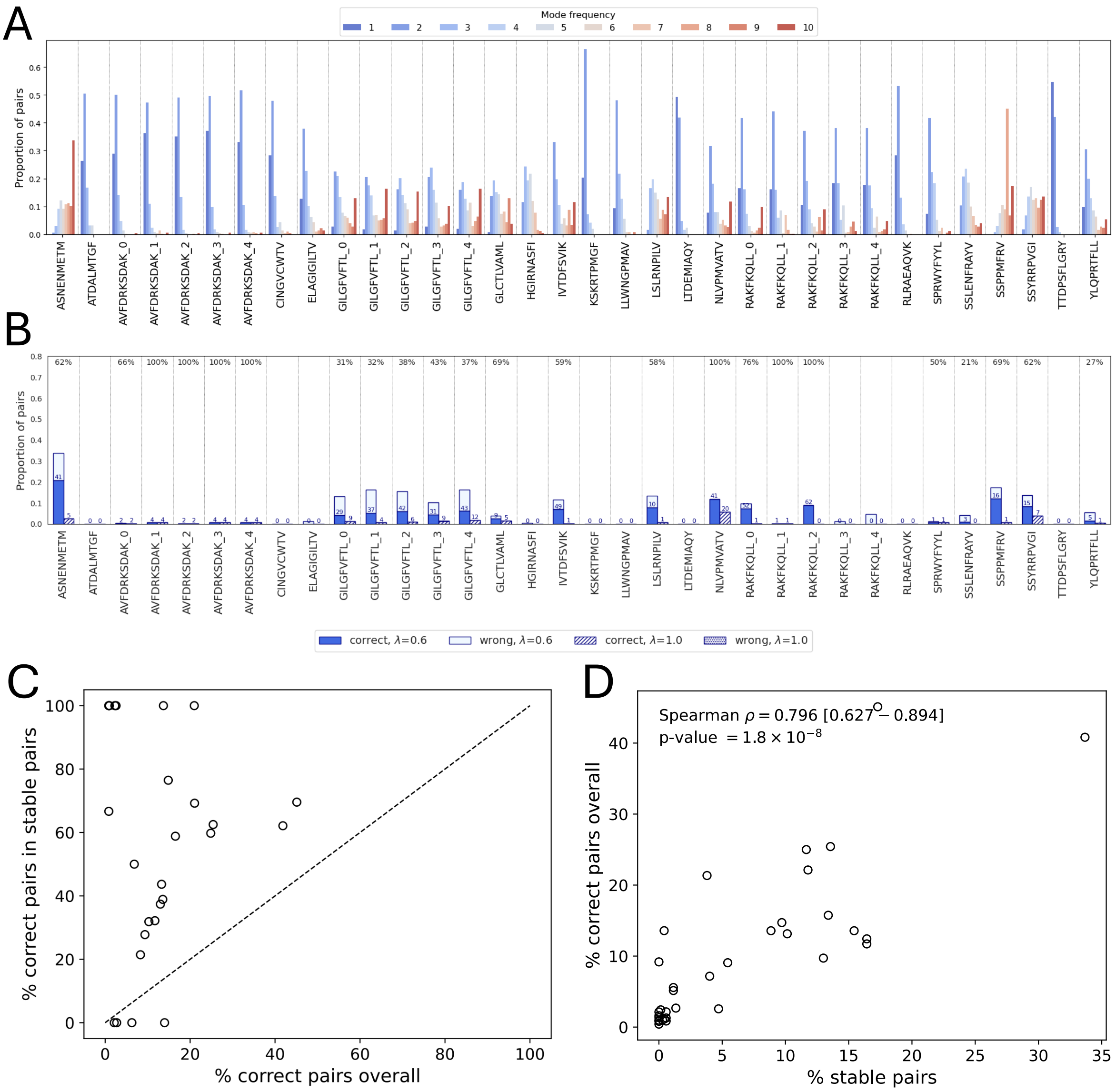


Figure S13: **Stability of the pairs assigned by the MI-IPA. A)** The bar chart shows the stability of the assignments made for each epitope. The x-axis shows the number of times an assignment is made across 10 repeats (i.e. the frequency of the mode, also colour-coded) and the y-axis shows the proportion of assignments that have that mode. The bar graph is repeated for each epitope, and for large epitopes all five subsamples are shown. The results shown correspond to pairing with $\lambda = 0.6$. **B)** The right-most bar for each epitope in **A** is broken down to show the proportion of stable assignments that are correct (with $\lambda = 0.6$ or $\lambda = 1$). The number on top of each bar indicates the number of correct pairs that were selected 10 times. The number at the top of each section indicates the percentage of correctly assigned pairs among pairs that are stably selected across repeats. Of note, some epitopes show stable pairs also in the chance expectation ($\lambda = 1$) scenario. These arise from individuals of size 1, i.e. examples where only a single $\alpha$ and a single $\beta$ are available from an individual, which will always be assigned correctly independently of how well the model is learning. **C)** Comparison of correct pairs (defined as in **B**) in the whole set and in the stable pairs for each epitope. Only epitopes with any stable pairs shown. **D)** Correlation between proportion of stable pairs (defined as in **B**) and proportion of correct pairs in each epitope. Spearman $\rho$ with 95% CIs and p-values are reported.

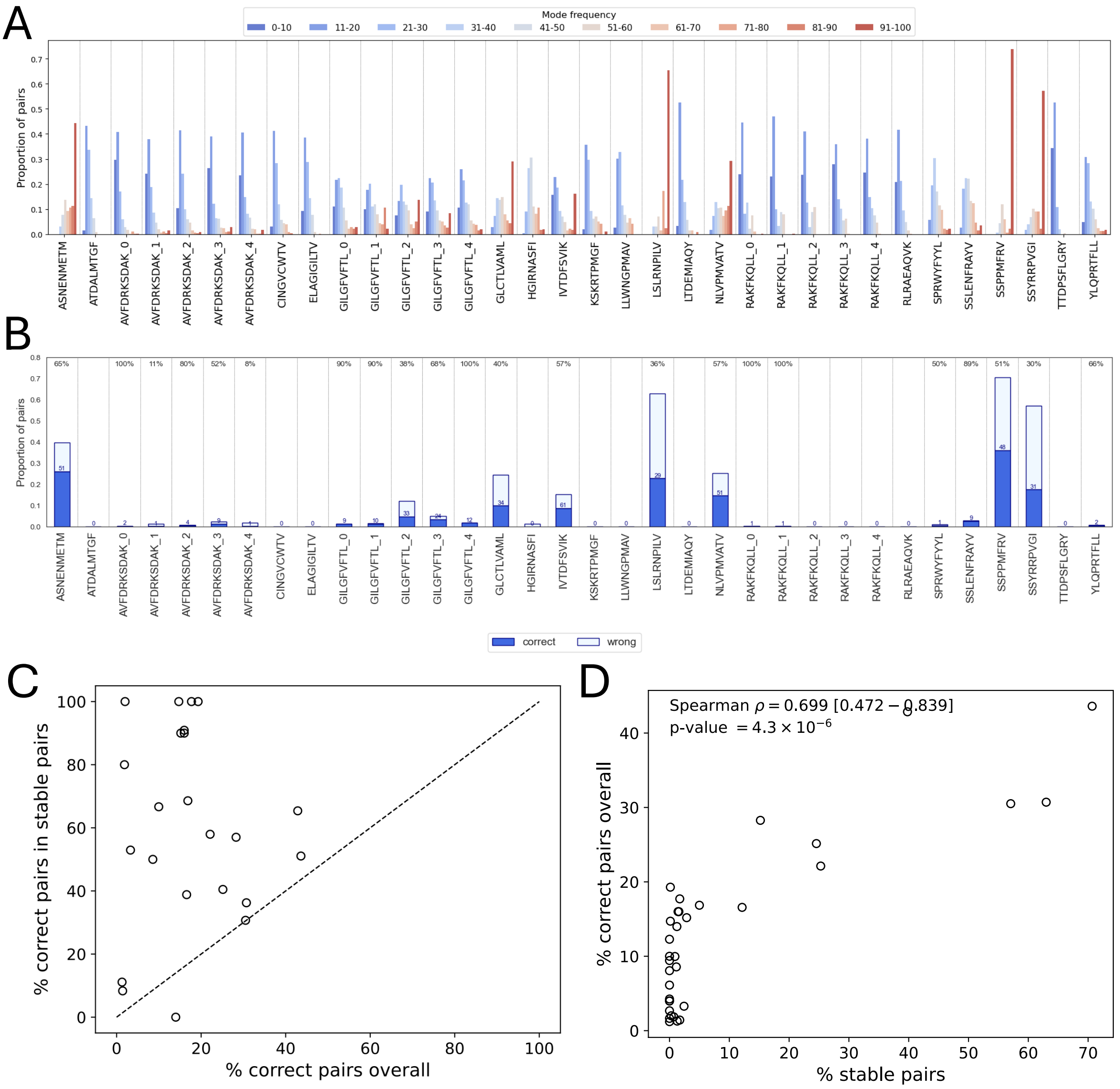


Figure S14: **Stability of the pairs assigned by the GA. A)** The bar chart shows the stability of the assignments made for each epitope. The x-axis shows the number of times an assignment is made across 100 repeats (i.e. the frequency of the mode, also colour-coded) and the y-axis shows the proportion of assignments that have that mode. The bar graph is repeated for each epitope, and for large epitopes all five subsamples are shown. The results shown correspond to GA run with $k = 20$ on Levenshtein distance. **B)** Proportion of correct assignments among the most stable pairs (selected $\geq 95$ times). The number on top of each bar indicates the number of correct pairs. The number at the top of each section indicates the percentage of correctly assigned pairs among pairs that are stably selected across repeats. **C)** Comparison of correct pairs (defined as in **B**) in the whole set and in the stable pairs for each epitope. Only epitopes with any stable pairs shown. **D)** Correlation between proportion of stable pairs (defined as in **B**) and proportion of correct pairs in each epitope. Spearman $\rho$ with 95% CIs and p-values are reported.

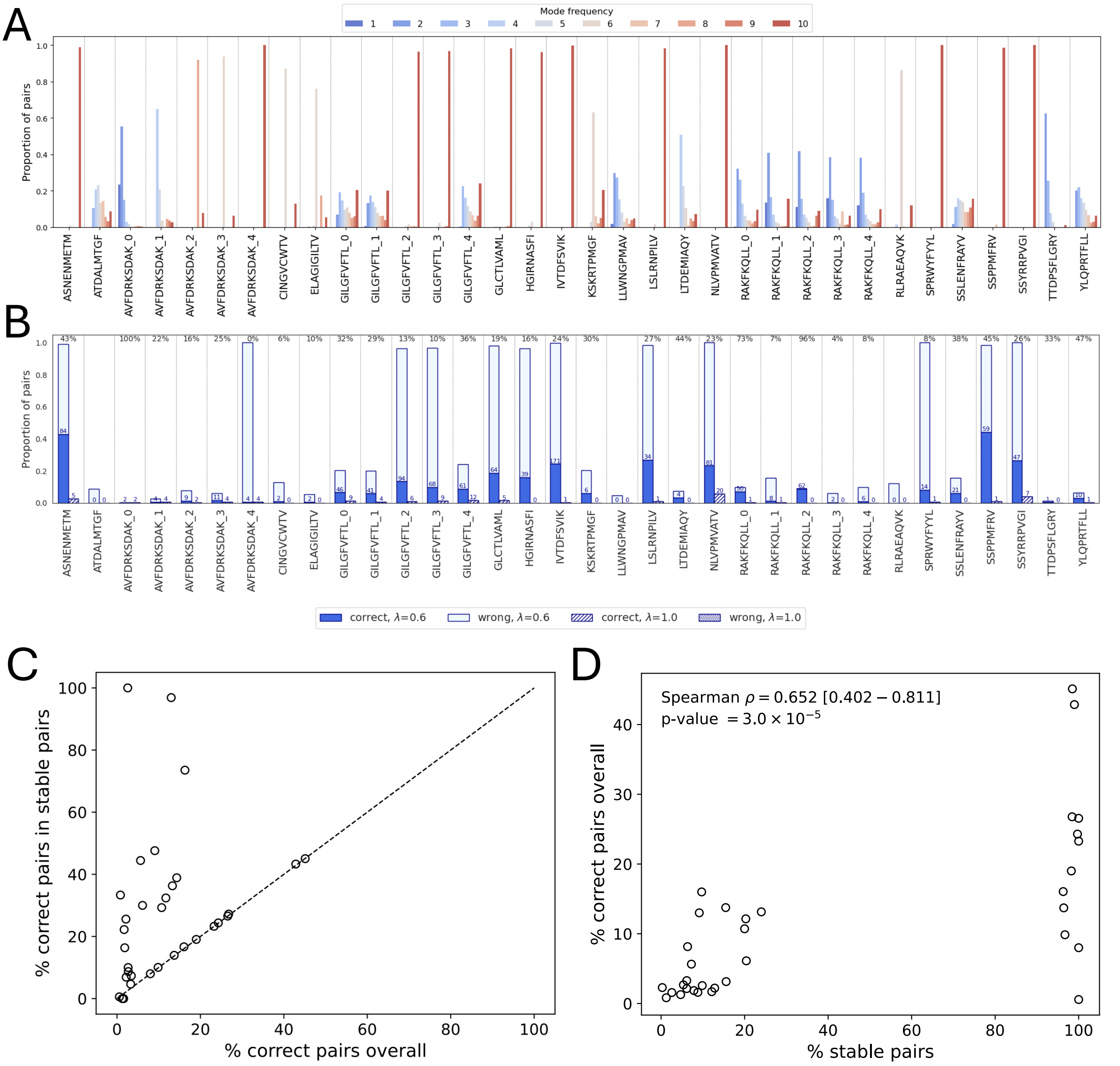


Figure S15: **Stability of the pairs assigned by the GA+MI-IPA. A)** The bar chart shows the stability of the assignments made for each epitope. The x-axis shows the number of times an assignment is made across 10 repeats (i.e. the frequency of the mode, also colour-coded) and the y-axis shows the proportion of assignments that have that mode. The bar graph is repeated for each epitope, and for large epitopes all five subsamples are shown ($\lambda = 0.6$, golden set from the GA, re-pairing allowed). **B)** The right-most bar for each epitope in **A** is broken down to show the proportion of stable assignments that are correct (MI-IPA run with $\lambda = 0.6$ or $\lambda = 1$, golden set from the GA, re-pairing allowed). The number on top of each bar indicates the number of correct pairs that were selected 10 times. The number at the top of each section indicates the percentage of correctly assigned pairs among pairs that are stably selected. Of note, some epitopes show stable pairs also in the chance expectation ($\lambda = 1$) scenario. These arise from individuals of size 1, i.e. examples where only a single $\alpha$ and a single $\beta$ are available from an individual, which will always be assigned correctly independently of how well the model is learning. **C)** Comparison of correct pairs (defined as in **B**) in the whole set and in the stable pairs for each epitope. **D)** Correlation between proportion of stable pairs (defined as in **B**) and proportion of correct pairs in each epitope. Spearman $\rho$ with 95% CIs and p-values are reported.

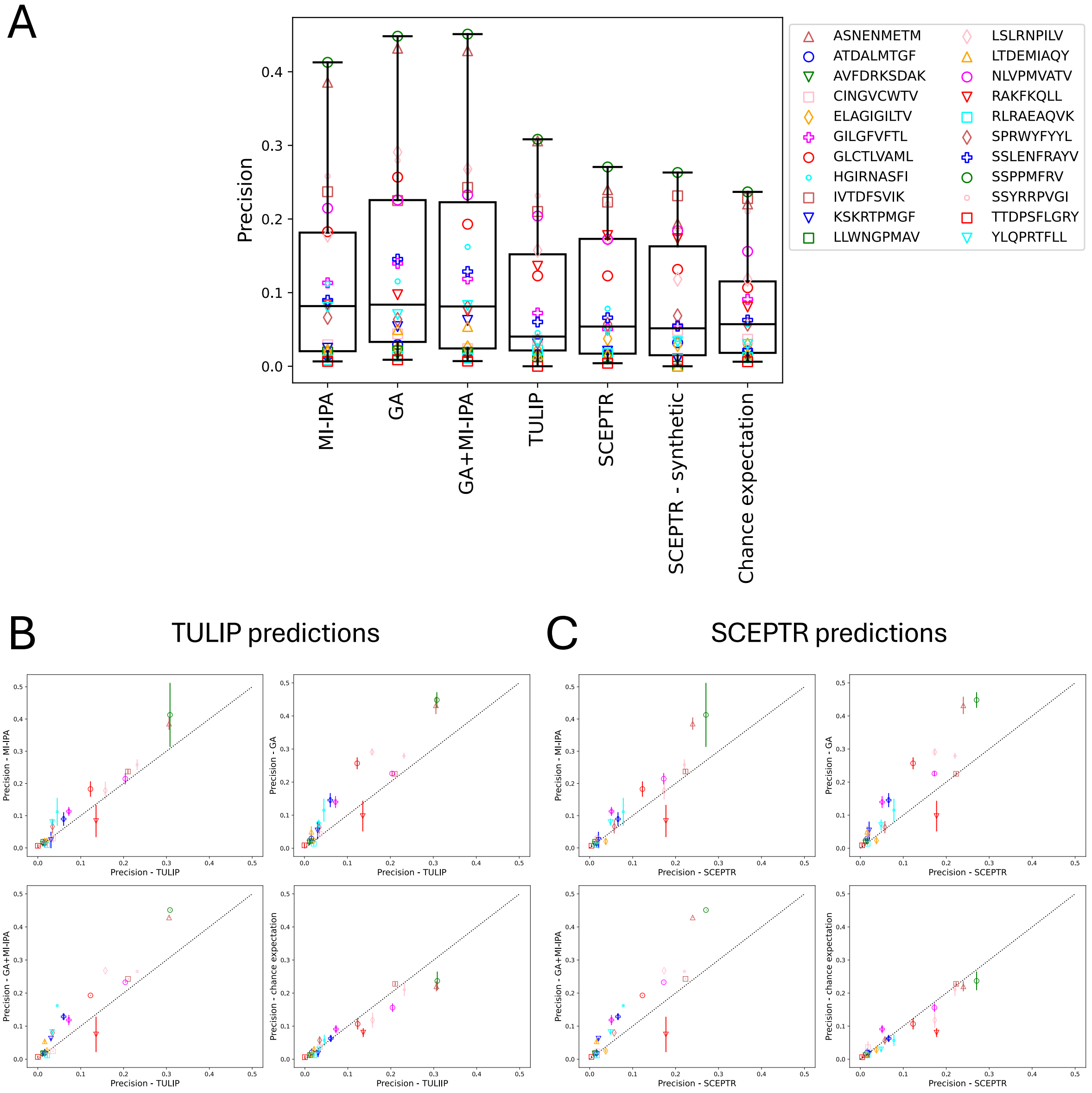


Figure S16: **Benchmarking pairing models against TULIP and SCEPTR. A)** Precision for each epitope in the six models evaluated plus the chance expectation (MI-IPA with $\lambda = 1$). SCEPTR was run both with its default model (trained on paired data) and with a model trained on synthetic data, in which the pairing in the training set was random (SCEPTR - synthetic). The edges of each boxplot show the quartiles, and the whiskers extend to 1.5 the interquartile range. Precision of prediction based on the TULIP (**B**) or SCEPTR (**C**) output compared to assigned by MI-IPA, GA and GA+MI-IPA algorithms and chance expectation (MI-IPA with $\lambda = 1$). Error bars indicate the standard deviation over the repeats of the models for MI-IPA, GA and GA+MI-IPA. The dotted line represents the diagonal.
