# Supplementary material for: Systematic analysis of CDR contacts and pairing constraints between T cell receptor αβ chains
Source: Bioinformatics. 2026 Jul 22;42(8):btag551. doi: 10.1093/bioinformatics/btag551 (PMC13447020; doi:10.1093/bioinformatics/btag551)
Supplement: Supplementary file 1 [file btag551_supplementary_data.zip › appendix.pdf]

Appendix

A1 Sequence inter-dependence between TCRα and β in epitope-specific repertoires

We retrieved paired-chain data from the VDJDb (Bagaev et al., 2020; Goncharov et al., 2022) and clustered all CDR3α and all CDR3β sequences from each epitope-specific repertoire based on triplet similarity (see Figure A1A for a representative example from epitope GLCTLVAML). Sequence similarity between CDR3s was assessed by normalised triplet similarity as previously described (Joshi et al., 2019). Briefly, each CDR3 is decomposed into sets of overlapping triplets. The number of triplets shared between two CDR3s is counted and normalised by $\sqrt{(L_1-1)(L_2-1)}$, where L_1 and L_2 are the lengths of the two CDR3s. Triplet similarity is calculated by using the stringdot function (norm = TRUE) of the kernlab package (Karatzoglou et al., 2004). The similarity function is called in Python by using package rpy2 (https://rpy2.github.io/) and networks are plotted using python-igraph (Csárdi & Nepusz, 2006). Triplet similarity was calculated on CDR3 sequences which did not include gaps. Sequence similarity graphs were defined by thresholding triplet similarity at a value of 0.76 and 0.72 for α and β respectively. The thresholds were set such that 99.99% of pairwise distances between CDR3 sequences not recognising the same epitope are below the threshold.

We observed no one-to-one relationship between clusters of α sequences and clusters of β sequences. However, within a single cluster of CDR3α sequences, the similarity of the paired CDR3β is higher than size-matched random subsamples of CDR3β sequences from that same repertoire (Figure A1B, median increase of 0.56), and similarly for CDR3β clusters (median increase of 0.15). Notably, 3 of 4 α clusters and 6 of 7 β clusters from epitope GLCTLVAML show higher similarity in paired sequences compared to background. We extended the analysis to all available epitopes for which at least 100 paired $\alpha\beta$ TCRs are in VDJDb (Figure A1C and Figure A2). We restricted the analysis to clusters of size 3 or greater. We detected no CDR3α clusters of size 3 or greater in epitopes LLWNGPMAV, SSYRRPVGI and TTDPSFLGRY and no CDR3β clusters of size 3 or greater in epitopes RLRAEAQVK and SPRWYFYYL. 15 of 22 and 17 of 22 epitopes showed increased similarity in CDR3α or β (of which 3 and 9 showed a significantly different distribution, respectively) associated with clusters of the other chain (Figure A2, median increase 0.096 for CDR3β paired to a CDR3α cluster and median increase 0.98 for CDR3α paired to a CDR3β cluster). Moreover, the pairwise similarities of CDR3α and CDR3β correlate for 2 of 22 and 5 of 22 epitopes, respectively (Figures A3 and A4). The increase in sequence similarity was variable for different epitopes. For epitope GLCTLVAML, for example, the CDR3α pairwise similarity increased by a median 0.56 (range = -0.02 - 0.93) and CDR3β median increases by 0.15 (range = 0 - 0.75). In contrast, epitope AVFDRKSDAK showed very little increased similarity (CDR3α median increase = 0.005, range = -0.14 - 0.36; CDR3β median increase = 0, range = 0 - 0.15).

Overall, these results suggest that, within an epitope-specific repertoire, similar TCRα chains

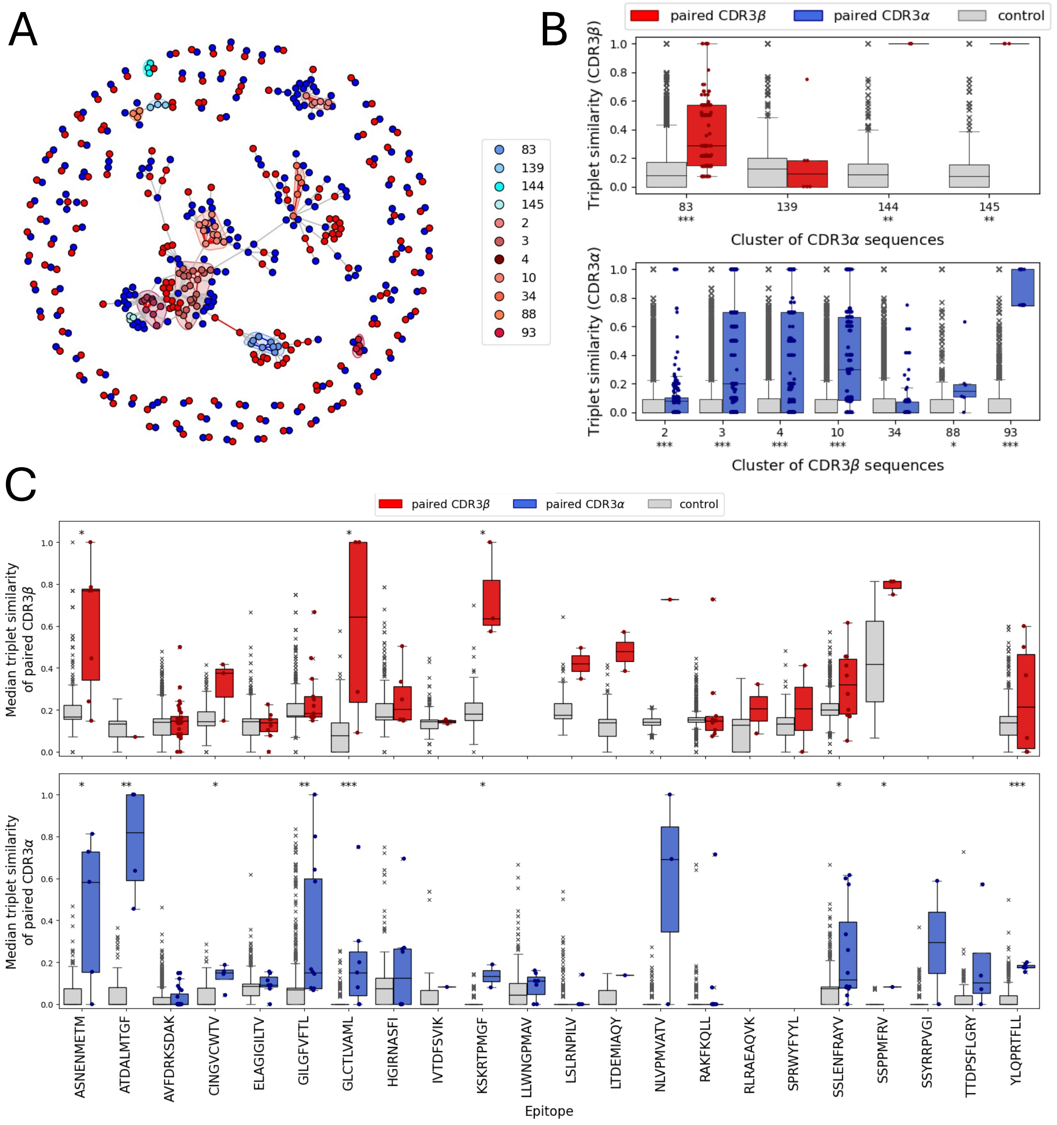

Figure A1: **Co-clustering of CDR3α and β sequences. A)** Each circle is a unique CDR3 sequence (α in blue, β in red). Similar α sequences are linked by blue edges, similar β sequences by red edges. Each α is linked to its paired β by a grey edge. The clusters analysed in **B** are coloured in shades of blue (α clusters) or red (β clusters) and identified by a shaded area around them. Each CDR3 sequence is represented only once, therefore the same CDR3β may be linked to multiple CDR3α (and vice versa), if it is not unique in the epitope-specific repertoire. **B)** Similarity of paired CDR3β when CDR3α are clustered (top, or vice versa - bottom) for the clusters identified in **A**. For each cluster, all pairwise similarities between the paired sequences are calculated (coloured boxplots). 100 equal-size random samples are taken as controls from the epitope-specific repertoire (grey boxplots). Only clusters of size 3 or greater are included. **C)** The analysis in **B** was repeated for 22 epitopes. Each dot represents the median for a cluster of size 3 or greater. Median similarity was also calculated for 100 controls for each cluster (grey boxes, random sequences drawn from the same epitope-specific repertoire). Columns are empty when no clusters of size 3 or larger are found. The edges of each boxplot show the quartiles, and the whiskers extend to 1.5 the interquartile range. The median of each boxplot is shown as a line. Outliers are shown as circles for real samples and crosses for the control subsamples. P-values (one-tailed Mann-Whitney U test with Bonferroni correction, comparing real to control with the alternative hypothesis of similarity in real > control): $*< 0.05$; $**< 0.01$; $***< 0.0001$.

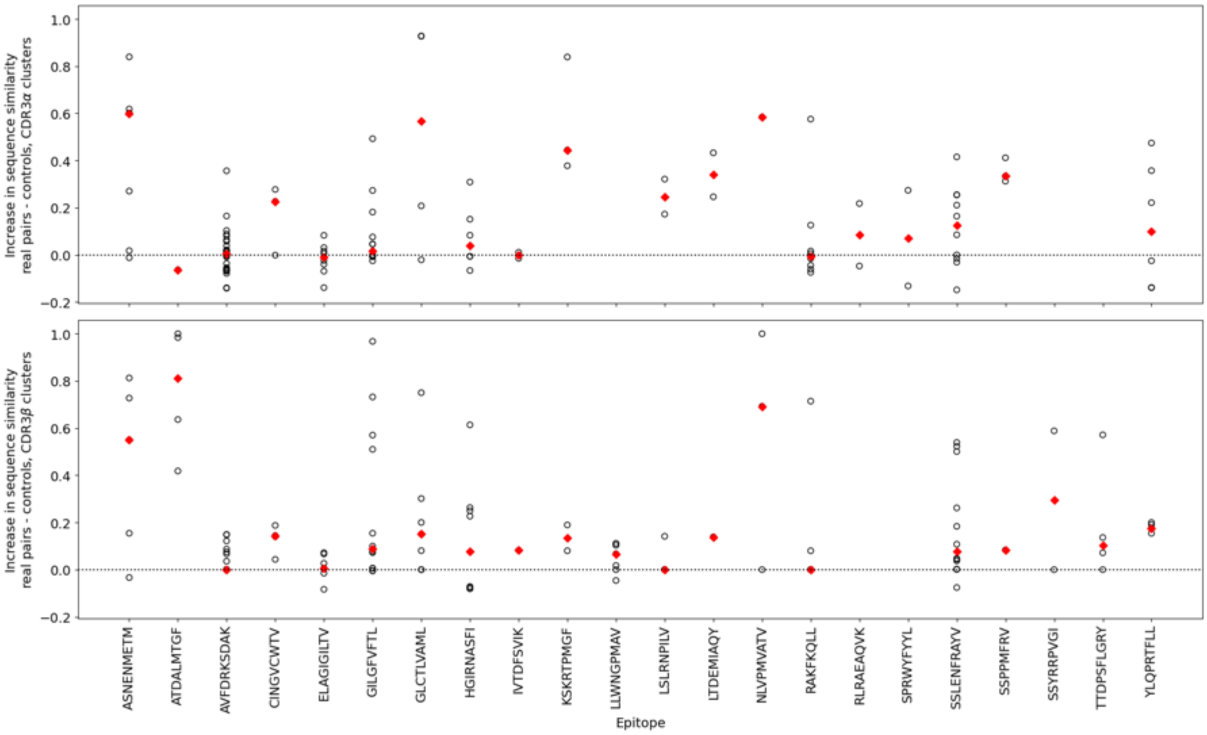

Figure A2: **Difference in sequence similarity between sequences paired to real clusters and random samples from epitope repertoires.** For each cluster (of CDR3α, top and of CDR3β, bottom) in each epitope repertoire, the similarity between the paired CDR3s to the sequences in each cluster is calculated. 100 random samples of the same size from the same epitope-specific repertoire are also taken and their similarity calculated. The difference between the median sequence similarity of the real pairs and of the random controls is shown (black circles, average median control similarity taken over all controls). The red diamonds show the average across all clusters for that epitope.

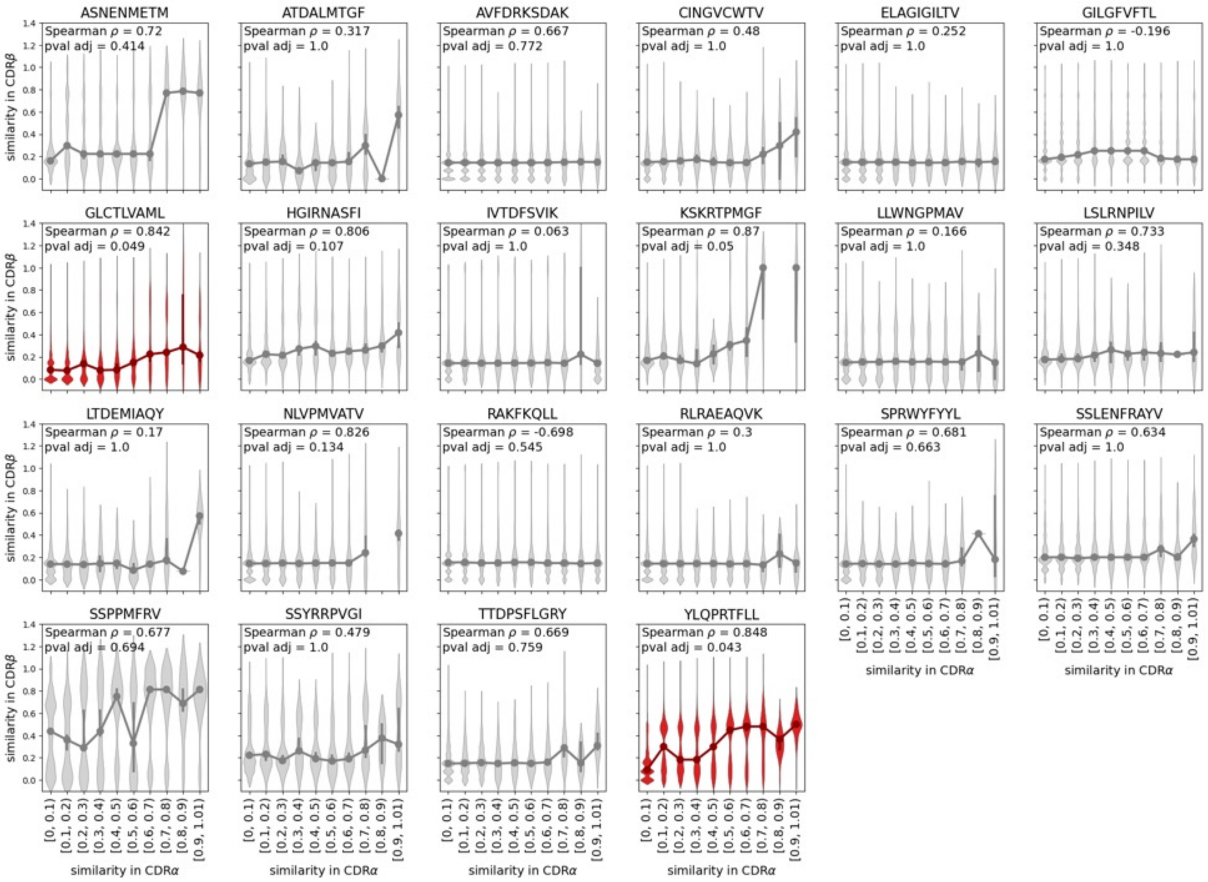

Figure A3: **Correlation of CDR3 pairwise sequence similarity between the two chains.** Pairwise similarity was calculated between all CDR3α and all CDR3β annotated for each epitope (including duplicate sequences). The pairwise similarity on the CDR3α was then binned and the similarity of the paired CDR3β examined. The large circles joined by a solid line represent the median for each distribution, whilst the violin plots show the distribution of the underlying distances. Spearman correlation was calculated using the mid-point of each bin, and correlating with the median of the values for that bin. Plots are red if the correlation is significant, grey if pvalue does not pass significance. p-value is calculated including Bonferroni correction.

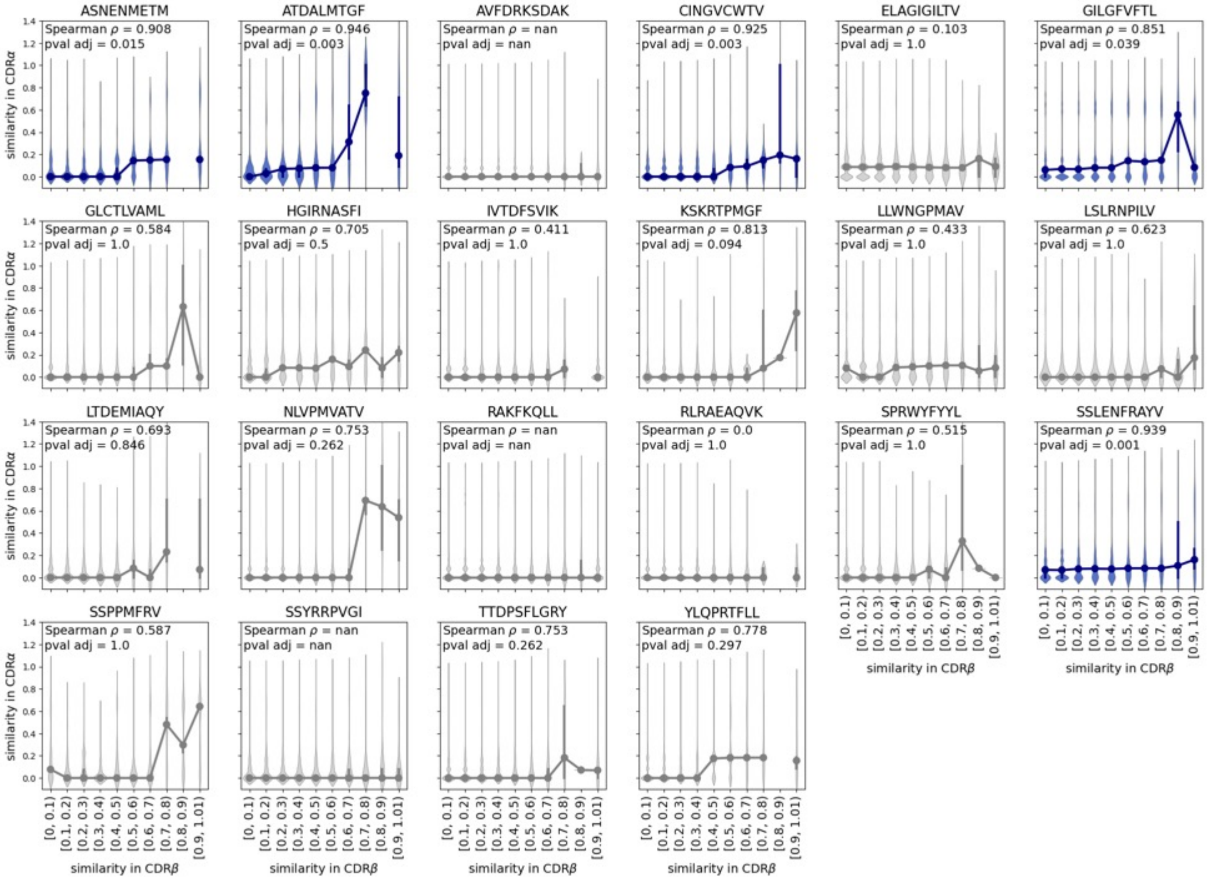

Figure A4: **Correlation of CDR3 pairwise sequence similarity between the two chains.** Pairwise similarity was calculated between all CDR3α and all CDR3β annotated for each epitope (including duplicate sequences). The pairwise similarity on the CDR3β was then binned and the similarity of the paired CDR3α examined. The large circles joined by a solid line represent the median for each distribution, whilst the violin plots show the distribution of the underlying distances. Spearman correlation was calculated using the mid-point of each bin, and correlating with the median of the values for that bin. Plots are blue if the correlation is significant, grey if pvalue does not pass significance. p-value is calculated including Bonferroni correction.

tend to pair with TCRβ chains that are more similar than random, i.e. TCR$\alpha\beta$ pairing is constrained.

A2 Calculating mutual information between TCR regions

MI is a statistical measure of the dependence of two random variables, i.e. of how much information each variable carries about the other. The MI between two regions where variability is moderate, such as V or J genes, can readily be estimated by using the observed frequencies of each gene in the set, together with their observed frequencies of co-occurrence. However, estimating the MI between one such region and a CDR3 sequence, or between two CDR3 sequences, is not as straightforward as most sequences occur only once (Table S1) and the data is thus insufficient to estimate the entropy of CDR3 sequences as a whole (and thus the MI involving them). We therefore focused on pairwise approximations of the CDR3 MI, where the amino acid sequence of the CDR3 region is considered one residue at a time, an approach that has been successfully used to study protein-protein interactions in the context of co-evolution (Dunn et al., 2008; Skerker et al., 2008; Bitbol, 2018; for an alternative approach using unbiased estimators for Renyi-Simpson entropies see Tiffeau-Mayer, 2023 and Henderson et al., 2024). The MI between a region of moderate variability (X) and each single residue position of the CDR3 sequence (S) was calculated, and then summed to obtain a pairwise approximation of the total MI between these two regions:

$$I(X;S) = \sum_{p=1}^{L} \sum_{r \in R} \sum_{x \in \Omega_X} f_{X,p}(x,r) \log \left(\frac{f_{X,p}(x,r)}{f_X(x) f_p(r)} \right) , \tag{1}$$

where f denotes a frequency observed in the data, L is the length of the CDR3 amino acid sequence of interest (we enforced $L = 19$ as described above), r is a residue at position p in sequence S, R is the set of possible residues (i.e. the 20 amino acids plus a character for a gap in alignment), and X is the random variable describing the moderately variable region of interest (e.g. the V gene), taking values x in an ensemble Ω_X. Similarly, a pairwise approximation of the MI between two CDR3 regions T and S was calculated by summing over all pairs of residue positions involving one position in each of these two regions:

$$I(T;S) = \sum_{q=1}^{L} \sum_{p=1}^{L} \sum_{r \in R} \sum_{w \in R} f_{q,p}(w,r) \log \left(\frac{f_{q,p}(w,r)}{f_q(w) f_p(r)} \right) , \tag{2}$$

where w is a residue at position q in sequence T (while r is a residue at position p in sequence S, as before).

Since plug-in estimates of MI are biased for small sample sizes (Nemenman et al., 2002; Archer et al., 2014), the 'real' value of the MI was extrapolated by taking sequential subsamples of size N [25, 35, 50, 80, 100, 150, 200, 300, 500, 1000, 1500, 2000, 2500, 3000, 5000, 10000, 15000] until sample size was reached. A regression line was fitted to the MI versus $1/N$ at

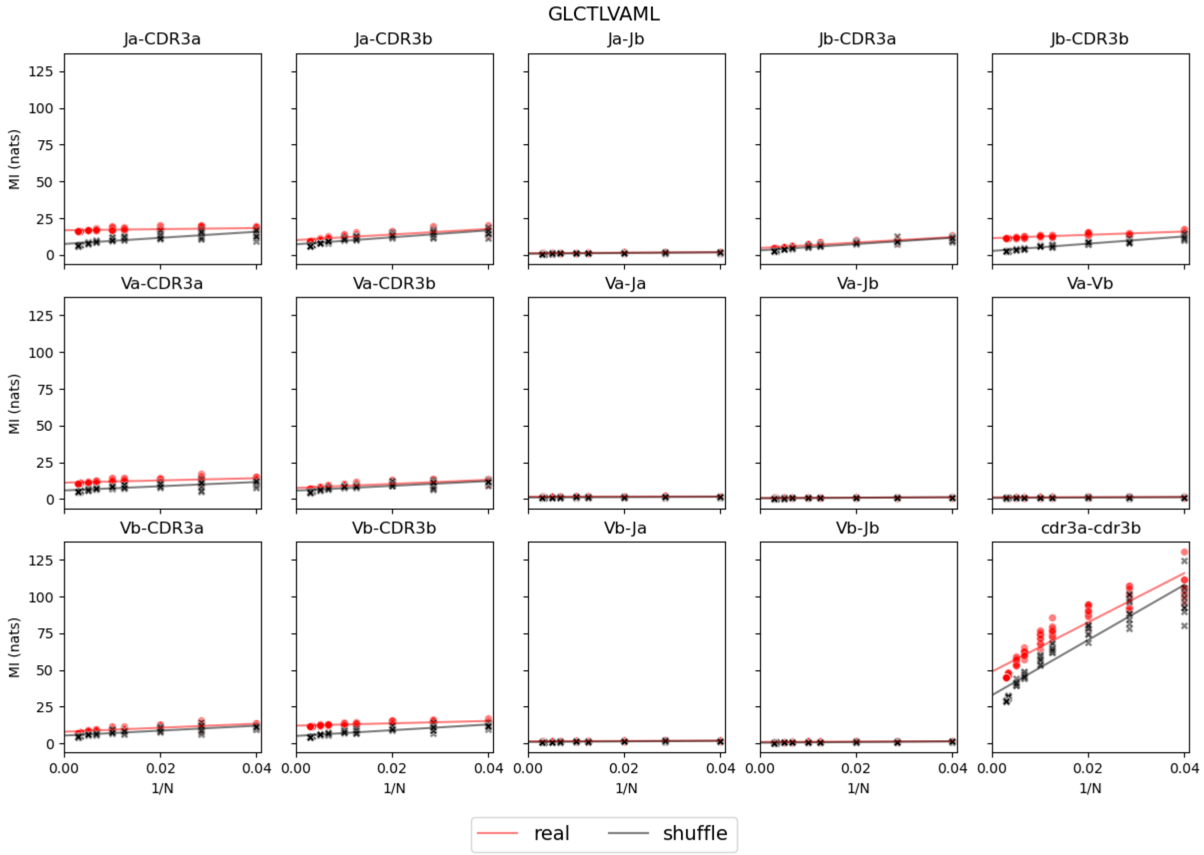

Figure A5: **Estimation of mutual information for epitope GLCTLVAML.** For each pair of TCR components (indicated in the title of each plot), mutual information (MI) was calculated at multiple subsamples for both the real set (red) and a shuffle (black). Each subsample was repeated 10 times. A line was fit through the point and MI was estimated as the y-intercept for both real set and shuffle.

each subsample and the y-intercept was used as the MI estimate ($\lim_{N\to\infty}$) (Strong et al., 1998; Panzeri et al., 2007). The procedure was repeated for both the real set and a shuffled version of the same set, where one variable is kept constant, whilst the second variable is randomly shuffled, so that the background entropy remains the same but the relationship between the two variables is broken. To account for batch effects that might be dependent on sequencing technology or study design, we perform the shuffle only within the sequences belonging to a single study (thus maintaining any batch effects on the signal). Each subsampling was repeated 10 times. Figure A5 shows the estimation for epitope GLCTLVAML as an example.

A3 Implementation of pairing models

Mutual information iterative pairing algorithm (MI-IPA)

The mutual information-based iterative pairing algorithm (MI-IPA Bitbol, 2018) aims to correctly match pairs of interacting proteins from two input lists based on the MI between the

respective amino acid sequences, by permuting the pairing between the two lists to approximately maximise MI. The method exploits the observation that, in families of evolutionarily related interacting proteins, the interacting contact regions between the proteins will co-evolve, such that a change in sequence of one member of the pair will constrain the possible compensatory changes in the other member of the pair, in order to preserve the required functional interaction. We adapted the method to pair a set of unpaired TCRα/β sequences and implemented it in Python v3.11.0, parallelising both the scoring of potential pairs and repeats over multiple CPU cores. Correct pairings were known but blinded to the algorithm.

The original implementation of the MI-IPA used species to reduce the combinatorial number of assignments to be evaluated. As species are not available in this case, we used study and individual information as available from VDJDb as a substitute. As repeats are present both in the TCRα and TCRβ, randomness was added into the model at the assignment stage. To account for this, as well as the influence of the initial random pairing, we ran the model 10 times in each condition.

Parameters θ (sequence diversity threshold) and λ (pseudocount) were defined as in Bitbol, 2018 and used to correct the frequency calculation for MI estimation. Step size and confidence calculation were also optimised for the dataset. We compared the Hungarian scoring method, a greedy assignment based on confidence calculation, and a 'no confidence' scenario, where the greedy assignment is performed directly on the scores assigned to each pair, without calculating a confidence score (Figure A6). We run the MI-IPA with diversity threshold $\theta = 0.6$, pseudocount $\lambda = 0.6$, step size of 6 sequences and no confidence calculation. Equivalent settings but with $\lambda = 1$ were used as a negative control yielding the chance expectation (as $\lambda = 1$ prevents the algorithm from learning).

Graph alignment (GA) algorithm

The graph alignment algorithm (GA) aims to align two sequence similarity networks built on the lists of sequences to be paired (Bradde et al., 2010). The method assumes that two co-evolving protein families will have similar phylogenetic trees (in our use-case, similarity graphs), and thus tries to align the two trees to pair the family members. First, all pairwise $\alpha - \alpha$ and $\beta - \beta$ CDR3 distances in each input list are calculated. The distances are then used to calculate the two similarity networks and the generated graphs are aligned by trying to maximise the number of overlapping edges. A published Julia implementation of the GA (Gandarilla-Pérez et al., 2023, https://github.com/carlosgandarilla/GA-IPA) was integrated in our Python v3.11.0 pipeline with no amendments to the code that performs the alignment using PyJulia v0.6.0 (https://pyjulia.readthedocs.io/en/latest/).

Four different distance measures were used: (1) Levenshtein (or edit) distance, (2) Weighted Levenshtein distance (where substitutions are weighted 1 and gaps are weighted $1 + \ln(4)$), (3) Triplet distance (calculated as 1-triplet similarity, as described above), (4) TCRdist (Dash et al., 2017) calculated on the CDR3 sequence only. Levenshtein and weighted Levenshtein

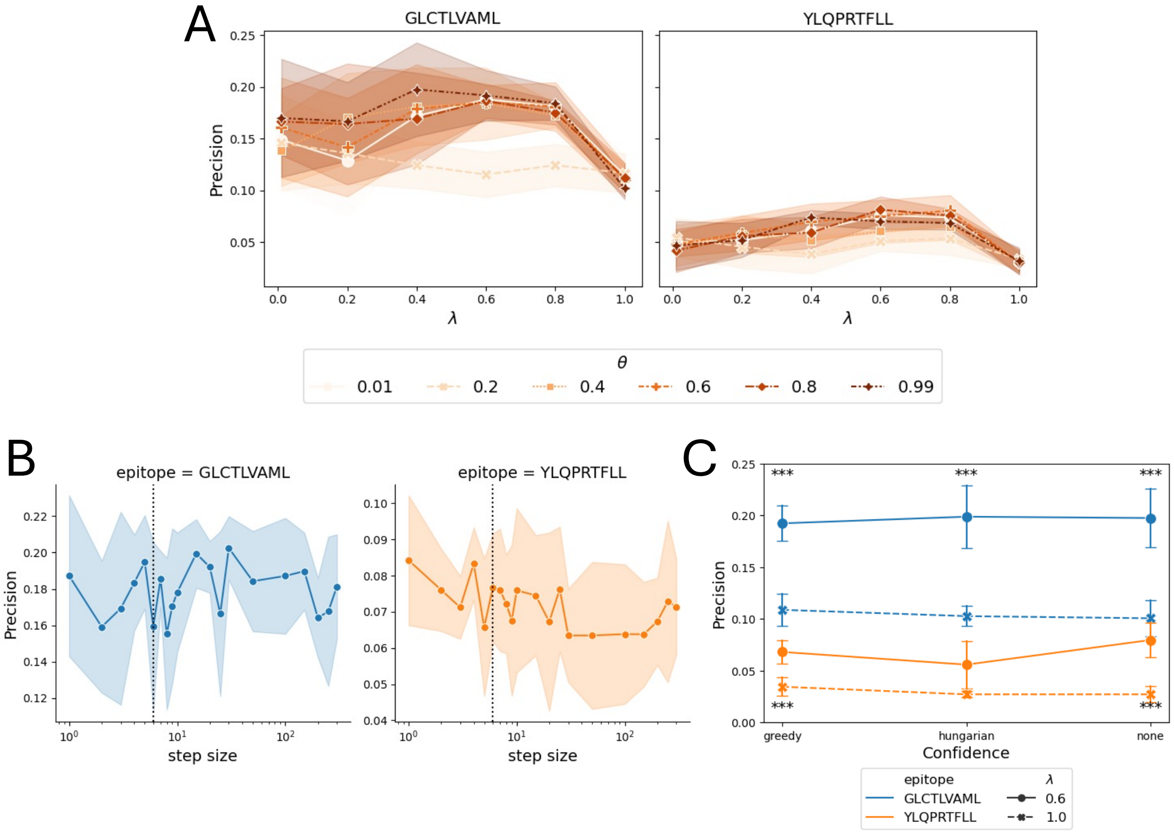

Figure A6: **Optimisation of parameters for MI-IPA.** All parameters are as in Bitbol, 2018, except that we add a scenario where no 'confidence' is calculated. This is a greedy assignment (as in "greedy"), but no confidence score is calculated for the pairing. Rather, the scoring for each pairing is used raw to rank the pairs. Shaded areas or error bars represent the standard deviation around the mean (solid line) for 10 repeats of each model. **A)**: θ and λ (controlling sampling correction and effective set size calculation, respectively); **B)**: step size; **C)**: confidence calculation algorithms. One-sided t-tests are calculated to compare performance of each model to its no-learning scenario (achieved by setting $\lambda = 1$). pvalues are corrected with Bonferroni correction and indicated as follows: $***< 0.001$; $0.001 \leq ** < 0.01$; $0.01 \leq * < 0.05$.

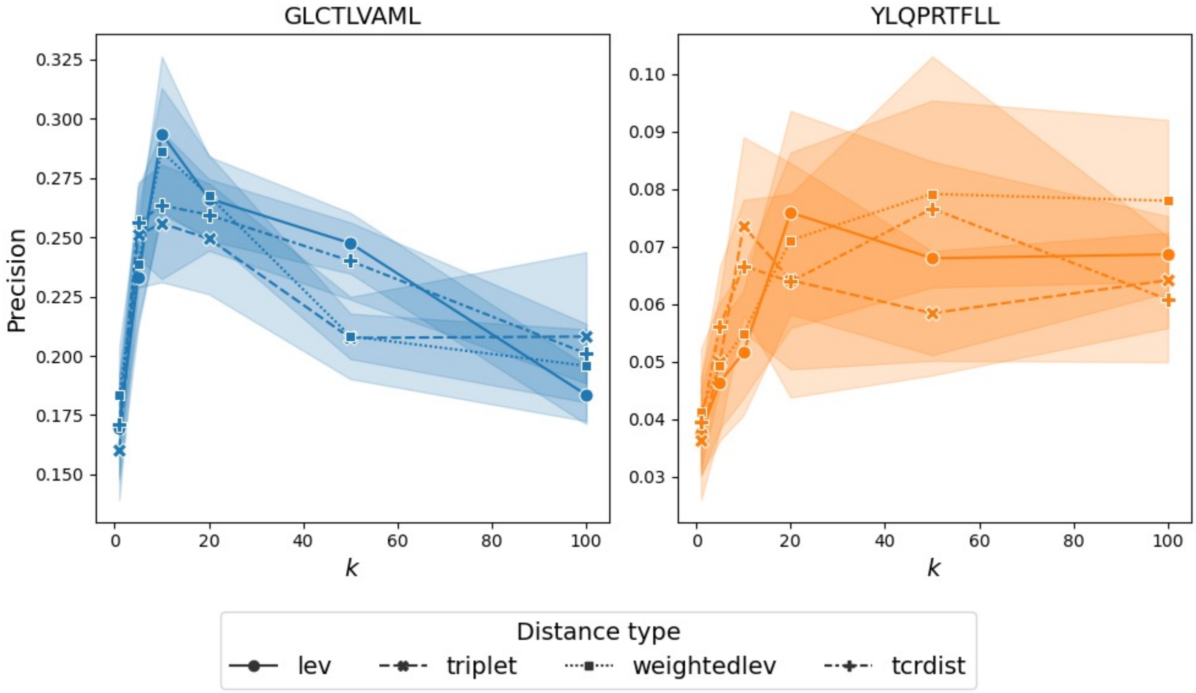

Figure A7: **Impact of k and distance function choice on GA pairing.** The GA was run with a range of k and 4 different distance metrics to evaluate their impact on the two benchmarking epitopes. Precision was calculated for each setting. The shaded areas show the standard deviation around the mean (solid line) for 100 repeats of the model.

distance are implemented in rapidfuzz (https://pypi.org/project/rapidfuzz/ Bachmann, 2024), whilst TCRdist is calculated using the implementation available in package pwseqdist (https://github.com/agartland/pwseqdist), using the parameters specified within tcrdist3 (https://tcrdist3.readthedocs.io/en/latest/, Mayer-Blackwell et al., 2021).

We tested the various distance metrics and a range of number of neighbours (k, defined as in the original method) to achieve the best pairing (Figure A7). The GA was run using Levenshtein distance and $k = 20$.

Combining GA and MI-IPA (GA+MI-IPA)

We combined the GA and MI-IPA as proposed by Gandarilla-Pérez et al., 2023. In the original implementation, the GA was used to calculate a first pairing for the sequences of interest. The stable assignments (i.e. the pairs that were consistently assigned over many iterations of the GA) were then used as a golden set for the MI-IPA (i.e. a training set of pairs that are used to initialise the MI-IPA and kept as they are throughout the IPA). Here, we explored three different ways of combining GA with MI-IPA:

(1) the most stable pairs (selected $\geq 95\%$ of the time, or the top 5 pairs, whichever is largest) from the GA are used as a golden set for the MI-IPA and removed from the set of pairs to be paired by the MI-IPA (as in Gandarilla-Pérez et al., 2023);

Epitope	Ties in total set	Number of pairs	Ties in stable pairs	Number of stable pairs
**ASNENMETM**	0	196	0	78
**ATDALMTGF**	5	125	0	5
**AVFDRKSDAK**				
subsample 0	72	700	0	5
subsample 1	49	700	0	9
subsample 2	39	700	0	5
subsample 3	68	700	0	17
subsample 4	71	700	0	12
**CINGVCWTV**	4	226	0	5
**ELAGIGILTV**	29	374	0	5
**GILGFVFTL**				
subsample 0	51	700	0	10
subsample 1	51	700	0	11
subsample 2	35	700	0	85
subsample 3	42	700	0	35
subsample 4	42	700	0	12
**GLCTLVAML**	11	342	0	84
**HGIRNASFI**	7	243	0	5
**IVTDFSVIK**	34	704	0	107
**KSKRTPMGF**	4	98	0	5
**LLWNGPMAV**	6	235	0	5
**LSLRNPILV**	1	127	0	80
**LTDEMIAQY**	6	124	0	5
**NLVPMVATV**	0	348	0	88
**RAKFKQLL**				
subsample 0	65	700	0	5
subsample 1	79	700	0	5
subsample 2	49	700	0	5
subsample 3	137	700	0	5
subsample 4	70	700	0	5
**RLRAEAQVK**	50	412	0	5
**SPRWYFYYL**	5	175	0	5
**SSLENFRAYV**	6	349	0	10
**SSPPMFRV**	3	133	0	94
**SSYRRPVGI**	0	177	0	101
**TTDPSFLGRY**	25	242	0	5
**YLQPRTFLL**	23	331	0	5

Table A1: **Number of pairs affected by random selection of modal β.** For each epitope, we show the number of pairs where a scoring tie occurs amongst all pairs ('total set') and amongst the pairs deemed stable ('stable pairs').

(2) the most stable pairs (selected $\geq 95\%$ of the time, or the top 5 pairs, whichever is largest) from the GA are used as a golden set for the MI-IPA but they are not removed from the set of pairs that the MI-IPA tries to assign. This allows the MI-IPA to correct the pairs in the final assignments;

(3) the complete consensus assignment from the GA (i.e. the TCRβ that is most often assigned for each TCRα across 100 repeats of the GA) is used as an input training set to the MI-IPA, but all α and all β are also available for re-pairing by the MI-IPA.

Note that when multiple β are chosen the same number of times for an α in the GA, the β included in the stable assignment is chosen at random. This never occurs with settings (1) and (2), as the most stable pairs always have a clear mode. In setting (3), the number of pairs where the beta needs to be randomly selected ranges between 0 and 20% of total pairs (Table A1). The final assignments for all pairs are then extracted and evaluated against the known pairs. We ran the GA+MI-IPA with the regime described in (2) (Figure A8).

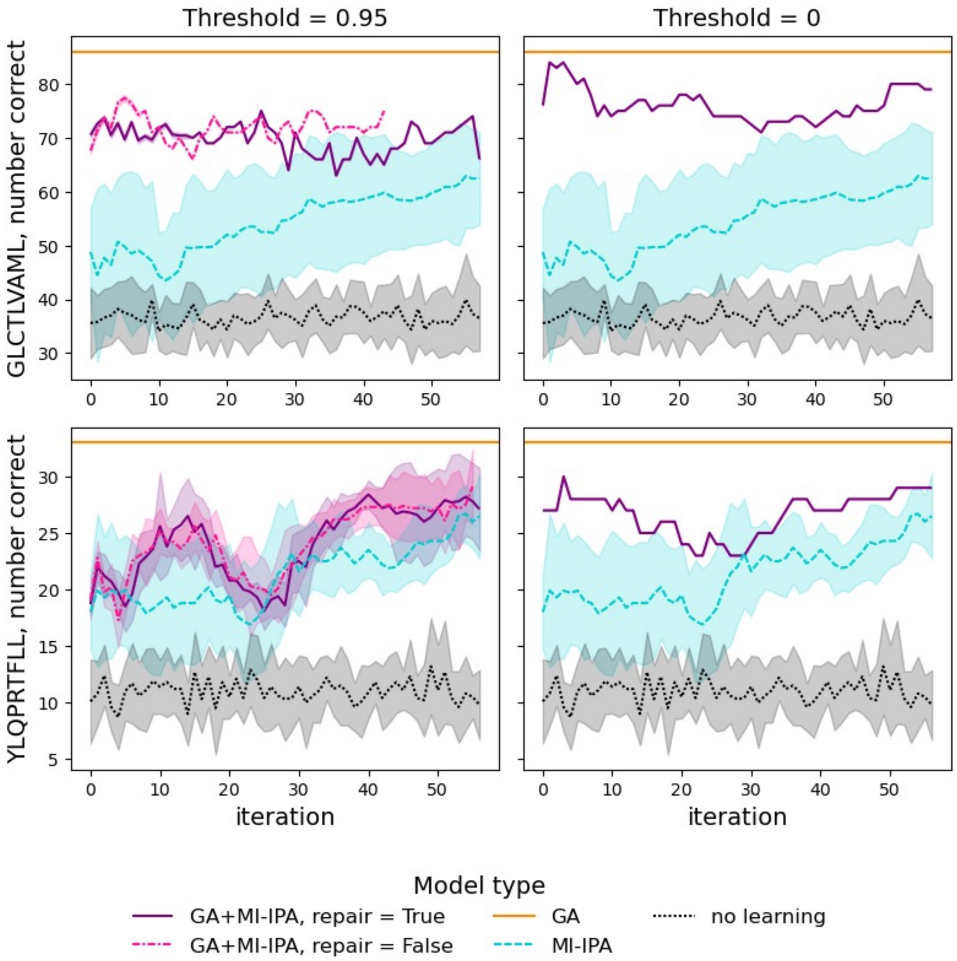

Figure A8: **Learning of the GA+MI-IPA over iterations with different settings.** Performance of combining the GA (Levenshtein distance, $k = 20$) with MI-IPA ($\theta = 0.6$, no confidence, step size = 6) is shown for the two benchmarking epitopes. The orange horizontal line shows the number of correct pairs in the GA consensus assignment which was used to select the training sets. The GA assignment is used as training for the MI-IPA, either by taking the complete assignment (threshold = 0, right column), or only the stable pairs (threshold = 0.95, left column). The MI-IPA is then allowed to pair all remaining β to all remaining α (pink dotted line with fewer iterations) or pair all α and β, including the ones in the training set (purple line). Performance of the MI-IPA on its own is also shown, with $\lambda = 0.6$ (cyan line) or $\lambda = 1$ (no learning, black dotted line). Shaded areas show the standard deviation around the mean (solid line).

References

Archer, E., Park, I. M., & Pillow, J. (2014, April). Bayesian Entropy Estimation for Countable Discrete Distributions [arXiv:1302.0328 [cs, math]]. https://doi.org/10.48550/arXiv.1302.0328

Bachmann, M. (2024, April). Rapidfuzz/RapidFuzz: Release 3.8.1. https://doi.org/10.5281/zenodo.10938887

Bagaev, D. V., Vroomans, R. M., Samir, J., Stervbo, U., Rius, C., Dolton, G., Greenshields-Watson, A., Attaf, M., Egorov, E. S., Zvyagin, I. V., Babel, N., Cole, D. K., Godkin, A. J., Sewell, A. K., Kesmir, C., Chudakov, D. M., Luciani, F., & Shugay, M. (2020). VDJdb in 2019: Database extension, new analysis infrastructure and a T-cell receptor motif compendium [Publisher: Oxford University Press]. *Nucleic Acids Research*, *48*(D1), D1057–D1062. https://doi.org/10.1093/nar/gkz874

Bitbol, A.-F. (2018). Inferring interaction partners from protein sequences using mutual information (C. O. Wilke, Ed.). *PLOS Computational Biology*, *14*(11), e1006401. https://doi.org/10.1371/journal.pcbi.1006401

Bradde, S., Braunstein, A., Mahmoudi, H., Tria, F., Weigt, M., & Zecchina, R. (2010). Aligning graphs and finding substructures by a cavity approach [arXiv:0905.1893 [cond-mat, q-bio]]. *EPL (Europhysics Letters)*, *89*(3), 37009. https://doi.org/10.1209/0295-5075/89/37009

Csárdi, G., & Nepusz, T. (2006). The igraph software package for complex network research. *InterJournal*, *Complex Systems*, Complex Systems. Retrieved May 2, 2023, from https://igraph.org

Dash, P., Fiore-Gartland, A. J., Hertz, T., Wang, G. C., Sharma, S., Souquette, A., Crawford, J. C., Clemens, E. B., Nguyen, T. H. O., Kedzierska, K., La Gruta, N. L., Bradley, P., & Thomas, P. G. (2017). Quantifiable predictive features define epitope-specific T cell receptor repertoires [Publisher: Nature Publishing Group]. *Nature*, *547*(7661), 89–93. https://doi.org/10.1038/nature22383

Dunn, S., Wahl, L., & Gloor, G. (2008). Mutual information without the influence of phylogeny or entropy dramatically improves residue contact prediction. *Bioinformatics*, *24*(3), 333–340. https://doi.org/10.1093/bioinformatics/btm604

Gandarilla-Pérez, C. A., Pinilla, S., Bitbol, A.-F., & Weigt, M. (2023). Combining phylogeny and coevolution improves the inference of interaction partners among paralogous proteins [Publisher: Public Library of Science]. *PLOS Computational Biology*, *19*(3), e1011010. https://doi.org/10.1371/journal.pcbi.1011010

Goncharov, M., Bagaev, D., Shcherbinin, D., Zvyagin, I., Bolotin, D., Thomas, P. G., Minervina, A. A., Pogorelyy, M. V., Ladell, K., McLaren, J. E., Price, D. A., Nguyen, T. H., Rowntree, L. C., Clemens, E. B., Kedzierska, K., Dolton, G., Rius, C. R., Sewell, A., Samir, J., . . . Shugay, M. (2022). VDJdb in the pandemic era: A compendium of T cell receptors specific for SARS-CoV-2 [Publisher: Nature Publishing Group]. *Nature Methods 2022 19:9*, *19*(9), 1017–1019. https://doi.org/10.1038/s41592-022-01578-0

Henderson, J., Nagano, Y., Milighetti, M., & Tiffeau-Mayer, A. (2024). Limits on inferring t cell specificity from partial information [Publisher: Proceedings of the National Academy of Sciences]. *Proceedings of the National Academy of Sciences*, *121*(42), e2408696121. https://doi.org/10.1073/pnas.2408696121

Joshi, K., Robert de Massy, M., Ismail, M., Reading, J. L., Uddin, I., Woolston, A., Hatipoglu, E., Oakes, T., Rosenthal, R., Peacock, T., Ronel, T., Noursadeghi, M., Turati, V., Furness, A. J. S., Georgiou, A., Wong, Y. N. S., Ben Aissa, A., Werner Sunderland, M., Jamal-Hanjani, M., . . . Chain, B. (2019). Spatial heterogeneity of the T cell receptor repertoire reflects the mutational landscape in lung cancer. *Nature Medicine*, *25*(1549), 1559. https://doi.org/10.1038/s41591-019-0592-2

Karatzoglou, A., Smola, A., Hornik, K., & Zeileis, A. (2004). Kernlab - An S4 package for Kernal Methods in R [ISBN: 9783037851579]. *Journal of Statistical Software*, *11*(9). https://doi.org/10.4028/www.scientific.net/AMR.271-273.389

Mayer-Blackwell, K., Schattgen, S., Cohen-Lavi, L., Crawford, J. C., Souquette, A., Gaevert, J. A., Hertz, T., Thomas, P. G., Bradley, P., & Fiore-Gartland, A. (2021). TCR meta-clonotypes for biomarker discovery with tcrdist3 enabled identification of public, HLA-restricted clusters of SARS-CoV-2 TCRs (B. Chain, A. M. Walczak, B. Chain, & T. Ronel, Eds.) [Publisher: eLife Sciences Publications, Ltd]. *eLife*, *10*, e68605. https://doi.org/10.7554/eLife.68605

Nemenman, I., Shafee, F., & Bialek, W. (2002, January). Entropy and inference, revisited [arXiv:physics/0108025]. https://doi.org/10.48550/arXiv.physics/0108025

Panzeri, S., Senatore, R., Montemurro, M. A., & Petersen, R. S. (2007). Correcting for the sampling bias problem in spike train information measures. *Journal of Neurophysiology*, *98*(3), 1064–1072. https://doi.org/10.1152/JN.00559.2007

Skerker, J. M., Perchuk, B. S., Siryaporn, A., Lubin, E. A., Ashenberg, O., Goulian, M., & Laub, M. T. (2008). Rewiring the Specificity of Two-Component Signal Transduction Systems [Publisher: Elsevier]. *Cell*, *133*(6), 1043–1054. https://doi.org/10.1016/j.cell.2008.04.040

Strong, S. P., Koberle, R., De Ruyter Van Steveninck, R. R., & Bialek, W. (1998). Entropy and Information in Neural Spike Trains. Retrieved April 7, 2023, from https://arxiv.org/abs/cond-mat/9603127

Tiffeau-Mayer, A. (2023, October). Unbiased estimation of sampling variance for Simpson's diversity index [arXiv:2310.03439 [cond-mat, q-bio]]. https://doi.org/10.48550/arXiv.2310.03439
